# Comparative transcriptomics of broad‐spectrum and synthetic cannabidiol treated C2C12 skeletal myotubes

**DOI:** 10.14814/phy2.70059

**Published:** 2024-09-17

**Authors:** Scott H. Gillham, Paige L. Cole, Mark R. Viggars, Andy H. Nolan, Graeme L. Close, Daniel J. Owens

**Affiliations:** ^1^ Research Institute of Sport and Exercise Science (RISES) Liverpool John Moores University Liverpool UK; ^2^ Department of Physiology and Aging University of Florida Gainesville Florida USA; ^3^ Centre for Tumour Biology, Barts Cancer Institute Queen Mary University of London London UK

**Keywords:** cannabidiol, skeletal muscle, transcriptome

## Abstract

Cannabidiol (CBD) is widely used in sports for recovery, pain management, and sleep improvement, yet its effects on muscle are not well understood. This study aimed to determine the transcriptional response of murine skeletal muscle myotubes to broad‐spectrum CBD and synthetic CBD (sCBD). Differentiated C2C12 myotubes were treated with 10 μM CBD, sCBD, or vehicle control (DMSO) for 24 h before RNA extraction. Poly‐A tail‐enriched mRNA libraries were constructed and sequenced using 2 × 50 bp paired‐end sequencing. CBD and sCBD treatment induced 4489 and 1979 differentially expressed genes (DEGs; *p* < 0.001, FDR step‐up <0.05), respectively, with common upregulation of 857 genes and common downregulation of 648 genes. Common upregulated DEGs were associated with “response to unfolded protein,” “cell redox homeostasis,” “endoplasmic reticulum stress,” “oxidative stress,” and “cellular response to hypoxia.” Common downregulated DEGs were linked to “sarcomere organization,” “skeletal muscle tissue development,” “regulation of muscle contraction,” and “muscle contraction.” CBD treatment induced unique DEGs compared to sCBD. The data indicate CBD may induce mild cellular stress, activating pathways associated with altered redox balance, unfolded protein response, and endoplasmic reticulum stress. We hypothesize that CBD interacts with muscle and may elicit a “mitohormetic” effect that warrants further investigation.

## 1 INTRODUCTION

Cannabidiol (CBD) is one of many cannabinoids derived from the *cannabis sativa* plant, which produces cannabinoids in addition to more than 550 other compounds (Capano et al., 2020; Puntel et al., 2016). Regarding cannabinoids, there have been >140 elucidated (Ujváry & Hanuš, 2016), with two of the most well‐known being ∆^9^‐tetrahydrocannabinol (∆^9^‐THC) and CBD (Brunt & Bossong, 2020; Gaoni & Mechoulam, 1964). The former is best known for its intoxicating properties, whilst CBD has shown little intoxicating or abuse potential (Sekar & Pack, 2019). These exogenous phytocannabinoids have been suggested to exert their action via their ability to bind to components of the body's endocannabinoid system (ECS). The ECS, primarily comprises cannabinoid receptors 1 (CB1R), and 2 (CB2R) and endogenous cannabinoids anandamide (AEA) and 2‐aracidonoylglycerol (2‐AG) (Lu & Mackie, 2020).

Cannabidiol use is prevalent in professional sports and is reportedly used for improving recovery, managing perceived pain and improving sleep, with 68% of players reporting a perceived benefit (Kasper et al., 2020). Others have reported similar findings for the use of cannabis, with one large meta‐analysis reporting that in a sample of >46,000 athletes, ~ 23% admitted to having used some form of cannabis in the past year (Docter et al., 2020). Despite the use of CBD by athletes as an alternative to opioids (Vernec et al., 2017) and nonsteroidal anti‐inflammatory drugs (NSAIDs) (Schoenfeld, 2012), there is a scarcity of evidence for any interaction between CBD and muscle. There is evidence that the cannabinoid receptors CBR1 and CBR2 gene (Cavuoto et al., 2007; Haddad, 2021) and protein are expressed in human, mouse, and rat skeletal muscle (Crespillo et al., 2011; Dalle & Koppo, 2021; Kalkan et al., 2023; Mendizabal‐Zubiaga et al., 2016); however, the latter is limited to immunolabeling studies. Antibody specificity and absolute quantification of the receptors by mass spectrometry have not yet been performed to confirm abundance in human skeletal muscle.

In vitro studies on skeletal myoblasts demonstrate no effect of a hemp‐derived CBD isolate (99.8% purity) on anabolic signaling through mTORC1 or inflammatory signaling through nuclear factor kappa B, as well as no CB1 receptor expression (Langer et al., 2022). However, others provide evidence that synthetic CBD (sCBD) may be promyogenic, an effect attributed to increasing [Ca^2+^], mostly via TRPV1 activation (Iannotti et al., 2019). Similar observations have been made in primary satellite cells and myoblasts isolated from healthy and/or Duchenne muscular dystrophy (DMD) donors. In vivo, sCBD administration confers anti‐inflammatory properties and protects skeletal muscle against degeneration in *mdx* mice (Iannotti et al., 2019). In a separate study on healthy rats undergoing eccentric loading of the tibialis anterior, administration of hemp‐derived CBD isolate decreased pro‐inflammatory signaling without blunting the anabolic response to exercise in rats (Langer et al., 2021). As such, the limited available evidence points toward a promyogenic and anti‐inflammatory effect on skeletal muscle.

Whilst CBD is thought to exert its effects via the ECS, it cannot be dismissed that the many other compounds found in the cannabis plant could contribute toward the effects of broad‐spectrum CBD. Indeed, untargeted and targeted metabolomics of cannabis cultivars identified a total of 377 analytes after controlling for pesticides (Wishart et al., 2024). The most abundant analytes included lipids and lipid‐like molecules and organic acids and their derivatives. Notably, the cultivars all contained high amounts of polyphenols including catechin and several other quantifiable cannabinoids. Given that polyphenols, for example, have been extensively studied in the context of exercise‐induced muscle damage, inflammation, and repair, with many studies reporting positive effects (Rickards et al., 2021), it is important to dissect where muscle‐specific effects of CBD exposure might be derived.

Taken together, this preliminary investigation aimed to determine the global transcriptional response of C2C12 skeletal muscle myotubes following exposure to broad‐spectrum CBD and sCBD. We aimed to ascertain if (1) skeletal muscle is responsive to broad‐spectrum CBD and sCBD and (2) whether these effects are attributable solely to CBD or the vast array of other compounds in commercially available broad‐spectrum CBD. We hypothesized that both commercially available broad‐spectrum CBD and sCBD would induce significant transcriptional changes in skeletal muscle myotubes related to metabolism, myogenesis, and inflammatory signaling based on the findings of others. Furthermore, we hypothesized that there would be a significant number of differentially expressed genes that are unique to broad‐spectrum CBD treatment when compared with sCBD.

## 2 MATERIALS AND METHODS

### 2.1 Chemicals, reagents and Plasticware

THC‐free, broad‐spectrum, hemp‐derived CBD was provided by Naturecan Ltd. as crystalline CBD. The CBD was dissolved in DMSO as a stock concentration of 36 mg mL^−1^. Pure sCBD powder was provided by Pureis Ltd. The sCBD was reconstituted in DMSO as a stock concentration at 36 mg·mL^−1^. Reconstituted CBD and sCBD were stored at −20°C and used within 3 weeks of reconstitution. When required, CBD and sCBD stock solutions were diluted further in culture media for the experiments outlined herein.

Dulbecco's modified eagle medium (DMEM. Cat. ID. 11,965,092), fetal bovine serum (FBS. Cat. ID. A5256801), new‐born calf serum (NBCS. Cat. ID. 16,010,159), penicillin–streptomycin (pen‐strep. Cat. ID. 15,070,063), and horse serum (HS. Cat. ID. 16,050,122) were purchased from Thermo Fisher Scientific (Oxford, UK). Phosphate buffered saline tablets (PBS. Cat. ID. 524,650), DMSO (Cat. ID. D2438‐50ML), trypsin–EDTA (Cat. ID. T4049), MTT (Cat. ID. 475,989), propidium iodide (Cat. ID. 537,059), and gelatin from porcine skin (Cat. ID. G1890) were purchased from Sigma Aldrich (Gillingham, UK). T75 culture flasks and 6 well culture plates were purchased from Nunc (Thermo Fisher Scientific, Oxford, UK).

C2C12 myoblasts were purchased from ATCC (Cat. ID. CRL‐1772. LGC Standards, Middlesex, UK). All experiments detailed in this manuscript were performed on C2C12s between passage 5 and 10. For the isolation of high‐quality RNA, RNeasy isolation kits (Cat. ID. 74104) were purchased from Qiagen (Qiagen Ltd. Manchester, UK).

### 2.2 Cell culture

C2C12 murine myoblasts were cultured on gelatin (0.2%) coated 6‐well culture plates in humidified 5% CO_2_ at 37°C in growth media comprising DMEM, 10% FBS, 10% NBCS, and 1% of a pen‐strep solution. Upon reaching ~80% confluence, monolayers were washed twice with prewarmed PBS and switched to low serum differentiation media (DM), DMEM, 2% HS, and 1% pen‐strep. Every 48 h thereafter, DM was removed from monolayers via aspiration and was replaced with fresh media. Cells were terminally differentiated by day 8 of low serum DM exposure.

### 2.3 Dose tolerability experiments

To ascertain an appropriate dose that would maximize the signal but minimize cytotoxicity to the terminally differentiated C2C12 myotubes used in this study, we relied on previous literature to develop our own dose tolerance experiments. For general cell culture studies, across multiple cell lines, a systematic review reported that CBD negatively affects cell viability at doses >2 μM, inducing apoptosis at doses >10 μM. In muscle‐specific studies, Langer, Avey, and Baar (Langer et al., 2022) used 5 μM as the upper limit in their dose–response experiments on skeletal muscle myotubes, but also performed a subset of experiments with 10 μM without reporting any negative effects. Taken together, we used these previous data as a reference and investigated 1, 2.5, 5, and 10 μM of CBD and sCBD. We utilized a metabolic activity assay and propidium iodide exclusion assay to determine cytotoxicity of the CBD and sCBD.

#### 2.3.1 3‐(4,5‐dimethylthiazol‐2‐yl)‐2,5‐diphenyltetrazolium bromide (MTT) metabolic activity assay

NAD(P)H‐dependent cellular oxidoreductase enzymes may reflect the number of viable cells present in cell culture models (Stockert et al., 2018). These enzymes can reduce the tetrazolium dye MTT, to its insoluble formazan, which has a purple color. The amount of formazan produced is directly proportional to the number of living cells present in the culture, however, decreases in cellular metabolic activity may occur before cell death and as such, the MTT assay was used in parallel to the propidium iodide assay describe in the following section to determine the dose‐responsiveness of C2C12s to varying CBD concentrations.

C2C12 myoblasts were seeded at 4 × 10^4^ cells⋅mL^−1^ in pregelatinised 12‐well plates in GM and cultured to 80% confluence. Thereafter, cells were induced to terminally differentiate as described under the cell culture section above. After 8 days of differentiation, existing DM was removed from monolayers. Following three PBS washes, monolayers were treated with either DM containing DMSO (CON) or DM + CBD or sCBD at doses from 1 to 10 μM (Figure 2). Following 24 h treatment, MTT was added to each well at 10% of total well volume and incubated for 180 min. Thereafter, existing media was removed from monolayers before another, short incubation of 6 min, which was completed with plate lids removed. Five hundred microlitres of DMSO was then added to each well, and plates were agitated on a plate rocker at 120 rpm for 2 min to ensure all cells had detached from the plate. Plates were then positioned into a Spark multimode microplate reader (Tecan, Mannedorf, Switzerland) and measured at a wavelength of 570 nm to measure the change in absorbance.

#### 2.3.2 Cell viability assay (Propidium iodide exclusion)

In a separate experiment, myoblasts were seeded at 7 × 10^4^ cells⋅mL^−1^ in pregelatinised 6‐well plates in GM. Plates were incubated for 24 h and, once ~80% cell confluence was reached GM was removed and cell monolayers were washed twice with PBS. Monolayers were then treated with DM and incubated at 37°C degrees, 5% CO_2_ for a further 48 h. Existing media was then removed, and monolayers were washed once with PBS, and fresh DM was introduced to monolayers. Monolayers were topped‐up every 48 h with DM corresponding to 10% of well concentration (100 μL) for 5 days. On day 8 of differentiation, existing media was removed from monolayers, which were subsequently treated with either differentiation media containing vehicle solution DMSO (CON) or at various concentrations of DM + CBD or sCBD from 1 to 10 μM (Figure 2). Following 24 h treatment, existing media was pipetted into 1.5 mL Eppendorf tubes and monolayers were washed twice with prewarmed PBS. Thereafter, trypsin–EDTA 0.025% was added to each well and plates were incubated for 5 min (37°C degrees, 5% CO_2_). The removed media was then placed back onto its corresponding well to allow serum to neutralize the activity of trypsin. This solution was then pipetted into a 1.5 mL Eppendorf and centrifuged for 5 min at 300 × *g*. Following centrifugation, the existing media was aspirated from each Eppendorf, leaving a small pellet of cells. Two hundred microlitres of fresh DM was then added to each Eppendorf, and cell pellets were fully resuspended by pipetting to create a solution of cells and DM. Propidium Iodide (5 mg·mL^−1^ in ddH_2_O) was then added to each Eppendorf at a concentration of 1:100 and these were vortexed for 20 s before a 5‐min incubation (37°C degrees, 5% CO_2_). PI fluorescence was then analyzed via flow cytometry using the FL‐3 channel on a BD Accuri™ C6 Plus Flow Cytometer (BD biosciences, Berkshire, UK). First, negative and positive controls were established whereby PI‐free cells were analyzed for background fluorescence alongside cells treated with 50% *v/v* H_2_O_2_. PI‐free cells had no increase in fluorescence, whilst H_2_O_2_ increased fluorescence (Data not shown). The excitation/emission maximum of the dye is typically 493/636; upon binding to DNA, excitation/emission maxima increased to 535/617 nm.

### 2.4 Next generation RNA sequencing

#### 2.4.1 Cell treatments

After determining the tolerability of C2C12 myotubes to varying doses of CBD and sCBD, we found that 10 μM was the upper limit tolerated by both treatments in MTT and PI experiments (see Figure 2). To determine the transcriptional response to 10 μM CBD and sCBD, we terminally differentiated C2C12 myotubes as described above. On day 8 of differentiation, the differentiation medium (DM) was removed and replaced with DM containing either vehicle (DMSO), CBD (10 μM), or sCBD (10 μM). Myotubes were then cultured in the respective treatments for 24 h before being harvested for RNA extraction. Duplicate wells (technical replicates) were used for each treatment, and the experiment was repeated three times (experimental replicates), generating *n* = 6 samples per treatment.

#### 2.4.2 RNA isolation

Following a 24 h treatment with CBD/sCBD/vehicle control, terminally differentiated C2C12 monolayers were lysed with buffer RLT from the Qiagen RNeasy kit (Qiagen Ltd). RNA was then extracted from cell lysates using the RNeasy kit with proteinase K digestion, as per the manufacturer's guidelines. Eluted RNA was stored at −80°C until required for determination of total RNA and library processing.

#### 2.4.3 Determination of RNA quantity and quality

Total RNA was quantified using a Nanodrop 8000 and RNA quality was assessed using an Agilent® Bioanalyser (average RIN score = 7, 260/280 = 2.1, 260/230 = 1.6). RNA samples were then diluted to 20 ng·μL^−1^ using RNase‐free water.

#### 2.4.4 RNA library preparation and sequencing

Libraries were constructed from 100 ng of total RNA with Poly‐A tail enrichment of mRNA using NEBNext® Ultra™ II RNA Library Prep Kit for Illumina® (Cat. ID. E7770L. New England Biolabs UK. Hitchin, Herts) with Agencourt AMPureXP Sample Purification Beads (Cat. ID. A63880. Beckman Coulter. Wycombe, UK) as per manufacturers guidelines, by Bart's and the London Genome Centre at Queen Mary, University of London. The resultant‐barcoded libraries were sequenced on an Illumina NextSeq 2000 using 2 × 50 bp paired‐end sequencing. An average of 22 million paired‐end reads was achieved per sample (details in Table S1).

### 2.5 Statistical analysis

#### 2.5.1 Dose tolerability

All statistical analysis and figures regarding dose tolerability experiments were conducted using GraphPad Prism™ for Macintosh (Version 9.3.1). All data were normally distributed, and as such were assessed with a one‐way ANOVA. Significance was assumed if α reached ≤0.05. All data are presented as mean (95% confidence intervals [CIs]), ± standard deviation (SD).

#### 2.5.2 RNA sequencing

FastQ files were imported to Partek® Flow® Genomic Analysis Software Partek Inc. (Missouri, USA) for pipeline processing. Pre‐alignment QA/QC was performed on all reads prior to read trimming. STAR alignment 4.4.1d was used to align trimmed reads to the Mus musculus genome. Aligned reads were then quantified to the Ensembl transcriptome annotation model associated with Mus musculus mm39 genome. Post alignment QC reports are provided in Table S1. Filtered raw counts were used for normalization and differential analysis with DESeq2 (Love et al., 2014) through Partek® Flow®. Gene transcripts were considered significantly different between groups when the FDR <0.05 and fold change >1. Volcano plots and annotation charts were generated in R studio (version 2023.06.1 + 524), Principal Component Analysis (PCA) plots were generated in Partek® Flow® and bar charts were constructed in GraphPad Prism version 10.0.0 for Windows (GraphPad Software, Boston, Massachusetts USA).

## 3 BIOINFORMATICS

Hypergeometric Optimization of Motif EnRichment—HOMER (v4.11) motif analysis was performed on DEGs commonly up/downregulated by treatment with CBD or sCBD, to identify the enrichment of known motifs (6–12 bp long) in the gene body and up to 2 kb upstream of the transcription start site (Heinz et al., 2010). The sequence logo's in Table 1 are produced by the HOMER v4.11 tool. Venn Diagram Analysis was performed using the VIB/UGent Venn online tool, http://bioinformatics.psb.ugent.be/webtools/Venn/.

**TABLE 1:** Top 5 conserved putative upstream regulators of CBD and sCBD transcriptional responses.

	% of targets	Log *p*‐value	TF (family)
**Motif (upregulated genes)**			
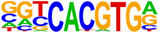	36.60	−1.93E+01	USF1 (bHLH)
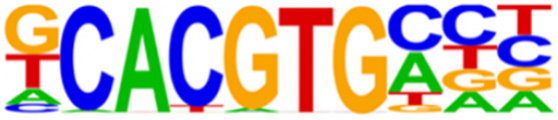	26.52	−1.38E+01	bHLHE40 (bHLH)
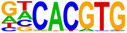	39.36	−1.28E+01	CLOCK (bHLH)
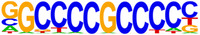	49.17	−1.25E+01	Sp1 (Zf)
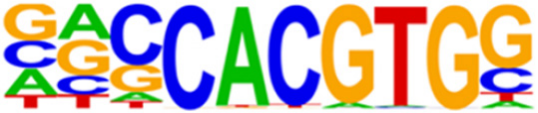	44.34	−1.22E+01	n‐Myc (bHLH)
**Motif (downregulated genes)**			
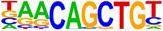	46.21	−1.12E+01	Myf5 (bHLH)
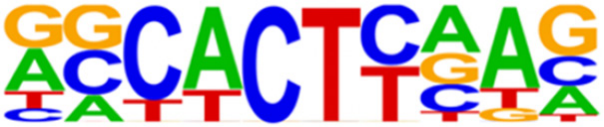	94.95	−1.01E+01	Nkx2.1 (Homeobox)
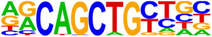	46.99	−9.08E+00	MyoD (bHLH)
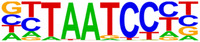	49.51	−8.24E+00	Otx2 (Homeobox)
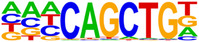	62.14	−7.33E+00	Ap4 (bHLH)

To visualize gene interactions and clusters of proteins that they encode with known physical interactions, STRING (Version 12. string.db.org) was deployed on commonly upregulated and downregulated DEGs. Only physical interactions are shown with the highest degree of confidence (0.900). *K*‐means clustering was used to visualize clusters of genes based on their centroids.

**FIGURE 1:**
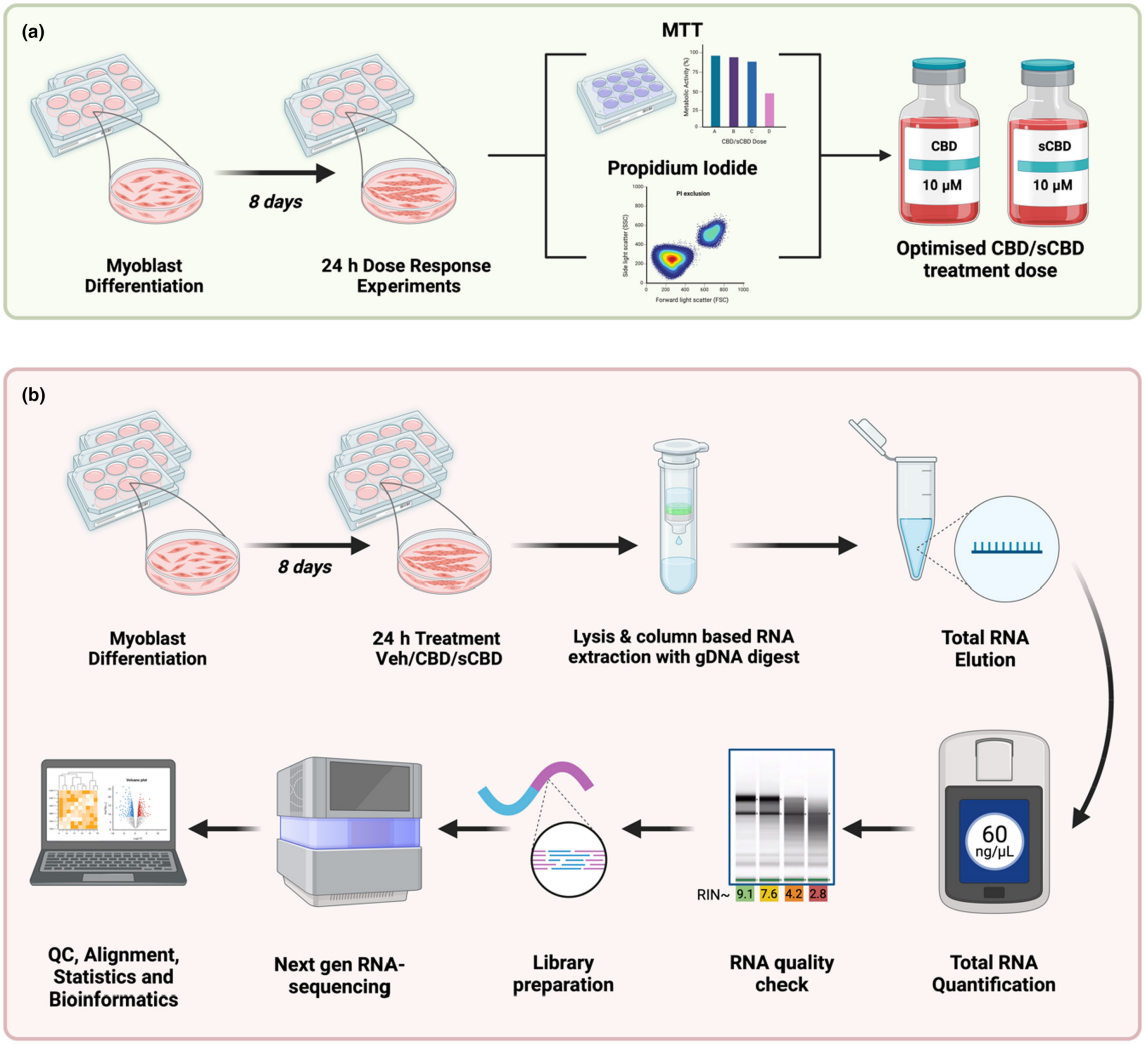
Experimental workflow for (a) the determination of dose tolerability. C2C12 myoblasts were differentiated in low serum media for 8 days before 24 h exposure to CBD, sCBD, or vehicle (DMSO) control. MTT and propidium iodide exclusion assays as well as visual inspection were performed to determine the maximal tolerable dose of CBD and sCBD across a range of concentrations from 0.001 to 10 μM. In workflow (b), differentiated myotubes were exposed to 10 μM CBD, sCBD, and vehicle control for 24 h before being lysed for column‐based RNA extraction. Total RNA quantity and quality were determined prior to library preparation and next generation sequencing. Pre‐alignment QC was performed prior to trimming and STAR alignment, followed by quantification to the annotation model, counts were then normalized by median ratio, prior to DSeq2 statistical analysis. Image created using Biorender.

Experimental workflows are presented schematically in Figure 1.

## 4 RESULTS

### 4.1 Dose tolerability

**FIGURE 2:**
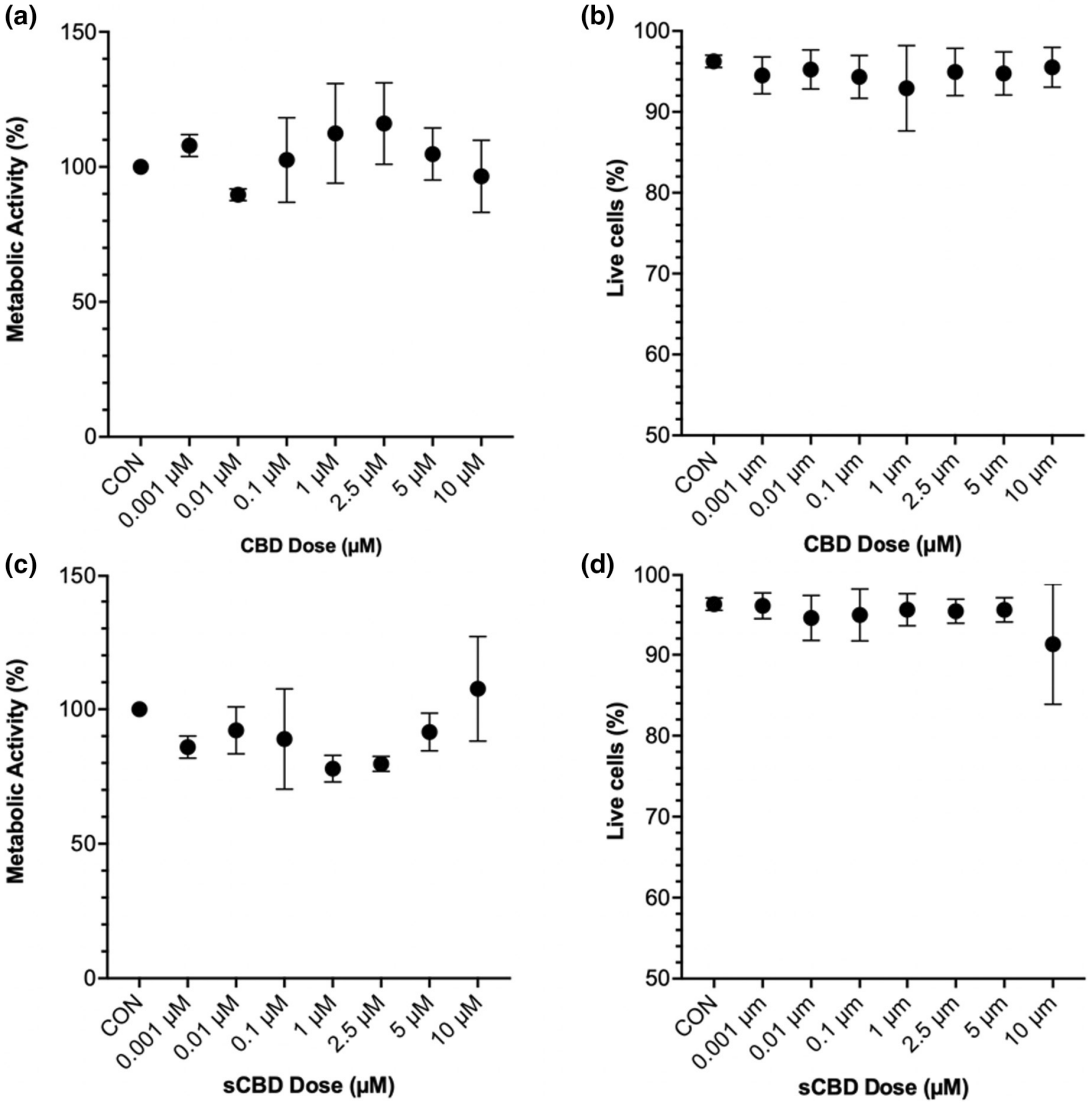
Dose tolerability experiments on terminally differentiated myotubes. Metabolic activity and cell viability were determined by the MTT assay and propidium iodide exclusion in CBD‐treated (a, b) and sCBD‐treated (c, d) C2C12 myotubes over a range of concentrations (0.001–10 μM).

Following 24 h of CBD exposure, metabolic activity and cell health, as determined by MTT assays, showed no significant differences across any of the tested doses (0.001–10 μM) compared to the control (*p* > 0.05; Figure 2a). Additionally, there were no reductions in the percentage of live cells at any CBD dose within the same range (*p* > 0.05; Figure 2b). Similarly, treatment with sCBD for 24 h did not alter metabolic activity at any dose (0.001–10 μM) relative to the control (*p* > 0.05; Figure 2c). Furthermore, the live cell population percentage remained unaffected by any sCBD dose tested (*p* > 0.05; Figure 2d). Based on these observations, along with results from other studies, we selected the maximum 10 μM CBD and sCBD to evaluate their potential effects on the transcriptome of terminally differentiated myotubes.

### 4.2 Transcriptional responses of terminally differentiated C2C12 Myotubes to CBD and sCBD treatment

**FIGURE 3:**
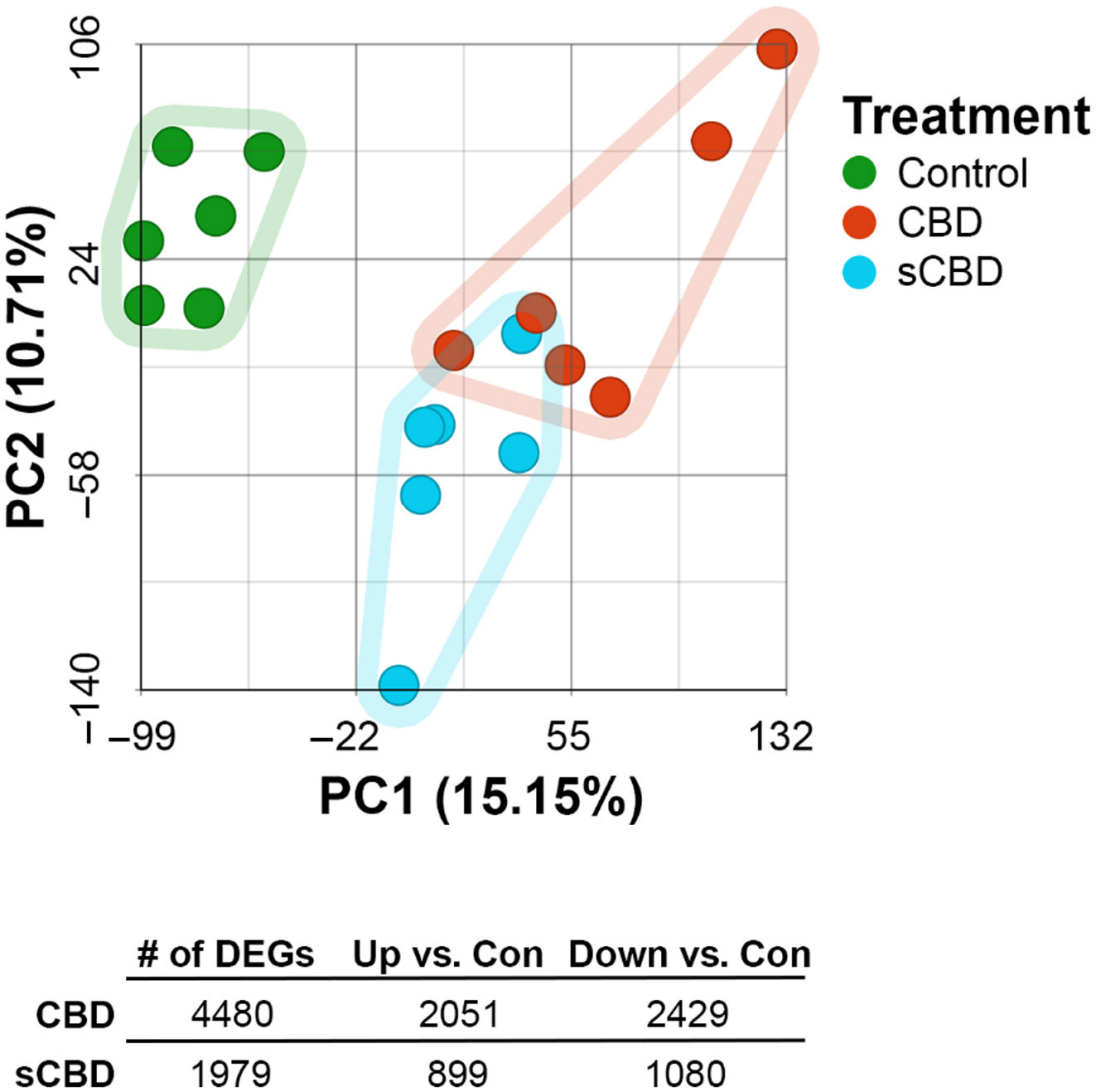
Principal component analysis plot and summary of differentially expressed genes (*p* < 0.001, FDR step‐up <0.05) by condition.

The PCA plot in Figure 3 visualizes the multidimensional relationships amongst the gene expression profiles of CON, CBD, and sCBD‐treated cells. The plot reveals distinct clusters of gene expression profiles for each treatment, but less separation between CBD and sCBD clusters versus CON, indicative of underlying patterns within the data.

**FIGURE 4:**
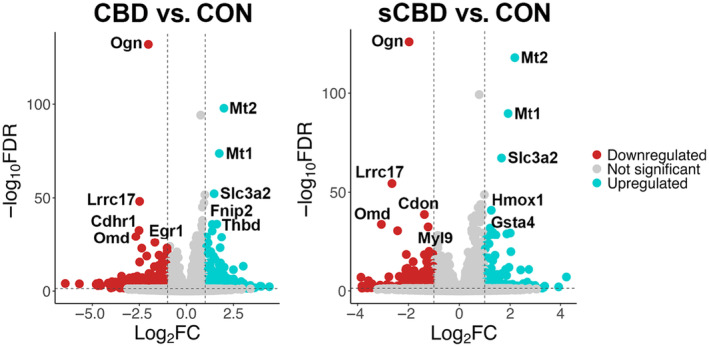
Volcano plot illustrating the relationship between −log_10_FDR step‐up and log_2_ fold‐change for DEGs. Gray data points represent genes that are not statistically significant (−log_10_FDR step up (<0.05) and log_2_FC > −1.1), whilst blue data points are significantly up regulated and red data points are significantly downregulated. Top 5 up and downregulated DEGs are labeled for comparison.

Using an 0.05 FDR cut off, we found that 4480 genes were differentially expressed in CBD versus CON. Of these genes, 2051 were significantly upregulated and 2429 were significantly downregulated. In the sCBD treatment, 1979 genes were differentially expressed. Of these genes, 899 were significantly upregulated and 1080 down were significantly downregulated (Figure 3). Interestingly, of the 5 most up and downregulated genes in CBD and sCBD treated cells, 3 of the most downregulated genes (Osteoglycin; *Ogn*, Leucine‐rich repeat‐containing protein 17; *Lrrc17* and Cadherin‐related family member 1; *Cdhr1*) and 3 of the most upregulated genes (Metallothionein 1 and 2; *Mt1*, *Mt2*, and solute carrier family 3 member 2; *Slc3a2*) were conserved across treatments (Figure 4).

### 4.3 Common upregulated transcriptional responses in CBD and sCBD treated Myotubes

**FIGURE 5:**
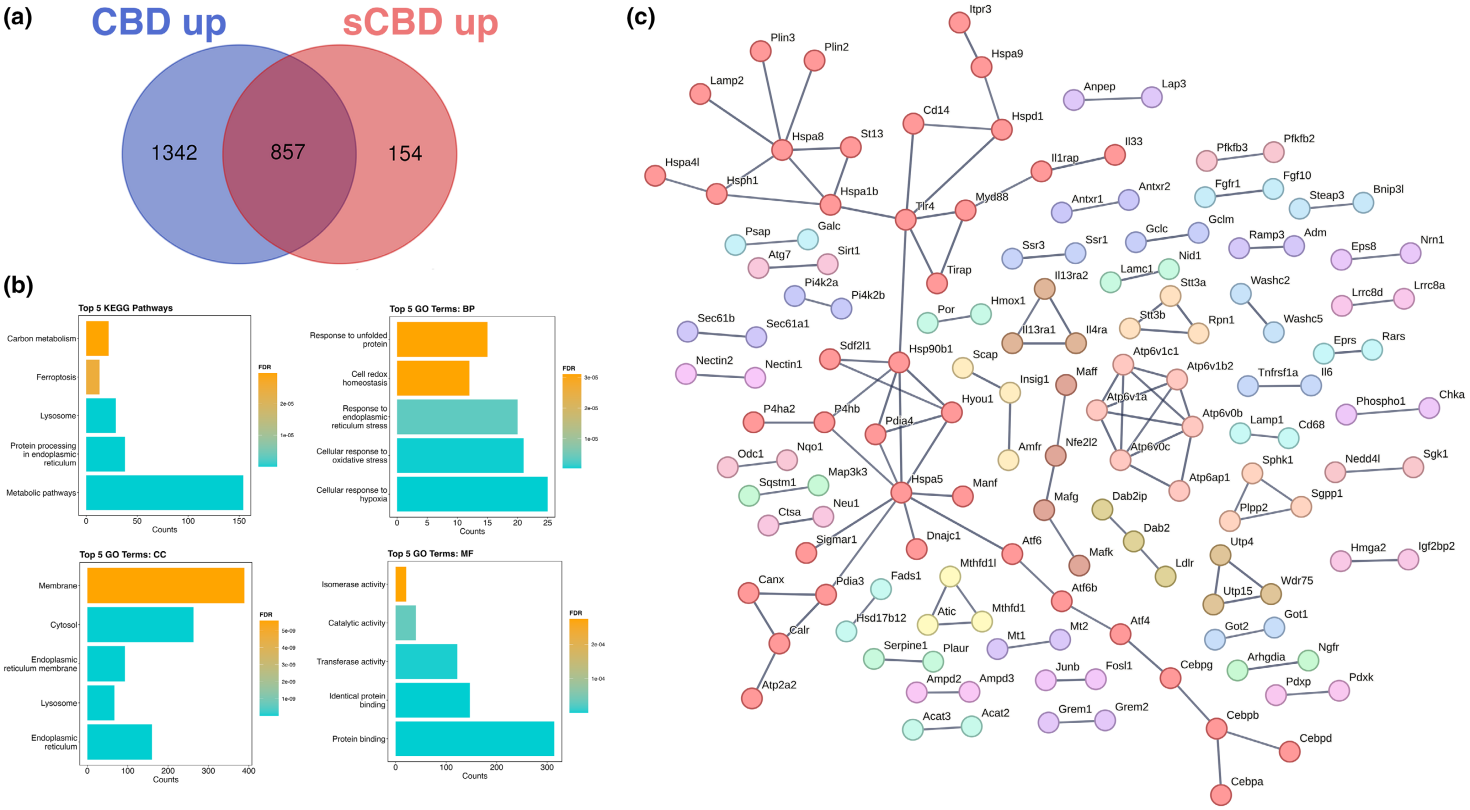
Common upregulated genes; (a) Venn diagram illustrating DEGs uniquely upregulated by CBD and sCBD and common upregulated genes in the overlapping region. (b) The top 5 Enriched KEGG pathways and GO terms for common upregulated genes. BP, biological process; CC, cellular compartment; MF, molecular function. (c) Common up‐regulated genes and their known protein–protein interactions, single nodes are not shown, highest confidence (0.9), clusters are colored individually using *K*‐means clustering.

To identify similarities in the transcriptional responses and gain deeper insight into the genes that may be directly influenced by CBD alone, we performed Venn diagram analysis on significantly up and downregulated DEGs. We identified that 857 genes were commonly upregulated by both CBD and sCBD treatments versus CON and 531 commonly downregulated by both CBD and sCBD versus CON (Figure 5).

To ascertain biological context to our data, we performed network analysis of known protein–protein interactions for proteins encoded by the DEGs in our data set using STRING. By visualizing the data in this way, we can determine what proteins and their associated networks might be most influential to our data, and specifically to CBD since these networks are commonly regulated in both CBD and sCBD treatments. In Figure 5c, we show several networks enriched in our data set for commonly upregulated DEGs. The largest network consists of protein interactions contains several connected chaperones (Heat shock proteins; *Hspa8*, *Hspa1b*, *Hsph1*, *Hspa4l*, *Hsp90b1*, *Hspa5*, *Hspa9*, *Hspd1*) and components of the unfolded protein responses/endoplasmic reticulum (UPR/ER) stress response (Activating transcription factors; *Atf6*, *Atf6b*, *Atf4*) and lysosomal associated membrane protein 2 (*Lamp2*). These results are congruent with our gene ontology analysis of commonly upregulated DEGs, whereby KEGG pathways and GO biological processes show significant enrichment of oxidative stress, UPR/ER stress, and lysosomal pathways (Figure 5b).

### 4.4 Common downregulated transcriptional responses in CBD and sCBD treated Myotubes

**FIGURE 6:**
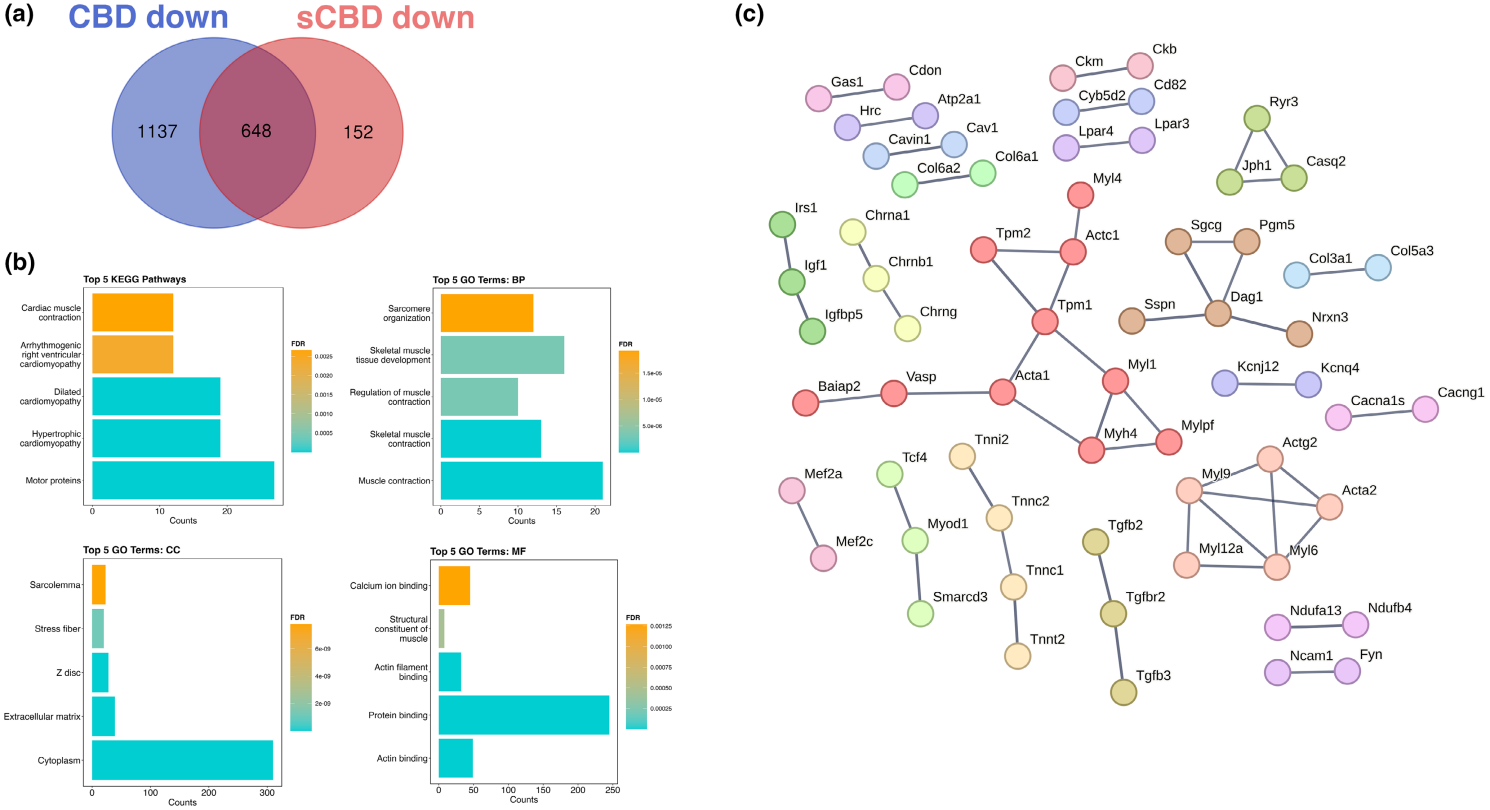
Common downregulated genes; (a) Venn diagram illustrating DEGs uniquely downregulated by CBD and sCBD and common downregulated genes in the overlapping region. (b) The top 5 Enriched KEGG pathways and GO terms for common downregulated genes. BP, biological process; CC, cellular compartment; MF, molecular function. (c) Common downregulated genes and their known protein–protein interactions, single nodes are not shown, highest confidence (0.9), clusters are colored individually using *K*‐means clustering.

We then performed the same set of bioinformatics analyses on the 648 commonly downregulated DEGs. The largest network in our STRING analysis (Figure 6c) consists of motor genes encoding motor proteins, including alpha actin (*Actc1*, *Acta1*, *Actn1*, *Actn4*), myosin light and heavy chains (*Myl1*, *Mylpf*, *Myh4*) and tropomyosin (*Tpm1*, *Tpm2*) as well as cell–cell contact proteins Vinculin (*Vcl*) and Vasodilator stimulated phosphoprotein (*Vasp*). Other clusters also contain motor proteins including troponins (*Tnni2*, *Tnnc2*, *Tnnc1*, *Tnnt2*) and structural collagen proteins (*Col5a1*, *Col1a2*, *Col5a3*, *Col3a1*). Gene ontology analysis of commonly downregulated DEGs revealed pathways associated to muscle contraction, skeletal muscle tissue development, motor proteins, sarcomere organization, and actin binding amongst others (Figure 6b).

### 4.5 Conserved putative upstream regulators of CBD and sCBD transcriptional responses

Finally, to gain understanding of the transcription factors regulating the common transcriptional responses between treatments, we performed hypergeometric optimization of motif enrichment (HOMER) analysis to identify enriched transcription factor DNA binding motifs in the gene body and up to 2 kb upstream of the transcription start site within DEGs.

HOMER analysis of commonly upregulated DEGs identified four basic Helix Loop Helix (bHLH) and one zinc finger transcription factor (TF) in the top 5 TF's (Table 1), with transcriptional activity related to stress responses such as hypoxia and oxidative stress. These features fall in line with our pathway analysis of conserved upregulated genes between (see Figure 5). We identified enrichment of the circadian clock TF bHLHE40, a ubiquitously expressed TF with strong expression in skeletal muscle. bHLHE40 can be induced by various cellular stressors including hypoxia (Miyazaki et al., 2002) and has been identified to protect skeletal muscle from ROS‐induced damage by activating the expression of heme‐oxygenase‐1 (*Hmox1*) (Vercherat et al., 2009), a gene that appears in the top 20 upregulated DEGs for both CBD (2.1‐fold change) and sCBD (2.4‐fold change) in our data set. Similarly, the bHLH TF Myc was enriched and has been linked to cellular responses to stress including oxidative stress and hypoxia (Le et al., 2021). Both treatments also increased expression of *Myc* (~1.4 fold), suggesting a common mechanism of action. Downstream targets of MYC such as MYC downstream regulated gene 1 (*Ndrg1*), also a common upregulated DEG in our dataset, are known to regulate the cellular adaptive response to hypoxia (Park et al., 2022). We identified USF1 and the zinc finger TF SP1 in our HOMER analysis and they too have been shown to regulate the expression of downstream targets in response to hypoxic conditions (Turkoglu & Kockar, 2016). USF1 is thought to mediate transcriptional activity to AMPK activation, driven by an increased in AMP:ATP ratio (Irrcher et al., 2008). Moreover, overexpression of USF1 in skeletal muscle cells leads to increased PGC‐1α promoter activation and similar increases at the mRNA level (Irrcher et al., 2008) indicating the importance of this TF in transcriptional responses to energetic stress.

DNA binding motifs enriched in our conserved downregulated DEGs included myogenic regulatory factors MYF5 and MYOD1 which have extensive roles in myogenesis and the latter, homeostasis of terminally differentiated muscle and the sarcomere. *Myod1* was found to be downregulated in both treatment groups (~1.6‐fold), again suggesting a common mechanism of action. We suggest that this may be responsible for the downregulation of many sarcomeric proteins, as evidenced in Figure 6c. Collectively, these TF's lend support to the pathway analysis presented in Figure 6b, suggestive of a cellular stress response induced by CBD and sCBD exposure, which may be partially responsible for downregulation of Myod1 and a number of genes encoding sarcomeric/contractile components.

A full list of TFs identified by HOMER analysis can be found in Tables S4,S5.

## 5 DISCUSSION

The main findings from our work show that CBD leads to the transcriptional activation and repression of several hundred genes and this is at least partly due to CBD itself rather than the many other compounds found in hemp‐derived, broad‐spectrum CBD. Upon further examination of the DEGs commonly up and downregulated by CBD and sCBD, we observed that many of the upregulated genes are indicative of a “stress” response and commonly downregulated genes allude to muscle remodeling of motor and structural proteins. In line with our hypothesis, we also show that broad‐spectrum CBD treatment induced 1342 unique upregulated DEGs and 1137 unique downregulated DEGs, compared to 154 and 152 unique up‐ and downregulated DEGs in sCBD presumably due to the array of additional compounds present compared to sCBD.

Our data are the first to report transcriptome responses to CBD and sCBD in skeletal muscle, a tissue in which relatively few data exist characterizing the effects of cannabinoids. Mechanistic studies by Langer and colleagues found that CBD exerted no effect on anabolic signaling through mTORC1 or inflammatory signaling through nuclear factor kappa B with CBD doses of 1–5 μM in C2C12 myotubes (Langer et al., 2022). Using a different experimental design, Iannotti et al. established that 1 μM CBD enhanced myogenesis, but 3 μM had an inhibitory effect on myogenin (*Myog*) mRNA expression (Iannotti et al., 2019). In vivo studies provide further complexity. Intraperitoneal injection of 60 mg·kg^−1^ CBD prevented loss of locomotor activity, reduced inflammation, and restored autophagy in mdx mice (Iannotti et al., 2019). In a separate study on healthy rats undergoing eccentric loading of the tibialis anterior, administration of 100 mg·kg^−1^ hemp‐derived CBD decreased pro‐inflammatory signaling without blunting the anabolic response to exercise in rats (Langer et al., 2021). These studies provide valuable targeted insights, but not a clear systems‐level picture of the effects of CBD on muscle.

With regards to dissecting our findings, insights may be found by examining the reported anti‐cancer properties of cannabinoids in various other cell types. One of the mechanisms by which CBD may act to reduce cancer cell proliferation is through apoptosis, with some studies highlighting increased ROS production as a central mechanism. For example, CBD can trigger *Noxa* (*Pmaip1*) mediated apoptosis in colorectal cancer cells in a dose dependent manner (0–8 μM) by generating ROS and inducing excessive endoplasmic reticulum (ER) stress, mainly driven by mitochondrial superoxide anion (Jeong et al., 2019). Notably, the induction of ER stress‐mediated apoptosis was under the action of the activating transcription factors *Atf3* and *Atf4*. Our dataset demonstrates transcriptional activation of *Atf4* and *Atf6*, as well as several molecular chaperones (*Hspa8, Hspa1b, Hsph1, Hspa4l, Hsp90b1, Hspa5, Hspa9, Hspd1*) integral to the ER stress response.

Oxidative stress and ER stress are interconnected cellular processes that can activate the Unfolded Protein Response (UPR). ROS generated during oxidative stress can modulate the activity of UPR signaling pathways by directly oxidizing critical cysteine residues within UPR proteins or by activating redox‐sensitive transcription factors (Malhotra & Kaufman, 2007). Studies on other phytonutrients such as the green tea catechins highlight this link in a physiological setting and provide further insights to the observations we present here. Once considered to be antioxidants, contrary evidence suggests that catechins may in fact act as pro‐oxidants to mediate a mitohormetic responses. For example, Tian et al. (2021) explored the effects of epigallocatechin‐3‐gallate (ECGC) and epicatechin‐3‐gallate (ECG) on *Caenorhabditis elegans* (Tian et al., 2021). The authors show that 2.5 μM of green tea catechins significantly blocks mitochondrial complex I activity and mitochondrial respiration rate leading to a transient rise in ROS production and a drop in ATP production. Further, the authors show that lifespan was extended in *C. elegans* by catechin treatment and posit that this could be mediated through a subsequent upregulation in antioxidant defense enzymes including catalase (*Cat*) and superoxide dismutase (*Sod*) and the energy sensors AMP kinase (*Ampk*) and sirtuin 1 (*Sirt1*). Interestingly, we also show *Sirt1* and *Sod2* both as DEGs significantly upregulated by CBD and sCBD in our dataset. Consistent with these observations and in support of a mitohormetic effect of CBD, ROS‐mediated UPR activation can enhance cellular antioxidant defenses by upregulating the expression of antioxidant enzymes. In our dataset, the antioxidants heme oxygenase‐1 (*Hmox1*), NAD(P)H dehydrogenase quinone 1 (*Nqo1*), glutathione reductase (*Gsr*), peroxiredoxins 5 and 6 (*Prx5*, *Prx6*), thioredoxin reductase 1 (*Txnrd1*) and superoxide dismutase 2 (*Sod2*) are all DEGs commonly upregulated by CBD and sCBD. Moreover, nuclear receptor subfamily 4, group A, member 2 (*Nr4a2*) was also commonly upregulated by CBD and sCBD and serves as a key regulator of antioxidant gene expression (Li et al., 2004; Motohashi & Yamamoto, 2004), via binding of antioxidant response elements and integrating cell signaling pathways to coordinate the expression of antioxidant genes and maintain cellular homeostasis.

During conditions of ER stress, the UPR is activated to restore ER homeostasis by attenuating protein synthesis, enhancing protein folding capacity, and promoting protein degradation through the ubiquitin‐proteasome system and autophagy. Notably, autophagy is required to maintain muscle mass (Masiero et al., 2009) and in a murine model of muscular dystrophy, intraperitoneal injection of CBD restored autophagy as evidenced by an increase in the ratio of LC3‐II to LC3‐I (Iannotti et al., 2019), often used as a marker of autophagic activity (Terman et al., 2007), as well as restoring mRNA transcript levels of the autophagy genes beclin‐1, Atg4, Atg12 and Ulk1. In support of this argument, we also show here that “Lysosome” is in the top 5 enriched KEGG terms and GO cellular compartment terms. The lysosome and autophagy are linked in a degradation pathway, where autophagic cargo is delivered to lysosomes for breakdown and recycling (Mizushima et al., 1998), maintaining cellular health and function. Moreover, the downregulation of contractile and motor proteins shown in Figure 6 could be interpretated as a feedback response to reduced protein translation. Whilst others have shown no attenuation of muscle anabolic signaling in response to CBD (Langer et al., 2021, 2022), we suggest that investigation of individual peptide synthesis rates via deuterium labeling in muscle following CBD exposure may provide better resolution than bulk synthesis rates and anabolic signaling. This in turn, could uncover the possible cellular remodeling of the proteome by CBD.

Calcium mediated ER stress is also an emergent theme in our data with significant up regulation of Calnexin (*Canx*) and Calreticulin (*Calr*) as part of a larger cluster of genes including chaperones and autophagy genes (Figure 4). Others have demonstrated that treatment of C2C12 and human derived myoblasts with CBD increases [Ca^2+^]_i_ via TRPV1 activation (Iannotti et al., 2019), an observation replicated in several other cell types and central to many of the established roles of CBD in pain mediation (Louis‐Gray et al., 2022). TRPV1 activation also promotes ER stress through modulation of endoplasmic reticulum calcium, [Ca^2+^]_ER_, leading to the downstream activation of nuclear targets *Atf4, Atf6* and *Xbp1* (Thomas et al., 2007). Increases in [Ca^2+^]_I_ may be a cause of mitochondrial ROS production, since elevated [Ca^2+^]_i_, due to TRPV1 channel opening, causes an initial increase in [Ca^2+^]_m_ and successive downstream effects, which can be attenuated by TRPV1 antagonists (Zhai et al., 2020). Future studies could explore treatment of myotubes with CBD in the presence or absence of TRPV1 antagonists to decipher the role of calcium in the signaling responses to CBD.

### 5.1 Limitations

A significant issue with cell culture experimentation is the translatability of findings to the in vivo setting, particularly with relevance to the concentration of compound under investigation. Deiana and colleagues report peak plasma CBD concentration of 14 μg·mL^−1^ at 120 min after injection of CBD at 120 mg·kg^−1^ bodyweight in rats, equivalent to about 45 μM (Deiana et al., 2012). Whilst plasma concentration and what the muscle is exposed to in the extracellular fluid may be very different, it is important to recognize that we used a considerably lower concentration of CBD in our in vitro studies (10 μM).

A further limitation is that, whilst C2C12 cells are regarded as a useful model to study basic muscle biology (Sharples & Stewart, 2011), findings must be replicated in human cells. The C2C12 cell line, derived from murine skeletal muscle myoblasts, is an immortalized cell line widely used in muscle research due to its ease of culture and ability to differentiate into myotubes. However, its use introduces species‐specific differences in cell behavior potentially limiting the translatability of findings to humans (Blau et al., 1983; Hernández‐Hernández et al., 2017; Yaffe & Saxel, 1977). Future work could replicate our findings using primary myoblasts obtained from human donors to provide a more translatable dataset. Moreover, it is difficult to achieve 100% differentiation of C2C12 myoblasts into myotubes and thus there are always undifferentiated myoblasts in the cell population studied. Therefore, the signal detected in our RNAseq experiments is derived from a mix of myoblasts and myotubes. This could be overcome in future experimentation using bioengineered muscle constructs that typically allow for longer culture times and promote highly aligned and differentiated myotubes.

Messenger RNA is an important molecular readout of the response to homeostatic perturbations and precedes protein synthesis. It is, however, unclear whether mRNA transcript abundance directly correlates with changes in peptide synthesis (Vogel & Marcotte, 2012) and we cannot rule out a first exposure effect, whereby the initial challenge to cellular homeostasis may be insufficient to repeatedly lead to the changes in the transcriptome that we describe here. To resolve the preliminary insights described here, cellular remodeling following chronic exposure to CBD should be interrogated. Dynamic proteome profiling of muscle exposed to CBD could offer the required resolution to determine whether CBD leads to longer term cell remodeling (Hesketh et al., 2020).

We studied only one form of broad spectrum of CBD in our experiments. Whilst many metabolites of the cannabis plant are shared amongst several cultivars (Wishart et al., 2024), there are metabolites that are not shared across strains and therefore care should be taken in extrapolating our findings to other CBD products.

Finally, we set out to identify the commonalities between broad‐spectrum and sCBD and therefore focussed out efforts on unraveling the biological context of commonly up‐ and downregulated genes. We are aware that there were many genes not commonly regulated by the treatments (i.e., DEGs unique to CBD or sCBD treatment), which we have not examined in the current manuscript as this was not the specific aim of our experiment. We found that 1342 and 154 DEGs were upregulated by CBD and sCBD, respectively. Whereas 1137 v 152 DEGs were downregulated by CBD and sCBD, respectively. As described earlier, broad‐spectrum CBD contains hundreds of other compounds (Wishart et al., 2024) and whilst we did not measure the composition of our broad‐spectrum treatment, this could explain the relatively large number of DEGs up and downregulated in CBD treatment compared with sCBD. As our data are publicly available, this provides a further avenue of investigation for future research.

## 6 CONCLUSION

The transcriptional data presented here provide evidence that CBD may induce a mild cellular stress that activates several pathways associated with altered redox balance, calcium homeostasis, unfolded protein response, and endoplasmic reticulum stress. Based on our work and previous work of others, we hypothesize that CBD can indeed interact with muscle to improve indices of cellular health. We suggest that future work should assess mitochondrial complex activity, mitochondrial metabolite production including ATP production, and ROS production following treatment with CBD to test the hypothesis that cannabinoids may act as mitohormetic compounds capable of improving cellular health.

## FUNDING INFORMATION

This work was supported by Naturecan Ltd who provided funding for for a PhD studentship.

## CONFLICT OF INTEREST STATEMENT

GLC received funding for a PhD studentship (for SG) from Naturecan Ltd., who manufacturer and sell CBD products. Naturecan Ltd. provided the broad‐spectrum CBD used in this study and Pureis Ltd. provided the synthetic CBD as a gift for the research in this study. Neither company were involved in the design, interpretation or writing of this manuscript.

## ETHICS STATEMENT

The experiments within this manuscript were performed exclusively on the C2C12 cell line and ethical approval was not necessary.

## Supporting information


Data S1.

## Data Availability

RNA‐seq data is available in File S1 (normalized counts and differential expression analysis) and raw data can be found at the GEO accession GSE272113. Further data will be made available by the corresponding author upon reasonable request.
