# Supplementary material for: Comparative transcriptomics of broad‐spectrum and synthetic cannabidiol treated C2C12 skeletal myotubes
Source: Physiol Rep. 2024 Sep 17;12(18):e70059. doi: 10.14814/phy2.70059 (PMC11407902; doi:10.14814/phy2.70059)
Supplement: Supplementary file 1 — Data S1. [file PHY2-12-e70059-s001.docx]

## Supplementary Material

**Supplementary Table 1.**

| **Sample Name** | **Total Reads** | **Total Alignments** | **Aligned** | **Unique singleton** | **Unique Paired** | **Non unique paired** | **Non unique singleton** | **Coverage** | **Average Coverage Depth** | **Average Length** | **Average Quality** | **% GC** |
| --- | --- | --- | --- | --- | --- | --- | --- | --- | --- | --- | --- | --- |
| Control 1 | 64,126,659 | 148,292,088 | 96.86% | 0% | 86.29% | 10.57% | 0% | 2.90% | 119.88 | 64.46 | 33.41 | 50.52% |
| Control 2 | 22,713,571 | 52,619,782 | 97.00% | 0% | 86.45% | 10.55% | 0% | 2.34% | 52.69 | 64.46 | 33.42 | 50.59% |
| Control 3 | 23,564,379 | 55,805,953 | 97.51% | 0% | 85.94% | 11.56% | 0% | 2.46% | 53.24 | 64.46 | 33.41 | 50.20% |
| Control 4 | 19,886,054 | 46,270,354 | 97.19% | 0% | 86.44% | 10.75% | 0% | 2.18% | 49.73 | 64.46 | 33.43 | 50.39% |
| Control 5 | 15,546,529 | 36,181,618 | 97.27% | 0% | 86.66% | 10.62% | 0% | 2.11% | 40.19 | 64.46 | 33.43 | 50.73% |
| Control 6 | 24,091,894 | 56,408,542 | 97.02% | 0% | 85.84% | 11.18% | 0% | 2.30% | 57.53 | 64.46 | 33.42 | 50.47% |
| CBD 1 | 21,174,045 | 49,396,243 | 96.97% | 0% | 86.13% | 10.84% | 0% | 2.14% | 54.31 | 64.46 | 33.42 | 50.92% |
| CBD 2 | 18,267,709 | 42,158,999 | 97.62% | 0% | 87.82% | 9.79% | 0% | 2.36% | 42.02 | 64.46 | 33.42 | 50.87% |
| CBD 3 | 25,160,078 | 58,288,151 | 97.69% | 0% | 87.59% | 10.09% | 0% | 2.58% | 53.05 | 64.46 | 33.42 | 50.99% |
| CBD 4 | 21,655,022 | 50,251,580 | 97.51% | 0% | 87.17% | 10.34% | 0% | 2.42% | 48.74 | 64.46 | 33.42 | 50.65% |
| CBD 5 | 25,092,971 | 58,369,189 | 97.55% | 0% | 87.18% | 10.38% | 0% | 2.57% | 53.4 | 64.46 | 33.42 | 51.14% |
| CBD 6 | 16,656,862 | 39,195,786 | 97.24% | 0% | 85.89% | 11.35% | 0% | 2.21% | 41.7 | 64.46 | 33.41 | 50.67% |
| sCBD 1 | 18,354,631 | 42,798,242 | 97.51% | 0% | 86.93% | 10.58% | 0% | 2.32% | 43.32 | 64.46 | 33.43 | 50.36% |
| sCBD 2 | 25,542,692 | 60,510,362 | 97.35% | 0% | 85.70% | 11.65% | 0% | 2.50% | 56.83 | 64.47 | 33.42 | 50.05% |
| sCBD 3 | 21,107,615 | 49,347,107 | 97.28% | 0% | 86.45% | 10.83% | 0% | 2.41% | 48.17 | 64.46 | 33.42 | 50.81% |
| sCBD 4 | 19,061,367 | 44,379,116 | 96.63% | 0% | 85.80% | 10.83% | 0% | 2.25% | 46.24 | 64.46 | 33.45 | 50.93% |
| sCBD 5 | 22,163,696 | 51,559,992 | 97.30% | 0% | 86.76% | 10.54% | 0% | 2.52% | 47.96 | 64.46 | 33.42 | 50.75% |
| sCBD 6 | 20,816,663 | 48,730,997 | 97.35% | 0% | 86.56% | 10.79% | 0% | 2.15% | 53.24 | 64.46 | 33.42 | 50.98% |

**Supplementary Table 2**.

| **Cluster number** | **Cluster colour** | **Gene count** | **Protein name** | **Protein identifier** | **Protein description** |
| --- | --- | --- | --- | --- | --- |
| 1 | Red | 38 | Atf4 | 10090.ENSMUSP00000105234 | Cyclic AMP-dependent transcription factor ATF-4; Transcriptional activator. |
| 1 | Red | 38 | Atf6 | 10090.ENSMUSP00000027974 | Processed cyclic AMP-dependent transcription factor ATF-6 alpha; Transmembrane glycoprotein of the endoplasmic reticulum that functions as a transcription activator and initiates the unfolded protein response (UPR) during endoplasmic reticulum stress. |
| 1 | Red | 38 | Atf6b | 10090.ENSMUSP00000015605 | Processed cyclic AMP-dependent transcription factor ATF-6 beta; Transcriptional factor that acts in the unfolded protein response (UPR) pathway by activating UPR target genes induced during ER stress. |
| 1 | Red | 38 | Atp2a2 | 10090.ENSMUSP00000031423 | Sarcoplasmic/endoplasmic reticulum calcium ATPase 2; This magnesium-dependent enzyme catalyzes the hydrolysis of ATP coupled with the translocation of calcium from the cytosol to the sarcoplasmic reticulum lumen. |
| 1 | Red | 38 | Calr | 10090.ENSMUSP00000003912 | Calreticulin; Calcium-binding chaperone that promotes folding, oligomeric assembly and quality control in the endoplasmic reticulum (ER) via the calreticulin/calnexin cycle. |
| 1 | Red | 38 | Canx | 10090.ENSMUSP00000137440 | Calnexin; Calcium-binding protein that interacts with newly synthesized glycoproteins in the endoplasmic reticulum. |
| 1 | Red | 38 | Cd14 | 10090.ENSMUSP00000056669 | Monocyte differentiation antigen CD14; Coreceptor for bacterial lipopolysaccharide. |
| 1 | Red | 38 | Cebpa | 10090.ENSMUSP00000096129 | CCAAT/enhancer-binding protein alpha; Transcription factor that coordinates proliferation arrest and the differentiation of myeloid progenitors, adipocytes, hepatocytes, and cells of the lung and the placenta. |
| 1 | Red | 38 | Cebpb | 10090.ENSMUSP00000069850 | CCAAT/enhancer-binding protein beta; Important transcription factor regulating the expression of genes involved in immune and inflammatory responses. |
| 1 | Red | 38 | Cebpd | 10090.ENSMUSP00000148145 | CCAAT/enhancer-binding protein delta; Transcription activator that recognizes two different DNA motifs: the CCAAT homology common to many promoters and the enhanced core homology common to many enhancers. |
| 1 | Red | 38 | Cebpg | 10090.ENSMUSP00000118588 | CCAAT/enhancer-binding protein gamma; Transcription factor that binds to the promoter and the enhancer regions of target genes. |
| 1 | Red | 38 | Dnajc1 | 10090.ENSMUSP00000126321 | DnaJ homolog subfamily C member 1; May modulate protein synthesis. |
| 1 | Red | 38 | Hsp90b1 | 10090.ENSMUSP00000020238 | Endoplasmin; Molecular chaperone that functions in the processing and transport of secreted proteins. |
| 1 | Red | 38 | Hspa1b | 10090.ENSMUSP00000133815 | Heat shock 70 kDa protein 1B; Molecular chaperone implicated in a wide variety of cellular processes, including protection of the proteome from stress, folding and transport of newly synthesized polypeptides, activation of proteolysis of misfolded proteins and the formation and dissociation of protein complexes. |
| 1 | Red | 38 | Hspa4l | 10090.ENSMUSP00000145468 | Heat shock 70 kDa protein 4L; Possesses chaperone activity in vitro where it inhibits aggregation of citrate synthase. |
| 1 | Red | 38 | Hspa5 | 10090.ENSMUSP00000028222 | Endoplasmic reticulum chaperone BiP; Endoplasmic reticulum chaperone that plays a key role in protein folding and quality control in the endoplasmic reticulum lumen. |
| 1 | Red | 38 | Hspa8 | 10090.ENSMUSP00000015800 | Heat shock cognate 71 kDa protein; Molecular chaperone implicated in a wide variety of cellular processes, including protection of the proteome from stress, folding and transport of newly synthesized polypeptides, activation of proteolysis of misfolded proteins and the formation and dissociation of protein complexes. |
| 1 | Red | 38 | Hspa9 | 10090.ENSMUSP00000025217 | Stress-70 protein, mitochondrial; Chaperone protein which plays an important role in mitochondrial iron-sulfur cluster (ISC) biogenesis. |
| 1 | Red | 38 | Hspd1 | 10090.ENSMUSP00000027123 | 60 kDa heat shock protein, mitochondrial; Chaperonin implicated in mitochondrial protein import and macromolecular assembly. |
| 1 | Red | 38 | Hsph1 | 10090.ENSMUSP00000144413 | Heat shock protein 105 kDa; Acts as a nucleotide-exchange factor (NEF) for chaperone proteins HSPA1A and HSPA1B, promoting the release of ADP from HSPA1A/B thereby triggering client/substrate protein release. Prevents the aggregation of denatured proteins in cells under severe stress, on which the ATP levels decrease markedly. Inhibits HSPA8/HSC70 ATPase and chaperone activities. |
| 1 | Red | 38 | Hyou1 | 10090.ENSMUSP00000123700 | Hypoxia up-regulated protein 1; Has a pivotal role in cytoprotective cellular mechanisms triggered by oxygen deprivation. |
| 1 | Red | 38 | Il1rap | 10090.ENSMUSP00000093843 | Interleukin-1 receptor accessory protein; Coreceptor for IL1RL2 in the IL-36 signaling system. Coreceptor with IL1R1 in the IL-1 signaling system. |
| 1 | Red | 38 | Il33 | 10090.ENSMUSP00000025724 | Interleukin-33(102-266); Cytokine that binds to and signals through the IL1RL1/ST2 receptor which in turn activates NF-kappa-B and MAPK signaling pathways in target cells. Involved in the maturation of Th2 cells inducing the secretion of T-helper type 2-associated cytokines. Also involved in activation of mast cells, basophils, eosinophils and natural killer cells. Acts as a chemoattractant for Th2 cells, and may function as an 'alarmin', that amplifies immune responses during tissue injury; Belongs to the IL-1 family. Highly divergent. |
| 1 | Red | 38 | Itpr3 | 10090.ENSMUSP00000038150 | Inositol 1,4,5-trisphosphate receptor type 3; Receptor for inositol 1,4,5-trisphosphate, a second messenger that mediates the release of intracellular calcium. |
| 1 | Red | 38 | Lamp2 | 10090.ENSMUSP00000074448 | Lysosome-associated membrane glycoprotein 2; Plays an important role in chaperone-mediated autophagy. Functions by binding target proteins, such as GAPDH and MLLT11, and targeting them for lysosomal degradation. Required for the fusion of autophagosomes with lysosomes during autophagy. |
| 1 | Red | 38 | Manf | 10090.ENSMUSP00000124562 | Mesencephalic astrocyte-derived neurotrophic factor; Selectively promotes the survival of dopaminergic neurons of the ventral mid-brain. |
| 1 | Red | 38 | Myd88 | 10090.ENSMUSP00000035092 | Myeloid differentiation primary response protein MyD88; Adapter protein involved in the Toll-like receptor and IL-1 receptor signaling pathway in the innate immune response. |
| 1 | Red | 38 | P4ha2 | 10090.ENSMUSP00000019050 | Prolyl 4-hydroxylase subunit alpha-2; Catalyzes the post-translational formation of 4- hydroxyproline in -Xaa-Pro-Gly- sequences in collagens and other proteins. |
| 1 | Red | 38 | P4hb | 10090.ENSMUSP00000026122 | Protein disulfide-isomerase; This multifunctional protein catalyzes the formation, breakage and rearrangement of disulfide bonds. |
| 1 | Red | 38 | Pdia3 | 10090.ENSMUSP00000028683 | Protein disulfide-isomerase A3. |
| 1 | Red | 38 | Pdia4 | 10090.ENSMUSP00000076521 | Protein disulfide-isomerase A4. |
| 1 | Red | 38 | Plin2 | 10090.ENSMUSP00000000466 | Perilipin-2; May be involved in development and maintenance of adipose tissue. |
| 1 | Red | 38 | Plin3 | 10090.ENSMUSP00000019726 | Perilipin-3; Required for the transport of mannose 6-phosphate receptors (MPR) from endosomes to the trans-Golgi network; Belongs to the perilipin family. |
| 1 | Red | 38 | Sdf2l1 | 10090.ENSMUSP00000023453 | Stromal cell-derived factor 2-like protein 1. |
| 1 | Red | 38 | Sigmar1 | 10090.ENSMUSP00000056027 | Sigma non-opioid intracellular receptor 1; Functions in lipid transport from the endoplasmic reticulum and is involved in a wide array of cellular functions probably through regulation of the biogenesis of lipid microdomains at the plasma membrane. |
| 1 | Red | 38 | St13 | 10090.ENSMUSP00000130195 | Hsc70-interacting protein; One HIP oligomer binds the ATPase domains of at least two HSC70 molecules dependent on activation of the HSC70 ATPase by HSP40. |
| 1 | Red | 38 | Tirap | 10090.ENSMUSP00000135435 | Toll/interleukin-1 receptor domain-containing adapter protein; Adapter involved in the TLR2 and TLR4 signaling pathways in the innate immune response. Acts via IRAK2 and TRAF-6, leading to the activation of NF-kappa-B, MAPK1, MAPK3 and JNK, and resulting in cytokine secretion and the inflammatory response. Positively regulates the production of TNF-alpha and interleukin-6. |
| 1 | Red | 38 | Tlr4 | 10090.ENSMUSP00000045770 | Toll-like receptor 4; Cooperates with LY96 and CD14 to mediate the innate immune response to bacterial lipopolysaccharide (LPS). Acts via MYD88, TIRAP and TRAF6, leading to NF-kappa-B activation, cytokine secretion and the inflammatory response. Also involved in LPS- independent inflammatory responses triggered by free fatty acids, such as palmitate. |
| 2 | Salmon | 6 | Atp6ap1 | 10090.ENSMUSP00000019231 | V-type proton ATPase subunit S1; Accessory subunit of the proton-transporting vacuolar (V)- ATPase protein pump, which is required for luminal acidification of secretory vesicles. Guides the V-type ATPase into specialized subcellular compartments, such as neuroendocrine regulated secretory vesicles or the ruffled border of the osteoclast, thereby regulating its activity. Involved in membrane trafficking and Ca^2+^-dependent membrane fusion. |
| 2 | Salmon | 6 | Atp6v0b | 10090.ENSMUSP00000047682 | V-type proton ATPase 21 kDa proteolipid subunit; Proton-conducting pore forming subunit of the membrane integral V0 complex of vacuolar ATPase. V-ATPase is responsible for acidifying a variety of intracellular compartments in eukaryotic cells. |
| 2 | Salmon | 6 | Atp6v0c | 10090.ENSMUSP00000024932 | V-type proton ATPase 16 kDa proteolipid subunit; Proton-conducting pore forming subunit of the membrane integral V0 complex of vacuolar ATPase. V-ATPase is responsible for acidifying a variety of intracellular compartments in eukaryotic cells. |
| 2 | Salmon | 6 | Atp6v1a | 10090.ENSMUSP00000110314 | V-type proton ATPase catalytic subunit A; Catalytic subunit of the peripheral V1 complex of vacuolar ATPase. V-ATPase vacuolar ATPase is responsible for acidifying a variety of intracellular compartments in eukaryotic cells. In aerobic conditions, involved in intracellular iron homeostasis, thus triggering the activity of Fe(2+) prolyl hydroxylase (PHD) enzymes, and leading to HIF1A hydroxylation and subsequent proteasomal degradation. |
| 2 | Salmon | 6 | Atp6v1b2 | 10090.ENSMUSP00000006435 | V-type proton ATPase subunit B, brain isoform; Non-catalytic subunit of the peripheral V1 complex of vacuolar ATPase. V-ATPase is responsible for acidifying a variety of intracellular compartments in eukaryotic cells. |
| 2 | Salmon | 6 | Atp6v1c1 | 10090.ENSMUSP00000022904 | V-type proton ATPase subunit C 1; Subunit of the peripheral V1 complex of vacuolar ATPase. Subunit C is necessary for the assembly of the catalytic sector of the enzyme and is likely to have a specific function in its catalytic activity. V-ATPase is responsible for acidifying a variety of intracellular compartments in eukaryotic cells. |
| 3 | Fire Brick | 4 | Maff | 10090.ENSMUSP00000094076 | Transcription factor MafF; Since they lack a putative transactivation domain, the small Mafs behave as transcriptional repressors when they dimerize among themselves. However, they seem to serve as transcriptional activators by dimerizing with other (usually larger) basic-zipper proteins, such as NFE2L1/NRF1, and recruiting them to specific DNA-binding sites. Interacts with the upstream promoter region of the oxytocin receptor gene. May be a transcriptional enhancer in the up-regulation of the oxytocin receptor gene at parturition. |
| 3 | Fire Brick | 4 | Mafg | 10090.ENSMUSP00000053899 | Transcription factor MafG; Since they lack a putative transactivation domain, the small Mafs behave as transcriptional repressors when they dimerize among themselves. However, they seem to serve as transcriptional activators by dimerizing with other (usually larger) basic-zipper proteins, such as NFE2, NFE2L1 and NFE2L2, and recruiting them to specific DNA-binding sites. |
| 3 | Fire Brick | 4 | Mafk | 10090.ENSMUSP00000106460 | Transcription factor MafK; Since they lack a putative transactivation domain, the small Mafs behave as transcriptional repressors when they dimerize among themselves (By similarity). However, they act as transcriptional activators by dimerizing with other (usually larger) basic-zipper proteins, such as NFE2, NFE2L1/NRF1, NFE2L2/NRF2 and NFE2L3/NRF3, and recruiting them to specific DNA-binding sites. Small Maf proteins heterodimerize with Fos and may act as competitive repressors of the NF-E2 transcription factor. |
| 3 | Fire Brick | 4 | Nfe2l2 | 10090.ENSMUSP00000099733 | Nuclear factor erythroid 2-related factor 2; Transcription factor that plays a key role in the response to oxidative stress: binds to antioxidant response (ARE) elements present in the promoter region of many cytoprotective genes, such as phase 2 detoxifying enzymes, and promotes their expression, thereby neutralizing reactive electrophiles. |
| 4 | Sandy Brown | 3 | Plpp2 | 10090.ENSMUSP00000069670 | Phospholipid phosphatase 2; Magnesium-independent phospholipid phosphatase that catalyzes the dephosphorylation of a variety of glycerolipid and sphingolipid phosphate esters including phosphatidate/PA, lysophosphatidate/LPA, sphingosine 1-phosphate/S1P and ceramide 1-phosphate/C1P. |
| 4 | Sandy Brown | 3 | Sgpp1 | 10090.ENSMUSP00000021450 | Sphingosine-1-phosphate phosphatase 1; Specifically dephosphorylates sphingosine 1-phosphate (S1P), dihydro-S1P, and phyto-S1P. Does not act on ceramide 1-phosphate, lysophosphatidic acid or phosphatidic acid. |
| 4 | Sandy Brown | 3 | Sphk1 | 10090.ENSMUSP00000131010 | Sphingosine kinase 1; Catalyzes the phosphorylation of sphingosine to form sphingosine 1-phosphate (SPP), a lipid mediator with both intra- and extracellular functions. |
| 5 | Saddle Brown | 3 | Il13ra1 | 10090.ENSMUSP00000033418 | Interleukin-13 receptor subunit alpha-1; Binds with low affinity to interleukin-13 (IL13). Together with IL4RA can form a functional receptor for IL13. |
| 5 | Saddle Brown | 3 | Il13ra2 | 10090.ENSMUSP00000033646 | Interleukin-13 receptor subunit alpha-2; Binds as a monomer with high affinity to interleukin-13 (IL13); Belongs to the type I cytokine receptor family. Type 5 subfamily. |
| 5 | Saddle Brown | 3 | Il4ra | 10090.ENSMUSP00000033004 | Soluble interleukin-4 receptor subunit alpha; Receptor for both interleukin 4 and interleukin 13. |
| 6 | Sandy Brown 2 | 3 | Rpn1 | 10090.ENSMUSP00000032143 | Dolichyl-diphosphooligosaccharide--protein glycosyltransferase subunit 1; Subunit of the oligosaccharyl transferase (OST) complex that catalyzes the initial transfer of a defined glycan (Glc(3)Man(9)GlcNAc(2) in eukaryotes) from the lipid carrier dolichol- pyrophosphate to an asparagine residue within an Asn-X-Ser/Thr consensus motif in nascent polypeptide chains, the first step in protein N-glycosylation. |
| 6 | Sandy Brown 2 | 3 | Stt3a | 10090.ENSMUSP00000113116 | Dolichyl-diphosphooligosaccharide--protein glycosyltransferase subunit STT3A; Catalytic subunit of the oligosaccharyl transferase (OST) complex that catalyzes the initial transfer of a defined glycan (Glc(3)Man(9)GlcNAc(2) in eukaryotes) from the lipid carrier dolichol- pyrophosphate to an asparagine residue within an Asn-X-Ser/Thr consensus motif in nascent polypeptide chains, the first step in protein N-glycosylation. |
| 6 | Sandy Brown 2 | 3 | Stt3b | 10090.ENSMUSP00000035010 | Dolichyl-diphosphooligosaccharide--protein glycosyltransferase subunit STT3B; Catalytic subunit of the oligosaccharyl transferase (OST) complex that catalyzes the initial transfer of a defined glycan (Glc(3)Man(9)GlcNAc(2) in eukaryotes) from the lipid carrier dolichol- pyrophosphate to an asparagine residue within an Asn-X-Ser/Thr consensus motif in nascent polypeptide chains, the first step in protein N-glycosylation. |
| 7 | Dark Golden Rod | 3 | Utp15 | 10090.ENSMUSP00000048204 | U3 small nucleolar RNA-associated protein 15 homolog; Ribosome biogenesis factor. Involved in nucleolar processing of pre-18S ribosomal RNA. Required for optimal pre-ribosomal RNA transcription by RNA polymerase I. |
| 7 | Dark Golden Rod | 3 | Utp4 | 10090.ENSMUSP00000048377 | U3 small nucleolar RNA-associated protein 4 homolog; Ribosome biogenesis factor. Involved in nucleolar processing of pre-18S ribosomal RNA. Involved in small subunit (SSU) pre-rRNA processing at sites A', A0, 1 and 2b. Required for optimal pre- ribosomal RNA transcription by RNA polymerase. May be a transcriptional regulator. Acts as a positive regulator of HIVEP1. |
| 7 | Dark Golden Rod | 3 | Wdr75 | 10090.ENSMUSP00000027139 | WD repeat-containing protein 75; Ribosome biogenesis factor. Involved in nucleolar processing of pre-18S ribosomal RNA. Required for optimal pre-ribosomal RNA transcription by RNA polymerase I. |
| 8 | Sandy Brown 3 | 3 | Amfr | 10090.ENSMUSP00000052258 | E3 ubiquitin-protein ligase AMFR; E3 ubiquitin-protein ligase that mediates the polyubiquitination of a number of proteins such as CD3D, CYP3A4, CFTR and APOB for proteasomal degradation. |
| 8 | Sandy Brown 3 | 3 | Insig1 | 10090.ENSMUSP00000061877 | Insulin-induced gene 1 protein; Mediates feedback control of cholesterol synthesis by controlling SCAP and HMGCR. Functions by blocking the processing of sterol regulatory element-binding proteins (SREBPs). |
| 8 | Sandy Brown 3 | 3 | Scap | 10090.ENSMUSP00000095953 | Sterol regulatory element-binding protein cleavage-activating protein; Escort protein required for cholesterol as well as lipid homeostasis. Regulates export of the SCAP/SREBF complex from the ER upon low cholesterol. |
| 9 | Brown | 3 | Dab2 | 10090.ENSMUSP00000079689 | Disabled homolog 2; Adapter protein that functions as clathrin-associated sorting protein (CLASP) required for clathrin-mediated endocytosis of selected cargo proteins. |
| 9 | Brown | 3 | Dab2ip | 10090.ENSMUSP00000088532 | Disabled homolog 2-interacting protein; Functions as a scaffold protein implicated in the regulation of a large spectrum of both general and specialized signaling pathways. Involved in several processes such as innate immune response, inflammation and cell growth inhibition, apoptosis, cell survival, angiogenesis, cell migration and maturation. |
| 9 | Brown | 3 | Ldlr | 10090.ENSMUSP00000034713 | Low-density lipoprotein receptor; Binds LDL, the major cholesterol-carrying lipoprotein of plasma, and transports it into cells by endocytosis. In order to be internalized, the receptor-ligand complexes must first cluster into clathrin-coated pits; Belongs to the LDLR family. |
| 10 | Yellow | 3 | Atic | 10090.ENSMUSP00000027384 | Phosphoribosylaminoimidazolecarboxamide formyltransferase; Bifunctional enzyme that catalyzes 2 steps in purine biosynthesis; Belongs to the PurH family. |
| 10 | Yellow | 3 | Mthfd1 | 10090.ENSMUSP00000021443 | C-1-tetrahydrofolate synthase, cytoplasmic, N-terminally processed; In the N-terminal section; belongs to the tetrahydrofolate dehydrogenase/cyclohydrolase family. |
| 10 | Yellow | 3 | Mthfd1l | 10090.ENSMUSP00000112897 | Monofunctional C1-tetrahydrofolate synthase, mitochondrial; May provide the missing metabolic reaction required to link the mitochondria and the cytoplasm in the mammalian model of one-carbon folate metabolism in embryonic an transformed cells complementing thus the enzymatic activities of MTHFD2; In the N-terminal section; belongs to the tetrahydrofolate dehydrogenase/cyclohydrolase family. |
| 11 | Green | 2 | Arhgdia | 10090.ENSMUSP00000063714 | Rho GDP-dissociation inhibitor 1; Controls Rho proteins homeostasis. Regulates the GDP/GTP exchange reaction of the Rho proteins by inhibiting the dissociation of GDP from them, and the subsequent binding of GTP to them. |
| 11 | Green | 2 | Ngfr | 10090.ENSMUSP00000000122 | Tumor necrosis factor receptor superfamily member 16; Low affinity neurotrophin receptor which can bind to mature NGF, BDNF, NTF3, and NTF4. |
| 12 | Light Green | 2 | Map3k3 | 10090.ENSMUSP00000002044 | Mitogen-activated protein kinase kinase kinase 3; Component of a protein kinase signal transduction cascade. Mediates activation of the NF-kappa-B, AP1 and DDIT3 transcriptional regulators. |
| 12 | Light Green | 2 | Sqstm1 | 10090.ENSMUSP00000099835 | Sequestosome-1; Autophagy receptor required for selective macroautophagy (aggrephagy). Functions as a bridge between polyubiquitinated cargo and autophagosomes. Interacts directly with both the cargo to become degraded and an autophagy modifier of the MAP1 LC3 family. |
| 13 | Medium Aqua Marine | 2 | Plaur | 10090.ENSMUSP00000002284 | Urokinase plasminogen activator surface receptor; Acts as a receptor for urokinase plasminogen activator. Plays a role in localizing and promoting plasmin formation. Mediates the proteolysis-independent signal transduction activation effects of U-PA. |
| 13 | Medium Aqua Marine | 2 | Serpine1 | 10090.ENSMUSP00000039586 | Plasminogen activator inhibitor 1; Serine protease inhibitor. Inhibits TMPRSS7. Is a primary inhibitor of tissue-type plasminogen activator (PLAT) and urokinase- type plasminogen activator (PLAU). |
| 14 | Aquamarine 4 | 2 | Lamc1 | 10090.ENSMUSP00000027752 | Laminin subunit gamma-1; Binding to cells via a high affinity receptor, laminin is thought to mediate the attachment, migration and organization of cells into tissues during embryonic development by interacting with other extracellular matrix components. |
| 14 | Aquamarine 4 | 2 | Nid1 | 10090.ENSMUSP00000005532 | Nidogen-1; Sulfated glycoprotein widely distributed in basement membranes and tightly associated with laminin. Also binds to collagen IV and perlecan. It probably has a role in cell-extracellular matrix interactions. |
| 15 | Aquamarine 2 | 2 | Hmox1 | 10090.ENSMUSP00000005548 | Heme oxygenase 1; Heme oxygenase cleaves the heme ring at the alpha methene bridge to form biliverdin. Biliverdin is subsequently converted to bilirubin by biliverdin reductase. Under physiological conditions, the activity of heme oxygenase is highest in the spleen, where senescent erythrocytes are sequestrated and destroyed. Exhibits cytoprotective effects since excess of free heme sensitizes cells to undergo apoptosis. |
| 15 | Aquamarine 2 | 2 | Por | 10090.ENSMUSP00000005651 | NADPH--cytochrome P450 reductase; This enzyme is required for electron transfer from NADP to cytochrome P450 in microsomes. It can also provide electron transfer to heme oxygenase and cytochrome B5; In the N-terminal section; belongs to the flavodoxin family. |
| 16 | Cyan | 2 | Acat2 | 10090.ENSMUSP00000007005 | Acetyl-CoA acetyltransferase, cytosolic; Involved in the biosynthetic pathway of cholesterol. |
| 16 | Cyan | 2 | Acat3 | 10090.ENSMUSP00000125454 | Acetyl-Coenzyme A acetyltransferase 3; Belongs to the thiolase-like superfamily. Thiolase family. |
| 17 | Aquamarine | 2 | Fads1 | 10090.ENSMUSP00000010807 | Acyl-CoA (8-3)-desaturase; Acts as a front-end fatty acyl-coenzyme A (CoA) desaturase that introduces a cis double bond at carbon 5 located between a preexisting double bond and the carboxyl end of the fatty acyl chain. |
| 17 | Aquamarine | 2 | Hsd17b12 | 10090.ENSMUSP00000028619 | Very-long-chain 3-oxoacyl-CoA reductase; Catalyzes the second of the four reactions of the long-chain fatty acids elongation cycle. |
| 18 | Aquamarine 3 | 2 | Cd68 | 10090.ENSMUSP00000018918 | Macrosialin; Could play a role in phagocytic activities of tissue macrophages, both in intracellular lysosomal metabolism and extracellular cell-cell and cell-pathogen interactions. |
| 18 | Aquamarine 3 | 2 | Lamp1 | 10090.ENSMUSP00000033824 | Lysosome-associated membrane glycoprotein 1; Presents carbohydrate ligands to selectins. Also implicated in tumor cell metastasis. |
| 19 | Sky Blue 5 | 2 | Eprs | 10090.ENSMUSP00000045841 | Bifunctional glutamate/proline--tRNA ligase; Multifunctional protein which is primarily part of the aminoacyl-tRNA synthetase multienzyme complex, also know as multisynthetase complex, that catalyzes the attachment of the cognate amino acid to the corresponding tRNA in a two-step reaction: the amino acid is first activated by ATP to form a covalent intermediate with AMP and is then transferred to the acceptor end of the cognate tRNA (By similarity). |
| 19 | Sky Blue 5 | 2 | Rars | 10090.ENSMUSP00000018992 | Arginine--tRNA ligase, cytoplasmic; Forms part of a macromolecular complex that catalyzes the attachment of specific amino acids to cognate tRNAs during protein synthesis. Modulates the secretion of AIMP1 and may be involved in generation of the inflammatory cytokine EMAP2 from AIMP1. |
| 20 | Sky Blue 3 | 2 | Galc | 10090.ENSMUSP00000021390 | Galactocerebrosidase; Hydrolyzes the galactose ester bonds of galactosylceramide, galactosylsphingosine, lactosylceramide, and monogalactosyldiglyceride. Enzyme with very low activity responsible for the lysosomal catabolism of galactosylceramide, a major lipid in myelin, kidney and epithelial cells of small intestine and colon. |
| 20 | Sky Blue 3 | 2 | Psap | 10090.ENSMUSP00000137476 | Saposin-B-Val; [Prosaposin]: Behaves as a myelinotrophic and neurotrophic factor, these effects are mediated by its G-protein-coupled receptors, GPR37 and GPR37L1, undergoing ligand-mediated internalization followed by ERK phosphorylation signaling. |
| 21 | Sky Blue | 2 | Fgf10 | 10090.ENSMUSP00000022246 | Fibroblast growth factor 10; Plays an important role in the regulation of embryonic development, cell proliferation and cell differentiation. Required for normal branching morphogenesis. May play a role in wound healing; Belongs to the heparin-binding growth factors family. |
| 21 | Sky Blue | 2 | Fgfr1 | 10090.ENSMUSP00000081041 | Fibroblast growth factor receptor 1; Tyrosine-protein kinase that acts as cell-surface receptor for fibroblast growth factors and plays an essential role in the regulation of embryonic development, cell proliferation, differentiation and migration. |
| 22 | Sky Blue 2 | 2 | Bnip3l | 10090.ENSMUSP00000022634 | BCL2/adenovirus E1B 19 kDa protein-interacting protein 3-like; Induces apoptosis. Interacts with viral and cellular anti- apoptosis proteins. |
| 22 | Sky Blue 2 | 2 | Steap3 | 10090.ENSMUSP00000108260 | Metalloreductase STEAP3; Endosomal ferrireductase required for efficient transferrin- dependent iron uptake in erythroid cells. Participates in erythroid iron homeostasis by reducing Fe(3+) to Fe(2+). Also mediates reduction of Cu(2+) to Cu(1+), suggesting that it participates in copper homeostasis. |
| 23 | Sky Blue 4 | 2 | Washc2 | 10090.ENSMUSP00000038983 | WASH complex subunit 2; Acts at least in part as component of the WASH core complex whose assembly at the surface of endosomes inhibits WASH nucleation- promoting factor (NPF) activity in recruiting and activating the Arp2/3 complex to induce actin polymerization and is involved in the fission of tubules that serve as transport intermediates during endosome sorting. |
| 23 | Sky Blue 4 | 2 | Washc5 | 10090.ENSMUSP00000022976 | WASH complex subunit 5; Acts at least in part as component of the WASH core complex whose assembly at the surface of endosomes seems to inhibit WASH nucleation-promoting factor (NPF) activity in recruiting and activating the Arp2/3 complex to induce actin polymerization, and which is involved in regulation of the fission of tubules that serve as transport intermediates during endosome sorting. |
| 24 | Cornflower Blue 3 | 2 | Got1 | 10090.ENSMUSP00000026196 | Aspartate aminotransferase, cytoplasmic; Biosynthesis of L-glutamate from L-aspartate or L-cysteine. Important regulator of levels of glutamate, the major excitatory neurotransmitter of the vertebrate central nervous system. Acts as a scavenger of glutamate in brain neuroprotection. |
| 24 | Cornflower Blue 3 | 2 | Got2 | 10090.ENSMUSP00000034097 | Aspartate aminotransferase, mitochondrial; Catalyzes the irreversible transamination of the L-tryptophan metabolite L-kynurenine to form kynurenic acid (KA). Plays a key role in amino acid metabolism. Important for metabolite exchange between mitochondria and cytosol. Facilitates cellular uptake of long-chain free fatty acids. |
| 25 | Cornflower Blue | 2 | Il6 | 10090.ENSMUSP00000026845 | Interleukin-6; Cytokine with a wide variety of biological functions. It is a potent inducer of the acute phase response. |
| 25 | Cornflower Blue | 2 | Tnfrsf1a | 10090.ENSMUSP00000032491 | Tumor necrosis factor receptor superfamily member 1A; Receptor for TNFSF2/TNF-alpha and homotrimeric TNFSF1/lymphotoxin-alpha. |
| 26 | Blue | 2 | Ssr1 | 10090.ENSMUSP00000021864 | Translocon-associated protein subunit alpha; TRAP proteins are part of a complex whose function is to bind calcium to the ER membrane and thereby regulate the retention of ER resident proteins. |
| 26 | Blue | 2 | Ssr3 | 10090.ENSMUSP00000029414 | Translocon-associated protein subunit gamma; TRAP proteins are part of a complex whose function is to bind calcium to the ER membrane and thereby regulate the retention of ER resident proteins; Belongs to the TRAP-gamma family. |
| 27 | Cornflower Blue 2 | 2 | Gclc | 10090.ENSMUSP00000034905 | Glutamate--cysteine ligase catalytic subunit; Belongs to the glutamate--cysteine ligase type 3 family. |
| 27 | Cornflower Blue 2 | 2 | Gclm | 10090.ENSMUSP00000029769 | Glutamate--cysteine ligase regulatory subunit; Belongs to the aldo/keto reductase family. Glutamate-- cysteine ligase light chain subfamily. |
| 28 | Medium Slate Blue | 2 | Pi4k2a | 10090.ENSMUSP00000069284 | Phosphatidylinositol 4-kinase type 2-alpha; Membrane-bound phosphatidylinositol-4 kinase (PI4-kinase) that catalyzes the phosphorylation of phosphatidylinositol (PI) to phosphatidylinositol 4-phosphate (PI4P), a lipid that plays important roles in endocytosis, Golgi function, protein sorting and membrane trafficking and is required for prolonged survival of neurons. |
| 28 | Medium Slate Blue | 2 | Pi4k2b | 10090.ENSMUSP00000031081 | Phosphatidylinositol 4-kinase type 2-beta; Together with PI4K2A and the type III PI4Ks (PIK4CA and PIK4CB) it contributes to the overall PI4-kinase activity of the cell. |
| 29 | Purple | 2 | Antxr1 | 10090.ENSMUSP00000045634 | Anthrax toxin receptor 1; Plays a role in cell attachment and migration. Interacts with extracellular matrix proteins and with the actin cytoskeleton. Mediates adhesion of cells to type 1 collagen and gelatin, reorganization of the actin cytoskeleton and promotes cell spreading. |
| 29 | Purple | 2 | Antxr2 | 10090.ENSMUSP00000031281 | Anthrax toxin receptor 2; Necessary for cellular interactions with laminin and the extracellular matrix; Belongs to the ATR family. |
| 30 | Medium Slate Blue 2 | 2 | Sec61a1 | 10090.ENSMUSP00000032168 | Protein transport protein Sec61 subunit alpha isoform 1; Component of SEC61 channel-forming translocon complex that mediates transport of signal peptide-containing precursor polypeptides across endoplasmic reticulum (ER). |
| 30 | Medium Slate Blue 2 | 2 | Sec61b | 10090.ENSMUSP00000067681 | Protein transport protein Sec61 subunit beta; Component of SEC61 channel-forming translocon complex that mediates transport of signal peptide-containing precursor polypeptides across endoplasmic reticulum (ER). |
| 31 | Medium Purple | 2 | Adm | 10090.ENSMUSP00000033054 | Proadrenomedullin N-20 terminal peptide; AM and PAMP are potent hypotensive and vasodilatator agents; Belongs to the adrenomedullin family. |
| 31 | Medium Purple | 2 | Ramp3 | 10090.ENSMUSP00000047518 | Receptor activity-modifying protein 3; Plays a role in cardioprotection by reducing cardiac hypertrophy and perivascular fibrosis in a GPER1-dependent manner. Transports the calcitonin gene-related peptide type 1 receptor (CALCRL) to the plasma membrane. Acts as a receptor for adrenomedullin (AM) together with CALCRL; Belongs to the RAMP family. |
| 32 | Medium Purple 2 | 2 | Mt1 | 10090.ENSMUSP00000034215 | Metallothionein-1; Metallothioneins have a high content of cysteine residues that bind various heavy metals; these proteins are transcriptionally regulated by both heavy metals and glucocorticoids; Belongs to the metallothionein superfamily. Type 1 family. |
| 32 | Medium Purple 2 | 2 | Mt2 | 10090.ENSMUSP00000034214 | Metallothionein-2; Metallothioneins have a high content of cysteine residues that bind various heavy metals; these proteins are transcriptionally regulated by both heavy metals and glucocorticoids; Belongs to the metallothionein superfamily. Type 1 family. |
| 33 | Medium Purple 3 | 2 | Anpep | 10090.ENSMUSP00000103015 | Aminopeptidase N; Broad specificity aminopeptidase which plays a role in the final digestion of peptides generated from hydrolysis of proteins by gastric and pancreatic proteases. Also involved in the processing of various peptides including peptide hormones, such as angiotensin III and IV, neuropeptides, and chemokines. |
| 33 | Medium Purple 3 | 2 | Lap3 | 10090.ENSMUSP00000040222 | Cytosol aminopeptidase; Presumably involved in the processing and regular turnover of intracellular proteins. Catalyses the removal of unsubstituted N- terminal amino acids from various peptides. |
| 34 | Orchid 5 | 2 | Grem1 | 10090.ENSMUSP00000097170 | Gremlin-1; Cytokine that may play an important role during carcinogenesis and metanephric kidney organogenesis, as BMP a antagonist required for early limb outgrowth and patterning in maintaining the FGF4-SHH feedback loop. Down-regulates the BMP4 signalling in a dose-dependent manner. |
| 34 | Orchid 5 | 2 | Grem2 | 10090.ENSMUSP00000049640 | Gremlin-2; Cytokine that inhibits the activity of BMP2 and BMP4 in a dose-dependent manner, and thereby modulates signalling by BMP family members. |
| 35 | Orchid 4 | 2 | Eps8 | 10090.ENSMUSP00000052776 | Epidermal growth factor receptor kinase substrate 8; Signalling adapter that controls various cellular protrusions by regulating actin cytoskeleton dynamics and architecture. |
| 35 | Orchid 4 | 2 | Nrn1 | 10090.ENSMUSP00000040900 | Neuritin; Promotes neurite outgrowth and especially branching of neuritic processes in primary hippocampal and cortical cells. |
| 36 | Orchid 2 | 2 | Chka | 10090.ENSMUSP00000025760 | Choline kinase alpha; Has a key role in phospholipid biosynthesis and may contribute to tumour cell growth. Catalyses the first step in phosphatidylcholine biosynthesis. Contributes to phosphatidylethanolamine biosynthesis. Phosphorylates choline and ethanolamine. Has higher activity with choline. Belongs to the choline/ethanolamine kinase family. |
| 36 | Orchid 2 | 2 | Phospho1 | 10090.ENSMUSP00000057858 | Phosphoethanolamine/phosphocholine phosphatase; Phosphatase that has a high activity toward phosphoethanolamine (PEA) and phosphocholine (PCho). Involved in the generation of inorganic phosphate for bone mineralization. |
| 37 | Violet 3 | 2 | Fosl1 | 10090.ENSMUSP00000025850 | Fos-related antigen 1; Belongs to the bZIP family. Fos subfamily. |
| 37 | Violet 3 | 2 | Junb | 10090.ENSMUSP00000064680 | Transcription factor jun-B; Transcription factor involved in regulating gene activity following the primary growth factor response. Binds to the DNA sequence 5'-TGA[CG]TCA-3'; Belongs to the bZIP family. Jun subfamily. |
| 38 | Violet | 2 | Nectin1 | 10090.ENSMUSP00000034510 | Nectin-1; Involved in cell adhesion and synaptogegesis. Has some neurite outgrowth-promoting activity. Receptor for alphaherpesvirus (HSV-1, HSV-2 and pseudorabies virus) entry into cells. Belongs to the nectin family. |
| 38 | Violet | 2 | Nectin2 | 10090.ENSMUSP00000074898 | Nectin-2; Modulator of T-cell signaling. Can be either a costimulator of T-cell function, or a coinhibitor, depending on the receptor it binds to. |
| 39 | Violet 2 | 2 | Ampd2 | 10090.ENSMUSP00000077946 | AMP deaminase 2; AMP deaminase plays a critical role in energy metabolism. Catalyzes the deamination of AMP to IMP and plays an important role in the purine nucleotide cycle (By similarity); Belongs to the metallo-dependent hydrolases superfamily. Adenosine and AMP deaminases family. |
| 39 | Violet 2 | 2 | Ampd3 | 10090.ENSMUSP00000005829 | AMP deaminase 3; AMP deaminase plays a critical role in energy metabolism; Belongs to the metallo-dependent hydrolases superfamily. Adenosine and AMP deaminases family. |
| 40 | Orchid | 2 | Pdxk | 10090.ENSMUSP00000038540 | Pyridoxal kinase; Required for synthesis of pyridoxal-5-phosphate from vitamin B6. |
| 40 | Orchid | 2 | Pdxp | 10090.ENSMUSP00000086796 | Pyridoxal phosphate phosphatase; Protein serine phosphatase that dephosphorylates 'Ser-3' in cofilin and probably also dephosphorylates phospho-serine residues in DSTN. Regulates cofilin-dependent actin cytoskeleton reorganization. |
| 41 | Orchid 3 | 2 | Lrrc8a | 10090.ENSMUSP00000092690 | Volume-regulated anion channel subunit LRRC8A; Essential component of the volume-regulated anion channel (VRAC, also named VSOAC channel), an anion channel required to maintain a constant cell volume in response to extracellular or intracellular osmotic changes. |
| 41 | Orchid 3 | 2 | Lrrc8d | 10090.ENSMUSP00000113603 | Volume-regulated anion channel subunit LRRC8D; Non-essential component of the volume-regulated anion channel (VRAC, also named VSOAC channel), an anion channel required to maintain a constant cell volume in response to extracellular or intracellular osmotic changes. |
| 42 | Hot Pink | 2 | Hmga2 | 10090.ENSMUSP00000123998 | High mobility group protein HMGI-C; Functions as a transcriptional regulator. Functions in cell cycle regulation through CCNA2. Plays an important role in chromosome condensation during the meiotic G2/M transition of spermatocytes. Plays a role in postnatal myogenesis, is involved in satellite cell activation. |
| 42 | Hot Pink | 2 | Igf2bp2 | 10090.ENSMUSP00000097629 | Insulin-like growth factor 2 mRNA-binding protein 2; RNA-binding factor that recruits target transcripts to cytoplasmic protein-RNA complexes (mRNPs). This transcript 'caging' into mRNPs allows mRNA transport and transient storage. |
| 43 | Pink | 2 | Ctsa | 10090.ENSMUSP00000099381 | Lysosomal protective protein 20 kDa chain; Protective protein appears to be essential for both the activity of beta-galactosidase and neuraminidase, it associates with these enzymes and exerts a protective function necessary for their stability and activity. This protein is also a carboxypeptidase and can deamidate tachykinins. |
| 43 | Pink | 2 | Neu1 | 10090.ENSMUSP00000007253 | Sialidase-1; Catalyzes the removal of sialic acid (N-acetylneuraminic acid) moieties from glycoproteins and glycolipids. To be active, it is strictly dependent on its presence in the multienzyme complex. Appears to have a preference for alpha 2-3 and alpha 2-6 sialyl linkage. Belongs to the glycosyl hydrolase 33 family. |
| 44 | Hot Pink 2 | 2 | Atg7 | 10090.ENSMUSP00000133215 | Ubiquitin-like modifier-activating enzyme ATG7; E1-like activating enzyme involved in the 2 ubiquitin-like systems required for cytoplasm to vacuole transport (Cvt) and autophagy. Activates ATG12 for its conjugation with ATG5 as well as the ATG8 family proteins for their conjugation with phosphatidylethanolamine. Both systems are needed for the ATG8 association to Cvt vesicles and autophagosomes membranes. Required for autophagic death induced by caspase-8 inhibition. Required for mitophagy. |
| 44 | Hot Pink 2 | 2 | Sirt1 | 10090.ENSMUSP00000112595 | NAD-dependent protein deacetylase sirtuin-1; NAD-dependent protein deacetylase that links transcriptional regulation directly to intracellular energetics and participates in the coordination of several separated cellular functions such as cell cycle, response to DNA damage, metabolism, apoptosis and autophagy. Can modulate chromatin function through deacetylation of histones and can promote alterations in the methylation of histones and DNA, leading to transcriptional repression. |
| 45 | Pale Violet Red | 2 | Nqo1 | 10090.ENSMUSP00000003947 | NAD(P)H dehydrogenase [quinone] 1; The enzyme apparently serves as a quinone reductase in connection with conjugation reactions of hydroquinons involved in detoxification pathways as well as in biosynthetic processes such as the vitamin K-dependent gamma-carboxylation of glutamate residues in prothrombin synthesis. |
| 45 | Pale Violet Red | 2 | Odc1 | 10090.ENSMUSP00000128661 | Ornithine decarboxylase; Catalyzes the first and rate-limiting step of polyamine biosynthesis that converts ornithine into putrescine, which is the precursor for the polyamines, spermidine and spermine. Polyamines are essential for cell proliferation and are implicated in cellular processes, ranging from DNA replication to apoptosis. |
| 46 | Light Coral 2 | 2 | Pfkfb2 | 10090.ENSMUSP00000066426 | 6-phosphofructo-2-kinase/fructose-2,6-bisphosphatase 2; Synthesis and degradation of fructose 2,6-bisphosphate. |
| 46 | Light Coral 2 | 2 | Pfkfb3 | 10090.ENSMUSP00000142079 | 6-phosphofructo-2-kinase/fructose-2, 6-biphosphatase 3 splice variant 2. |
| 47 | Light Coral | 2 | Nedd4l | 10090.ENSMUSP00000158026 | E3 ubiquitin-protein ligase NEDD4-like; E3 ubiquitin-protein ligase which accepts ubiquitin from an E2 ubiquitin-conjugating enzyme in the form of a thioester and then directly transfers the ubiquitin to targeted substrates. Inhibits TGF- beta signalling by triggering SMAD2 and TGFBR1 ubiquitination and proteasome-dependent degradation. |
| 47 | Light Coral | 2 | Sgk1 | 10090.ENSMUSP00000114074 | Serine/threonine-protein kinase Sgk1; Serine/threonine-protein kinase which is involved in the regulation of a wide variety of ion channels, membrane transporters, cellular enzymes, transcription factors, neuronal excitability, cell growth, proliferation, survival, migration and apoptosis. Plays an important role in cellular stress response. |

**Supplementary Table 3**.

| **Cluster number** | **Cluster colour** | **Gene count** | **Protein name** | **Protein identifier** | **Protein description** |
| --- | --- | --- | --- | --- | --- |
| 1 | Red | 10 | Acta1 | 10090.ENSMUSP00000034453 | Actin, alpha skeletal muscle, intermediate form; Actins are highly conserved proteins that are involved in various types of cell motility and are ubiquitously expressed in all eukaryotic cells. |
| 1 | Red | 10 | Actc1 | 10090.ENSMUSP00000087736 | Actin, alpha cardiac muscle 1, intermediate form; Actins are highly conserved proteins that are involved in various types of cell motility and are ubiquitously expressed in all eukaryotic cells; Belongs to the actin family. |
| 1 | Red | 10 | Baiap2 | 10090.ENSMUSP00000026436 | Brain-specific angiogenesis inhibitor 1-associated protein 2; Adapter protein that links membrane-bound small G-proteins to cytoplasmic effector proteins. Necessary for CDC42-mediated reorganization of the actin cytoskeleton and for RAC1-mediated membrane ruffling. |
| 1 | Red | 10 | Myh4 | 10090.ENSMUSP00000018632 | Myosin-4; Muscle contraction. |
| 1 | Red | 10 | Myl1 | 10090.ENSMUSP00000027151 | Myosin light chain 1/3, skeletal muscle isoform; Non-regulatory myosin light chain required for proper formation and/or maintenance of myofibers, and thus appropriate muscle function. |
| 1 | Red | 10 | Myl4 | 10090.ENSMUSP00000102570 | Myosin light chain 4; Regulatory light chain of myosin. Does not bind calcium. |
| 1 | Red | 10 | Mylpf | 10090.ENSMUSP00000032910 | Myosin regulatory light chain 2, skeletal muscle isoform. |
| 1 | Red | 10 | Tpm1 | 10090.ENSMUSP00000109337 | Tropomyosin alpha-1 chain; Binds to actin filaments in muscle and non-muscle cells. Plays a central role, in association with the troponin complex, in the calcium dependent regulation of vertebrate striated muscle contraction. Smooth muscle contraction is regulated by interaction with caldesmon. In non-muscle cells is implicated in stabilizing cytoskeleton actin filaments. |
| 1 | Red | 10 | Tpm2 | 10090.ENSMUSP00000103546 | Tropomyosin beta chain; Binds to actin filaments in muscle and non-muscle cells. Plays a central role, in association with the troponin complex, in the calcium dependent regulation of vertebrate striated muscle contraction. Smooth muscle contraction is regulated by interaction with caldesmon. In non-muscle cells is implicated in stabilizing cytoskeleton actin filaments. The non-muscle isoform may have a role in agonist-mediated receptor internalization. Belongs to the tropomyosin family. |
| 1 | Red | 10 | Vasp | 10090.ENSMUSP00000032561 | Vasodilator-stimulated phosphoprotein; Ena/VASP proteins are actin-associated proteins involved in a range of processes dependent on cytoskeleton remodelling and cell polarity such as axon guidance, lamellipodial and filopodial dynamics, platelet activation and cell migration. |
| 2 | Salmon | 5 | Acta2 | 10090.ENSMUSP00000048218 | Actin, aortic smooth muscle, intermediate form; Actins are highly conserved proteins that are involved in various types of cell motility and are ubiquitously expressed in all eukaryotic cells. |
| 2 | Salmon | 5 | Actg2 | 10090.ENSMUSP00000074658 | Actin, gamma-enteric smooth muscle, intermediate form; Actins are highly conserved proteins that are involved in various types of cell motility and are ubiquitously expressed in all eukaryotic cells; Belongs to the actin family. |
| 2 | Salmon | 5 | Myl12a | 10090.ENSMUSP00000123412 | Myosin, light chain 12A, regulatory, non-sarcomeric. |
| 2 | Salmon | 5 | Myl6 | 10090.ENSMUSP00000128803 | Myosin light polypeptide 6; Regulatory light chain of myosin. Does not bind calcium. |
| 2 | Salmon | 5 | Myl9 | 10090.ENSMUSP00000085913 | Myosin regulatory light polypeptide 9; Myosin regulatory subunit that plays an important role in regulation of both smooth muscle and non-musical cell contractile activity via its phosphorylation. Implicated in cytokinesis, receptor capping, and cell locomotion. |
| 3 | Saddle Brown | 5 | Dag1 | 10090.ENSMUSP00000142109 | Alpha-dystroglycan; The dystroglycan complex is involved in a number of processes including laminin and basement membrane assembly, sacrolemmal stability, cell survival, peripheral nerve myelination, nodal structure, cell migration, and epithelial polarization. |
| 3 | Saddle Brown | 5 | Nrxn3 | 10090.ENSMUSP00000129678 | Neurexin-3; Neuronal cell surface protein that may be involved in cell recognition and cell adhesion. May mediate intracellular signaling (By similarity). |
| 3 | Saddle Brown | 5 | Pgm5 | 10090.ENSMUSP00000036025 | Phosphoglucomutase-like protein 5; Component of adherens-type cell-cell and cell-matrix junctions. Lacks phosphoglucomutase activity (By similarity). Belongs to the phosphohexose mutase family. |
| 3 | Saddle Brown | 5 | Sgcg | 10090.ENSMUSP00000077106 | Gamma-sarcoglycan; Component of the sarcoglycan complex, a subcomplex of the dystrophin-glycoprotein complex which forms a link between the F-actin cytoskeleton and the extracellular matrix. |
| 3 | Saddle Brown | 5 | Sspn | 10090.ENSMUSP00000032383 | Sarcospan; Component of the dystrophin-glycoprotein complex (DGC), a complex that spans the muscle plasma membrane and forms a link between the F-actin cytoskeleton and the extracellular matrix. Preferentially associates with the sarcoglycan subcomplex of the DGC (By similarity). |
| 4 | Sandy Brown | 4 | Tnnc1 | 10090.ENSMUSP00000131991 | Troponin C, slow skeletal and cardiac muscles; Troponin is the central regulatory protein of striated muscle contraction. Tn consists of three components: Tn-I which is the inhibitor of actomyosin ATPase, Tn-T which contains the binding site for tropomyosin and Tn-C. The binding of calcium to Tn-C abolishes the inhibitory action of Tn on actin filaments. |
| 4 | Sandy Brown | 4 | Tnnc2 | 10090.ENSMUSP00000099384 | Troponin C, skeletal muscle; Troponin is the central regulatory protein of striated muscle contraction. Tn consists of three components: Tn-I which is the inhibitor of actomyosin ATPase, Tn-T which contains the binding site for tropomyosin and Tn-C. The binding of calcium to Tn-C abolishes the inhibitory action of Tn on actin filaments. |
| 4 | Sandy Brown | 4 | Tnni2 | 10090.ENSMUSP00000122733 | Troponin I, fast skeletal muscle; Troponin I is the inhibitory subunit of troponin, the thin filament regulatory complex which confers calcium-sensitivity to striated muscle actomyosin ATPase activity. |
| 4 | Sandy Brown | 4 | Tnnt2 | 10090.ENSMUSP00000140941 | Troponin T, cardiac muscle; Troponin T is the tropomyosin-binding subunit of troponin, the thin filament regulatory complex which confers calcium-sensitivity to striated muscle actomyosin ATPase activity. |
| 5 | Brown | 3 | Tgfb2 | 10090.ENSMUSP00000142149 | Transforming growth factor beta-2 proprotein; Transforming growth factor beta-2 proprotein: Precursor of the Latency-associated peptide (LAP) and Transforming growth factor beta-2 (TGF-beta-2) chains, which constitute the regulatory and active subunit of TGF-beta-2, respectively. Transforming growth factor beta-2: Multifunctional protein that regulates various processes such as angiogenesis and heart development. |
| 5 | Brown | 3 | Tgfb3 | 10090.ENSMUSP00000003687 | Transforming growth factor beta-3 proprotein; Transforming growth factor beta-3 proprotein: Precursor of the Latency-associated peptide (LAP) and Transforming growth factor beta-3 (TGF-beta-3) chains, which constitute the regulatory and active subunit of TGF-beta-3, respectively. Transforming growth factor beta-3: Multifunctional protein that regulates embryogenesis and cell differentiation and is required in various processes such as secondary palate development. |
| 5 | Brown | 3 | Tgfbr2 | 10090.ENSMUSP00000062333 | TGF-beta receptor type-2; Transmembrane serine/threonine kinase forming with the TGF- beta type I serine/threonine kinase receptor, TGFBR1, the non- promiscuous receptor for the TGF-beta cytokines TGFB1, TGFB2 and TGFB3. Transduces the TGFB1, TGFB2 and TGFB3 signal from the cell surface to the cytoplasm and is thus regulating a plethora of physiological and pathological processes. |
| 6 | Yellow | 3 | Chrna1 | 10090.ENSMUSP00000028515 | Acetylcholine receptor subunit alpha; After binding acetylcholine, the AChR responds by an extensive change in conformation that affects all subunits and leads to opening of an ion-conducting channel across the plasma membrane. |
| 6 | Yellow | 3 | Chrnb1 | 10090.ENSMUSP00000047270 | Acetylcholine receptor subunit beta; After binding acetylcholine, the AChR responds by an extensive change in conformation that affects all subunits and leads to opening of an ion-conducting channel across the plasma membrane. |
| 6 | Yellow | 3 | Chrng | 10090.ENSMUSP00000027470 | Acetylcholine receptor subunit gamma; After binding acetylcholine, the AChR responds by an extensive change in conformation that affects all subunits and leads to opening of an ion-conducting channel across the plasma membrane; Belongs to the ligand-gated ion channel (TC 1.A.9) family. Acetylcholine receptor (TC 1.A.9.1) subfamily. Gamma/CHRNG sub- subfamily. |
| 7 | Olive | 3 | Casq2 | 10090.ENSMUSP00000029454 | Calsequestrin-2; Calsequestrin is a high-capacity, moderate affinity, calcium- binding protein and thus acts as an internal calcium store in muscle. Calcium ions are bound by clusters of acidic residues at the protein surface, especially at the interface between subunits. |
| 7 | Olive | 3 | Jph1 | 10090.ENSMUSP00000039072 | Junctophilin-1; Junctophilins contribute to the formation of junctional membrane complexes (JMCs) which link the plasma membrane with the endoplasmic or sarcoplasmic reticulum in excitable cells. Provides a structural foundation for functional cross-talk between the cell surface and intracellular calcium release channels. JPH1 contributes to the construction of the skeletal muscle triad by linking the t-tubule (transverse-tubule) and SR (sarcoplasmic reticulum) membranes. |
| 7 | Olive | 3 | Ryr3 | 10090.ENSMUSP00000147250 | Ryanodine receptor 3; Calcium channel that mediates the release of Ca(2+) from the sarcoplasmic reticulum into the cytoplasm in muscle and thereby plays a role in triggering muscle contraction. May regulate Ca(2+) release by other calcium channels. Calcium channel that mediates Ca(2+)-induced Ca(2+) release from the endoplasmic reticulum in non-muscle cells. Plays a role in cellular calcium signaling. Contributes to cellular calcium ion homeostasis. |
| 8 | Green | 3 | Myod1 | 10090.ENSMUSP00000072330 | Myoblast determination protein 1; Acts as a transcriptional activator that promotes transcription of muscle-specific target genes and plays a role in muscle differentiation. Together with MYF5 and MYOG, co-occupies muscle-specific gene promoter core region during myogenesis. |
| 8 | Green | 3 | Smarcd3 | 10090.ENSMUSP00000030791 | SWI/SNF-related matrix-associated actin-dependent regulator of chromatin subfamily D member 3; Involved in transcriptional activation and repression of select genes by chromatin remodeling (alteration of DNA-nucleosome topology). |
| 8 | Green | 3 | Tcf4 | 10090.ENSMUSP00000110636 | Transcription factor 4; Transcription factor that binds to the immunoglobulin enhancer Mu-E5/KE5-motif. Involved in the initiation of neuronal differentiation. Activates transcription by binding to the E box (5'- CANNTG-3'). Isoform 2 inhibits MYOD1 activation of the cardiac alpha- actin promoter. |
| 9 | Lime Green | 3 | Igf1 | 10090.ENSMUSP00000100937 | Insulin-like growth factor I; The insulin-like growth factors, isolated from plasma, are structurally and functionally related to insulin but have a much higher growth-promoting activity. |
| 9 | Lime Green | 3 | Igfbp5 | 10090.ENSMUSP00000027377 | Insulin-like growth factor-binding protein 5; IGF-binding proteins prolong the half-life of the IGFs and have been shown to either inhibit or stimulate the growth promoting effects of the IGFs on cell culture. They alter the interaction of IGFs with their cell surface receptors. |
| 9 | Lime Green | 3 | Irs1 | 10090.ENSMUSP00000063795 | Insulin receptor substrate 1; May mediate the control of various cellular processes by insulin. When phosphorylated by the insulin receptor binds specifically to various cellular proteins containing SH2 domains such as phosphatidylinositol 3-kinase p85 subunit or GRB2. Activates phosphatidylinositol 3-kinase when bound to the regulatory p85 subunit (By similarity). |
| 10 | Light Green | 2 | Col6a1 | 10090.ENSMUSP00000001147 | Collagen alpha-1(VI) chain; Collagen VI acts as a cell-binding protein; Belongs to the type VI collagen family. |
| 10 | Light Green | 2 | Col6a2 | 10090.ENSMUSP00000001181 | Collagen alpha-2(VI) chain; Collagen VI acts as a cell-binding protein. |
| 11 | Sky Blue | 2 | Col3a1 | 10090.ENSMUSP00000085192 | Collagen alpha-1(III) chain; Collagen type III occurs in most soft connective tissues along with type I collagen. Involved in regulation of cortical development. |
| 11 | Sky Blue | 2 | Col5a3 | 10090.ENSMUSP00000004201 | Collagen type V alpha 3 chain. |
| 12 | Cornflower Blue | 2 | Cav1 | 10090.ENSMUSP00000007799 | Caveolin-1; May act as a scaffolding protein within caveolar membranes (By similarity). Forms a stable heterooligomeric complex with CAV2 that targets to lipid rafts and drives caveolae formation. Mediates the recruitment of CAVIN proteins (CAVIN1/2/3/4) to the caveolae. Interacts directly with G-protein alpha subunits and can functionally regulate their activity. |
| 12 | Cornflower Blue | 2 | Cavin1 | 10090.ENSMUSP00000058321 | Caveolae-associated protein 1; Plays an important role in caveolae formation and organization. Essential for the formation of caveolae in all tissues. Core component of the CAVIN complex which is essential for recruitment of the complex to the caveolae in presence of calveolin-1 (CAV1). |
| 13 | Blue | 2 | Cd82 | 10090.ENSMUSP00000028644 | CD82 antigen; Associates with CD4 or CD8 and delivers costimulatory signals for the TCR/CD3 pathway; Belongs to the tetraspanin (TM4SF) family. |
| 13 | Blue | 2 | Cyb5d2 | 10090.ENSMUSP00000078623 | Neuferricin; Heme-binding protein which promotes neuronal but not astrocyte differentiation. |
| 14 | Purple | 2 | Kcnj12 | 10090.ENSMUSP00000041696 | ATP-sensitive inward rectifier potassium channel 12; Inward rectifying potassium channel that is activated by phosphatidylinositol 4,5-bisphosphate and that probably participates in controlling the resting membrane potential in electrically excitable cells. |
| 14 | Purple | 2 | Kcnq4 | 10090.ENSMUSP00000030376 | Potassium voltage-gated channel subfamily KQT member 4; Probably important in the regulation of neuronal excitability. |
| 15 | Medium Purple | 2 | Atp2a1 | 10090.ENSMUSP00000032974 | Sarcoplasmic/endoplasmic reticulum calcium ATPase 1; Key regulator of striated muscle performance by acting as the major Ca(2+) ATPase responsible for the reuptake of cytosolic Ca(2+) into the sarcoplasmic reticulum. |
| 15 | Medium Purple | 2 | Hrc | 10090.ENSMUSP00000082459 | Histidine-rich calcium-binding protein. |
| 16 | Medium Purple 2 | 2 | Lpar3 | 10090.ENSMUSP00000037712 | Lysophosphatidic acid receptor 3; Receptor for lysophosphatidic acid (LPA), a mediator of diverse cellular activities. Seems to be coupled to the G(i)/G(o) and G(q) families of heteromeric G proteins. |
| 16 | Medium Purple 2 | 2 | Lpar4 | 10090.ENSMUSP00000053986 | Lysophosphatidic acid receptor 4; Receptor for lysophosphatidic acid (LPA), a mediator of diverse cellular activities. |
| 17 | Orchid 2 | 2 | Fyn | 10090.ENSMUSP00000097547 | Tyrosine-protein kinase Fyn; Non-receptor tyrosine-protein kinase that plays a role in many biological processes including regulation of cell growth and survival, cell adhesion, integrin-mediated signaling, cytoskeletal remodeling, cell motility, immune response and axon guidance. |
| 17 | Orchid 2 | 2 | Ncam1 | 10090.ENSMUSP00000130668 | Neural cell adhesion molecule 1; This protein is a cell adhesion molecule involved in neuron- neuron adhesion, neurite fasciculation, outgrowth of neurites, etc. |
| 18 | Violet | 2 | Ndufa13 | 10090.ENSMUSP00000105796 | NADH dehydrogenase [ubiquinone] 1 alpha subcomplex subunit 13; Accessory subunit of the mitochondrial membrane respiratory chain NADH dehydrogenase (Complex I), that is believed not to be involved in catalysis. Complex I functions in the transfer of electrons from NADH to the respiratory chain. |
| 18 | Violet | 2 | Ndufb4 | 10090.ENSMUSP00000023514 | NADH dehydrogenase [ubiquinone] 1 beta subcomplex subunit 4; Accessory subunit of the mitochondrial membrane respiratory chain NADH dehydrogenase (Complex I), that is believed not to be involved in catalysis. Complex I functions in the transfer of electrons from NADH to the respiratory chain. The immediate electron acceptor for the enzyme is believed to be ubiquinone. |
| 19 | Violet 2 | 2 | Cacna1s | 10090.ENSMUSP00000107695 | Voltage-dependent L-type calcium channel subunit alpha-1S; Pore-forming, alpha-1S subunit of the voltage-gated calcium channel that gives rise to L-type calcium currents in skeletal muscle. |
| 19 | Violet 2 | 2 | Cacng1 | 10090.ENSMUSP00000021065 | Voltage-dependent calcium channel gamma-1 subunit; Regulatory subunit of the voltage-gated calcium channel that gives rise to L-type calcium currents in skeletal muscle. Regulates channel inactivation kinetics; Belongs to the PMP-22/EMP/MP20 family. CACNG subfamily. |
| 20 | Orchid | 2 | Cdon | 10090.ENSMUSP00000113977 | Cell adhesion molecule-related/down-regulated by oncogenes; Component of a cell-surface receptor complex that mediates cell-cell interactions between muscle precursor cells. Promotes differentiation of myogenic cells. Required for response to NTN3 and activation of NFATC3. |
| 20 | Orchid | 2 | Gas1 | 10090.ENSMUSP00000153311 | Growth arrest-specific protein 1; Specific growth arrest protein involved in growth suppression. Blocks entry to S phase. Prevents cycling of normal and transformed cells. |
| 21 | Hot Pink | 2 | Mef2a | 10090.ENSMUSP00000117496 | Myocyte-specific enhancer factor 2A; Transcriptional activator which binds specifically to the MEF2 element, 5'-YTA[AT](4)TAR-3', found in numerous muscle-specific genes. Also involved in the activation of numerous growth factor- and stress-induced genes. Mediates cellular functions not only in skeletal and cardiac muscle development, but also in neuronal differentiation and survival. |
| 21 | Hot Pink | 2 | Mef2c | 10090.ENSMUSP00000143401 | Myocyte-specific enhancer factor 2C; Transcription activator which binds specifically to the MEF2 element present in the regulatory regions of many muscle-specific genes. Controls cardiac morphogenesis and myogenesis, and is also involved in vascular development. Enhances transcriptional activation mediated by SOX18. |
| 22 | Pink | 2 | Ckb | 10090.ENSMUSP00000001304 | Creatine kinase B-type; Reversibly catalyzes the transfer of phosphate between ATP and various phosphogens (e.g. creatine phosphate). Creatine kinase isoenzymes play a central role in energy transduction in tissues with large, fluctuating energy demands, such as skeletal muscle, heart, brain and spermatozoa; Belongs to the ATP:guanido phosphotransferase family. |
| 22 | Pink | 2 | Ckm | 10090.ENSMUSP00000146972 | Creatine kinase M-type; Reversibly catalyzes the transfer of phosphate between ATP and various phosphogens (e.g. creatine phosphate). Creatine kinase isoenzymes play a central role in energy transduction in tissues with large, fluctuating energy demands, such as skeletal muscle, heart, brain and spermatozoa; Belongs to the ATP:guanido phosphotransferase family. |

**Supplementary Table 4.** Common up motifs

| **Motif Name** | **Consensus** | **P-value** | **Log P-value** | **q-value (Benjamini)** | **# of Target Sequences with Motif(of 724)** | **% of Target Sequences with Motif** | **# of Background Sequences with Motif(of 27615)** | **% of Background Sequences with Motif** |
| --- | --- | --- | --- | --- | --- | --- | --- | --- |
| USF1(bHLH)/GM12878-Usf1-ChIP-Seq(GSE32465)/Homer | SGTCACGTGR | 1.00E-08 | -1.93E+01 | 0 | 265 | 36.60% | 7351.2 | 26.62% |
| bHLHE40(bHLH)/HepG2-BHLHE40-ChIP-Seq(GSE31477)/Homer | KCACGTGMCN | 1.00E-05 | -1.38E+01 | 0.0001 | 192 | 26.52% | 5281.8 | 19.13% |
| CLOCK(bHLH)/Liver-Clock-ChIP-Seq(GSE39860)/Homer | GHCACGTG | 1.00E-05 | -1.28E+01 | 0.0003 | 285 | 39.36% | 8620.7 | 31.22% |
| Sp1(Zf)/Promoter/Homer | GGCCCCGCCCCC | 1.00E-05 | -1.25E+01 | 0.0003 | 356 | 49.17% | 11253.1 | 40.75% |
| n-Myc(bHLH)/mES-nMyc-ChIP-Seq(GSE11431)/Homer | VRCCACGTGG | 1.00E-05 | -1.22E+01 | 0.0003 | 321 | 44.34% | 9983.1 | 36.15% |
| c-Myc(bHLH)/LNCAP-cMyc-ChIP-Seq(Unpublished)/Homer | VCCACGTG | 1.00E-05 | -1.22E+01 | 0.0003 | 270 | 37.29% | 8143 | 29.49% |
| Max(bHLH)/K562-Max-ChIP-Seq(GSE31477)/Homer | RCCACGTGGYYN | 1.00E-05 | -1.22E+01 | 0.0003 | 304 | 41.99% | 9371.6 | 33.94% |
| c-Myc(bHLH)/mES-cMyc-ChIP-Seq(GSE11431)/Homer | VVCCACGTGG | 1.00E-04 | -1.05E+01 | 0.0009 | 238 | 32.87% | 7177.6 | 25.99% |
| E-box(bHLH)/Promoter/Homer | SSGGTCACGTGA | 1.00E-04 | -9.55E+00 | 0.0021 | 76 | 10.50% | 1823.7 | 6.60% |
| Usf2(bHLH)/C2C12-Usf2-ChIP-Seq(GSE36030)/Homer | GTCACGTGGT | 1.00E-03 | -8.91E+00 | 0.0036 | 174 | 24.03% | 5098.2 | 18.46% |
| HIF-1b(HLH)/T47D-HIF1b-ChIP-Seq(GSE59937)/Homer | RTACGTGC | 1.00E-03 | -8.34E+00 | 0.0057 | 454 | 62.71% | 15505 | 56.15% |
| BMAL1(bHLH)/Liver-Bmal1-ChIP-Seq(GSE39860)/Homer | GNCACGTG | 1.00E-03 | -7.73E+00 | 0.0097 | 543 | 75.00% | 19122.7 | 69.25% |
| NPAS2(bHLH)/Liver-NPAS2-ChIP-Seq(GSE39860)/Homer | KCCACGTGAC | 1.00E-03 | -7.51E+00 | 0.0111 | 409 | 56.49% | 13885 | 50.29% |
| SpiB(ETS)/OCILY3-SPIB-ChIP-Seq(GSE56857)/Homer | AAAGRGGAAGTG | 1.00E-02 | -6.63E+00 | 0.025 | 148 | 20.44% | 4442.5 | 16.09% |
| Atf3(bZIP)/GBM-ATF3-ChIP-Seq(GSE33912)/Homer | DATGASTCATHN | 1.00E-02 | -6.30E+00 | 0.0324 | 256 | 35.36% | 8339.4 | 30.20% |
| PU.1(ETS)/ThioMac-PU.1-ChIP-Seq(GSE21512)/Homer | AGAGGAAGTG | 1.00E-02 | -6.12E+00 | 0.0362 | 265 | 36.60% | 8695.6 | 31.49% |
| AP-1(bZIP)/ThioMac-PU.1-ChIP-Seq(GSE21512)/Homer | VTGACTCATC | 1.00E-02 | -6.01E+00 | 0.0381 | 280 | 38.67% | 9264.9 | 33.55% |
| Olig2(bHLH)/Neuron-Olig2-ChIP-Seq(GSE30882)/Homer | RCCATMTGTT | 1.00E-02 | -5.93E+00 | 0.0391 | 564 | 77.90% | 20231.9 | 73.27% |
| KLF5(Zf)/LoVo-KLF5-ChIP-Seq(GSE49402)/Homer | DGGGYGKGGC | 1.00E-02 | -5.88E+00 | 0.0391 | 593 | 81.91% | 21421.2 | 77.58% |
| Klf4(Zf)/mES-Klf4-ChIP-Seq(GSE11431)/Homer | GCCACACCCA | 1.00E-02 | -5.50E+00 | 0.0539 | 283 | 39.09% | 9457.6 | 34.25% |
| AMYB(HTH)/Testes-AMYB-ChIP-Seq(GSE44588)/Homer | TGGCAGTTGG | 1.00E-02 | -4.98E+00 | 0.0861 | 543 | 75.00% | 19539.2 | 70.76% |
| CRX(Homeobox)/Retina-Crx-ChIP-Seq(GSE20012)/Homer | GCTAATCC | 1.00E-02 | -4.96E+00 | 0.0861 | 634 | 87.57% | 23255 | 84.22% |
| Fra1(bZIP)/BT549-Fra1-ChIP-Seq(GSE46166)/Homer | NNATGASTCATH | 1.00E-02 | -4.92E+00 | 0.0861 | 218 | 30.11% | 7167 | 25.96% |
| ELF5(ETS)/T47D-ELF5-ChIP-Seq(GSE30407)/Homer | ACVAGGAAGT | 1.00E-02 | -4.79E+00 | 0.0914 | 357 | 49.31% | 12355.6 | 44.75% |
| NFkB-p65-Rel(RHD)/ThioMac-LPS-Expression(GSE23622)/Homer | GGAAATTCCC | 1.00E-02 | -4.76E+00 | 0.0914 | 40 | 5.52% | 1012.6 | 3.67% |
| Nkx6.1(Homeobox)/Islet-Nkx6.1-ChIP-Seq(GSE40975)/Homer | GKTAATGR | 1.00E-01 | -4.44E+00 | 0.1197 | 630 | 87.02% | 23162.4 | 83.88% |
| Isl1(Homeobox)/Neuron-Isl1-ChIP-Seq(GSE31456)/Homer | CTAATKGV | 1.00E-01 | -4.39E+00 | 0.1208 | 589 | 81.35% | 21490.4 | 77.83% |
| Fosl2(bZIP)/3T3L1-Fosl2-ChIP-Seq(GSE56872)/Homer | NATGASTCABNN | 1.00E-01 | -4.28E+00 | 0.131 | 136 | 18.78% | 4319.9 | 15.64% |
| BATF(bZIP)/Th17-BATF-ChIP-Seq(GSE39756)/Homer | DATGASTCAT | 1.00E-01 | -4.11E+00 | 0.1497 | 249 | 34.39% | 8446.1 | 30.59% |
| ETS:RUNX(ETS,Runt)/Jurkat-RUNX1-ChIP-Seq(GSE17954)/Homer | RCAGGATGTGGT | 1.00E-01 | -4.04E+00 | 0.1544 | 63 | 8.70% | 1820.6 | 6.59% |
| EWS:ERG-fusion(ETS)/CADO_ES1-EWS:ERG-ChIP-Seq(SRA014231)/Homer | ATTTCCTGTN | 1.00E-01 | -3.90E+00 | 0.1732 | 329 | 45.44% | 11476.6 | 41.56% |
| Srebp1a(bHLH)/HepG2-Srebp1a-ChIP-Seq(GSE31477)/Homer | RTCACSCCAY | 1.00E-01 | -3.90E+00 | 0.1732 | 111 | 15.33% | 3491.1 | 12.64% |
| PPARE(NR),DR1/3T3L1-Pparg-ChIP-Seq(GSE13511)/Homer | TGACCTTTGCCCCA | 1.00E-01 | -3.71E+00 | 0.1951 | 397 | 54.83% | 14098.7 | 51.06% |
| SCL(bHLH)/HPC7-Scl-ChIP-Seq(GSE13511)/Homer | AVCAGCTG | 1.00E-01 | -3.66E+00 | 0.1992 | 704 | 97.24% | 26440.5 | 95.76% |
| RXR(NR),DR1/3T3L1-RXR-ChIP-Seq(GSE13511)/Homer | TAGGGCAAAGGTCA | 1.00E-01 | -3.61E+00 | 0.2034 | 439 | 60.64% | 15734.8 | 56.98% |
| Ascl1(bHLH)/NeuralTubes-Ascl1-ChIP-Seq(GSE55840)/Homer | NNVVCAGCTGBN | 1.00E-01 | -3.55E+00 | 0.2098 | 494 | 68.23% | 17882.9 | 64.76% |
| HRE(HSF)/Striatum-HSF1-ChIP-Seq(GSE38000)/Homer | TTCTAGAABNTTCTA | 1.00E-01 | -3.47E+00 | 0.2211 | 127 | 17.54% | 4121.6 | 14.93% |
| MafK(bZIP)/C2C12-MafK-ChIP-Seq(GSE36030)/Homer | GCTGASTCAGCA | 1.00E-01 | -3.43E+00 | 0.2255 | 99 | 13.67% | 3135.5 | 11.36% |
| HOXD13(Homeobox)/Chicken-Hoxd13-ChIP-Seq(GSE38910)/Homer | NCYAATAAAA | 1.00E-01 | -3.36E+00 | 0.2343 | 416 | 57.46% | 14906.9 | 53.99% |
| HIF-1a(bHLH)/MCF7-HIF1a-ChIP-Seq(GSE28352)/Homer | TACGTGCV | 1.00E-01 | -3.20E+00 | 0.2693 | 134 | 18.51% | 4418.5 | 16.00% |
| Ets1-distal(ETS)/CD4+-PolII-ChIP-Seq(Barski et al.)/Homer | MACAGGAAGT | 1.00E-01 | -3.04E+00 | 0.3084 | 160 | 22.10% | 5389.7 | 19.52% |
| p53(p53)/mES-cMyc-ChIP-Seq(GSE11431)/Homer | ACATGCCCGGGCAT | 1.00E-01 | -3.00E+00 | 0.3142 | 11 | 1.52% | 233 | 0.84% |
| CTCF(Zf)/CD4+-CTCF-ChIP-Seq(Barski et al.)/Homer | AYAGTGCCMYCTRGTGGCCA | 1.00E-01 | -2.94E+00 | 0.3258 | 87 | 12.02% | 2784.3 | 10.08% |
| CRE(bZIP)/Promoter/Homer | CSGTGACGTCAC | 1.00E-01 | -2.89E+00 | 0.3335 | 140 | 19.34% | 4692 | 16.99% |
| Stat3(Stat)/mES-Stat3-ChIP-Seq(GSE11431)/Homer | CTTCCGGGAA | 1.00E-01 | -2.89E+00 | 0.3335 | 264 | 36.46% | 9264.9 | 33.55% |
| Tcfcp2l1(CP2)/mES-Tcfcp2l1-ChIP-Seq(GSE11431)/Homer | NRAACCRGTTYRAACCRGYT | 1.00E-01 | -2.88E+00 | 0.3335 | 86 | 11.88% | 2757.9 | 9.99% |
| BORIS(Zf)/K562-CTCFL-ChIP-Seq(GSE32465)/Homer | CNNBRGCGCCCCCTGSTGGC | 1.00E-01 | -2.72E+00 | 0.3718 | 134 | 18.51% | 4507.3 | 16.32% |
| CEBP:AP1(bZIP)/ThioMac-CEBPb-ChIP-Seq(GSE21512)/Homer | DRTGTTGCAA | 1.00E-01 | -2.70E+00 | 0.3718 | 294 | 40.61% | 10436.6 | 37.80% |
| Znf263(Zf)/K562-Znf263-ChIP-Seq(GSE31477)/Homer | CVGTSCTCCC | 1.00E-01 | -2.70E+00 | 0.3718 | 566 | 78.18% | 20904.9 | 75.71% |
| NF1:FOXA1(CTF,Forkhead)/LNCAP-FOXA1-ChIP-Seq(GSE27824)/Homer | WNTGTTTRYTTTGGCA | 1.00E-01 | -2.69E+00 | 0.3718 | 30 | 4.14% | 849.2 | 3.08% |
| MYB(HTH)/ERMYB-Myb-ChIPSeq(GSE22095)/Homer | GGCVGTTR | 1.00E-01 | -2.66E+00 | 0.3718 | 573 | 79.14% | 21189 | 76.74% |
| MITF(bHLH)/MastCells-MITF-ChIP-Seq(GSE48085)/Homer | RTCATGTGAC | 1.00E-01 | -2.63E+00 | 0.3718 | 357 | 49.31% | 12837.8 | 46.49% |
| Rbpj1(?)/Panc1-Rbpj1-ChIP-Seq(GSE47459)/Homer | HTTTCCCASG | 1.00E-01 | -2.62E+00 | 0.3718 | 520 | 71.82% | 19118.9 | 69.24% |
| HNF4a(NR),DR1/HepG2-HNF4a-ChIP-Seq(GSE25021)/Homer | CARRGKBCAAAGTYCA | 1.00E-01 | -2.59E+00 | 0.3718 | 222 | 30.66% | 7771.5 | 28.15% |
| RORgt(NR)/EL4-RORgt.Flag-ChIP-Seq(GSE56019)/Homer | AAYTAGGTCA | 1.00E-01 | -2.57E+00 | 0.3718 | 64 | 8.84% | 2028.9 | 7.35% |
| STAT1(Stat)/HelaS3-STAT1-ChIP-Seq(GSE12782)/Homer | NATTTCCNGGAAAT | 1.00E-01 | -2.57E+00 | 0.3718 | 162 | 22.38% | 5560.7 | 20.14% |
| NeuroD1(bHLH)/Islet-NeuroD1-ChIP-Seq(GSE30298)/Homer | GCCATCTGTT | 1.00E-01 | -2.55E+00 | 0.3718 | 309 | 42.68% | 11042.2 | 39.99% |
| Maz(Zf)/HepG2-Maz-ChIP-Seq(GSE31477)/Homer | GGGGGGGG | 1.00E-01 | -2.54E+00 | 0.3718 | 586 | 80.94% | 21732.2 | 78.70% |
| GATA(Zf),IR3/iTreg-Gata3-ChIP-Seq(GSE20898)/Homer | NNNNNBAGATAWYATCTVHN | 1.00E-01 | -2.52E+00 | 0.3718 | 67 | 9.25% | 2140.8 | 7.75% |
| Foxo1(Forkhead)/RAW-Foxo1-ChIP-Seq(Fan et al.)/Homer | CTGTTTAC | 1.00E-01 | -2.52E+00 | 0.3718 | 566 | 78.18% | 20948.8 | 75.87% |
| EHF(ETS)/LoVo-EHF-ChIP-Seq(GSE49402)/Homer | AVCAGGAAGT | 1.00E-01 | -2.47E+00 | 0.3718 | 493 | 68.09% | 18106.4 | 65.57% |
| Srebp2(bHLH)/HepG2-Srebp2-ChIP-Seq(GSE31477)/Homer | CGGTCACSCCAC | 1.00E-01 | -2.45E+00 | 0.3718 | 71 | 9.81% | 2292.2 | 8.30% |
| Sox4(HMG)/proB-Sox4-ChIP-Seq(GSE50066)/Homer | YCTTTGTTCC | 1.00E-01 | -2.39E+00 | 0.3823 | 345 | 47.65% | 12448.6 | 45.08% |
| IRF1(IRF)/PBMC-IRF1-ChIP-Seq(GSE43036)/Homer | GAAAGTGAAAGT | 1.00E-01 | -2.38E+00 | 0.383 | 74 | 10.22% | 2410.5 | 8.73% |
| EWS:FLI1-fusion(ETS)/SK_N_MC-EWS:FLI1-ChIP-Seq(SRA014231)/Homer | VACAGGAAAT | 1.00E-01 | -2.34E+00 | 0.3922 | 293 | 40.47% | 10497.1 | 38.02% |
| Bcl6(Zf)/Liver-Bcl6-ChIP-Seq(GSE31578)/Homer | NNNCTTTCCAGGAAA | 1.00E-01 | -2.32E+00 | 0.3922 | 493 | 68.09% | 18146.1 | 65.72% |
| GABPA(ETS)/Jurkat-GABPa-ChIP-Seq(GSE17954)/Homer | RACCGGAAGT | 1.00E-01 | -2.32E+00 | 0.3922 | 424 | 58.56% | 15485.3 | 56.08% |
| ETV1(ETS)/GIST48-ETV1-ChIP-Seq(GSE22441)/Homer | AACCGGAAGT | 1.00E+00 | -2.26E+00 | 0.4073 | 526 | 72.65% | 19448.3 | 70.43% |
| Arnt:Ahr(bHLH)/MCF7-Arnt-ChIP-Seq(Lo et al.)/Homer | TBGCACGCAA | 1.00E+00 | -2.22E+00 | 0.4161 | 294 | 40.61% | 10569.3 | 38.28% |
| Chop(bZIP)/MEF-Chop-ChIP-Seq(GSE35681)/Homer | ATTGCATCAT | 1.00E+00 | -2.19E+00 | 0.4208 | 88 | 12.15% | 2943.7 | 10.66% |
| Cdx2(Homeobox)/mES-Cdx2-ChIP-Seq(GSE14586)/Homer | GYMATAAAAH | 1.00E+00 | -2.18E+00 | 0.4208 | 291 | 40.19% | 10466.7 | 37.91% |
| Tcf3(HMG)/mES-Tcf3-ChIP-Seq(GSE11724)/Homer | ASWTCAAAGG | 1.00E+00 | -2.16E+00 | 0.4235 | 128 | 17.68% | 4404.9 | 15.95% |
| Smad2(MAD)/ES-SMAD2-ChIP-Seq(GSE29422)/Homer | CTGTCTGG | 1.00E+00 | -2.15E+00 | 0.4235 | 516 | 71.27% | 19089.3 | 69.13% |
| Reverb(NR),DR2/RAW-Reverba.biotin-ChIP-Seq(GSE45914)/Homer | GTRGGTCASTGGGTCA | 1.00E+00 | -2.13E+00 | 0.4254 | 65 | 8.98% | 2130.3 | 7.72% |
| Ap4(bHLH)/AML-Tfap4-ChIP-Seq(GSE45738)/Homer | NAHCAGCTGD | 1.00E+00 | -2.09E+00 | 0.4336 | 406 | 56.08% | 14864.1 | 53.83% |
| RUNX(Runt)/HPC7-Runx1-ChIP-Seq(GSE22178)/Homer | SAAACCACAG | 1.00E+00 | -2.09E+00 | 0.4336 | 291 | 40.19% | 10494.9 | 38.01% |
| Tcf4(HMG)/Hct116-Tcf4-ChIP-Seq(SRA012054)/Homer | ASATCAAAGGVA | 1.00E+00 | -2.09E+00 | 0.4336 | 211 | 29.14% | 7493.8 | 27.14% |
| Stat3+il21(Stat)/CD4-Stat3-ChIP-Seq(GSE19198)/Homer | SVYTTCCNGGAARB | 1.00E+00 | -2.08E+00 | 0.4336 | 321 | 44.34% | 11633.9 | 42.13% |
| NF1(CTF)/LNCAP-NF1-ChIP-Seq(Unpublished)/Homer | CYTGGCABNSTGCCAR | 1.00E+00 | -2.04E+00 | 0.4336 | 177 | 24.45% | 6239.4 | 22.60% |
| HRE(HSF)/HepG2-HSF1-ChIP-Seq(GSE31477)/Homer | BSTTCTRGAABVTTCYAGAA | 1.00E+00 | -2.02E+00 | 0.4388 | 86 | 11.88% | 2904.8 | 10.52% |
| HIF2a(bHLH)/785_O-HIF2a-ChIP-Seq(GSE34871)/Homer | GCACGTACCC | 1.00E+00 | -1.99E+00 | 0.4458 | 173 | 23.90% | 6105.6 | 22.11% |
| Esrrb(NR)/mES-Esrrb-ChIP-Seq(GSE11431)/Homer | KTGACCTTGA | 1.00E+00 | -1.94E+00 | 0.4648 | 274 | 37.85% | 9901.8 | 35.86% |
| Ptf1a(bHLH)/Panc1-Ptf1a-ChIP-Seq(GSE47459)/Homer | ACAGCTGTTN | 1.00E+00 | -1.93E+00 | 0.4648 | 622 | 85.91% | 23304.2 | 84.40% |
| TCFL2(HMG)/K562-TCF7L2-ChIP-Seq(GSE29196)/Homer | ACWTCAAAGG | 1.00E+00 | -1.92E+00 | 0.4648 | 46 | 6.35% | 1488 | 5.39% |
| Atf4(bZIP)/MEF-Atf4-ChIP-Seq(GSE35681)/Homer | MTGATGCAAT | 1.00E+00 | -1.86E+00 | 0.4824 | 112 | 15.47% | 3885.6 | 14.07% |
| Atoh1(bHLH)/Cerebellum-Atoh1-ChIP-Seq(GSE22111)/Homer | VNRVCAGCTGGY | 1.00E+00 | -1.85E+00 | 0.4839 | 380 | 52.49% | 13951.7 | 50.53% |
| Foxh1(Forkhead)/hESC-FOXH1-ChIP-Seq(GSE29422)/Homer | NNTGTGGATTSS | 1.00E+00 | -1.77E+00 | 0.5164 | 258 | 35.64% | 9351.8 | 33.87% |
| Jun-AP1(bZIP)/K562-cJun-ChIP-Seq(GSE31477)/Homer | GATGASTCATCN | 1.00E+00 | -1.73E+00 | 0.5295 | 88 | 12.15% | 3035.7 | 10.99% |
| Bach2(bZIP)/OCILy7-Bach2-ChIP-Seq(GSE44420)/Homer | TGCTGAGTCA | 1.00E+00 | -1.72E+00 | 0.5316 | 77 | 10.64% | 2637.9 | 9.55% |
| RUNX1(Runt)/Jurkat-RUNX1-ChIP-Seq(GSE29180)/Homer | AAACCACARM | 1.00E+00 | -1.69E+00 | 0.5435 | 383 | 52.90% | 14122.3 | 51.14% |
| AR-halfsite(NR)/LNCaP-AR-ChIP-Seq(GSE27824)/Homer | CCAGGAACAG | 1.00E+00 | -1.69E+00 | 0.5435 | 692 | 95.58% | 26164.1 | 94.76% |
| Smad4(MAD)/ESC-SMAD4-ChIP-Seq(GSE29422)/Homer | VBSYGTCTGG | 1.00E+00 | -1.68E+00 | 0.5435 | 523 | 72.24% | 19503.3 | 70.63% |
| NFkB-p50,p52(RHD)/Monocyte-p50-ChIP-Chip(Schreiber et al.)/Homer | GGGGGAATCCCC | 1.00E+00 | -1.67E+00 | 0.5435 | 65 | 8.98% | 2210.5 | 8.01% |
| Fli1(ETS)/CD8-FLI-ChIP-Seq(GSE20898)/Homer | NRYTTCCGGH | 1.00E+00 | -1.63E+00 | 0.5525 | 485 | 66.99% | 18055.7 | 65.39% |
| Tcf12(bHLH)/GM12878-Tcf12-ChIP-Seq(GSE32465)/Homer | VCAGCTGYTG | 1.00E+00 | -1.61E+00 | 0.5555 | 347 | 47.93% | 12778.9 | 46.28% |
| GRHL2(CP2)/HBE-GRHL2-ChIP-Seq(GSE46194)/Homer | AAACYKGTTWDACMRGTTTB | 1.00E+00 | -1.60E+00 | 0.5565 | 169 | 23.34% | 6068.4 | 21.98% |
| AP-2gamma(AP2)/MCF7-TFAP2C-ChIP-Seq(GSE21234)/Homer | SCCTSAGGSCAW | 1.00E+00 | -1.60E+00 | 0.5565 | 425 | 58.70% | 15761.2 | 57.08% |
| Lhx3(Homeobox)/Neuron-Lhx3-ChIP-Seq(GSE31456)/Homer | ADBTAATTAR | 1.00E+00 | -1.60E+00 | 0.5565 | 514 | 70.99% | 19185.7 | 69.48% |
| Nur77(NR)/K562-NR4A1-ChIP-Seq(GSE31363)/Homer | TGACCTTTNCNT | 1.00E+00 | -1.57E+00 | 0.5575 | 94 | 12.98% | 3294.7 | 11.93% |
| Unknown(Homeobox)/Limb-p300-ChIP-Seq/Homer | SSCMATWAAA | 1.00E+00 | -1.54E+00 | 0.5674 | 280 | 38.67% | 10263.5 | 37.17% |
| Nrf2(bZIP)/Lymphoblast-Nrf2-ChIP-Seq(GSE37589)/Homer | HTGCTGAGTCAT | 1.00E+00 | -1.49E+00 | 0.5891 | 18 | 2.49% | 559.9 | 2.03% |
| Smad3(MAD)/NPC-Smad3-ChIP-Seq(GSE36673)/Homer | TWGTCTGV | 1.00E+00 | -1.43E+00 | 0.6171 | 661 | 91.30% | 24970.1 | 90.43% |
| NFY(CCAAT)/Promoter/Homer | RGCCAATSRG | 1.00E+00 | -1.38E+00 | 0.6439 | 378 | 52.21% | 14049 | 50.88% |
| Egr2(Zf)/Thymocytes-Egr2-ChIP-Seq(GSE34254)/Homer | NGCGTGGGCGGR | 1.00E+00 | -1.36E+00 | 0.6543 | 124 | 17.13% | 4463.4 | 16.16% |
| MyoG(bHLH)/C2C12-MyoG-ChIP-Seq(GSE36024)/Homer | AACAGCTG | 1.00E+00 | -1.31E+00 | 0.6792 | 376 | 51.93% | 14002.9 | 50.71% |
| TR4(NR),DR1/Hela-TR4-ChIP-Seq(GSE24685)/Homer | GAGGTCAAAGGTCA | 1.00E+00 | -1.30E+00 | 0.6794 | 61 | 8.43% | 2142.1 | 7.76% |
| ERE(NR),IR3/MCF7-ERa-ChIP-Seq(Unpublished)/Homer | VAGGTCACNSTGACC | 1.00E+00 | -1.30E+00 | 0.6794 | 113 | 15.61% | 4070 | 14.74% |
| ETS1(ETS)/Jurkat-ETS1-ChIP-Seq(GSE17954)/Homer | ACAGGAAGTG | 1.00E+00 | -1.29E+00 | 0.6794 | 436 | 60.22% | 16305.2 | 59.05% |
| ISRE(IRF)/ThioMac-LPS-Expression(GSE23622)/Homer | AGTTTCASTTTC | 1.00E+00 | -1.28E+00 | 0.6794 | 30 | 4.14% | 1013.8 | 3.67% |
| STAT4(Stat)/CD4-Stat4-ChIP-Seq(GSE22104)/Homer | NYTTCCWGGAAR | 1.00E+00 | -1.28E+00 | 0.6794 | 388 | 53.59% | 14473 | 52.42% |
| Otx2(Homeobox)/EpiLC-Otx2-ChIP-Seq(GSE56098)/Homer | NYTAATCCYB | 1.00E+00 | -1.27E+00 | 0.6794 | 310 | 42.82% | 11507.3 | 41.67% |
| Pax7(Paired,Homeobox),long/Myoblast-Pax7-ChIP-Seq(GSE25064)/Homer | TAATCHGATTAC | 1.00E+00 | -1.25E+00 | 0.6794 | 12 | 1.66% | 375.1 | 1.36% |
| RBPJ:Ebox(?,bHLH)/Panc1-Rbpj1-ChIP-Seq(GSE47459)/Homer | GGGRAARRGRMCAGMTG | 1.00E+00 | -1.22E+00 | 0.6899 | 147 | 20.30% | 5368.2 | 19.44% |
| Pax7(Paired,Homeobox)/Myoblast-Pax7-ChIP-Seq(GSE25064)/Homer | TAATCAATTA | 1.00E+00 | -1.22E+00 | 0.6899 | 41 | 5.66% | 1425.8 | 5.16% |
| Sox2(HMG)/mES-Sox2-ChIP-Seq(GSE11431)/Homer | BCCATTGTTC | 1.00E+00 | -1.20E+00 | 0.69 | 341 | 47.10% | 12715.6 | 46.05% |
| EBF(EBF)/proBcell-EBF-ChIP-Seq(GSE21978)/Homer | DGTCCCYRGGGA | 1.00E+00 | -1.16E+00 | 0.717 | 113 | 15.61% | 4115 | 14.90% |
| NF-E2(bZIP)/K562-NFE2-ChIP-Seq(GSE31477)/Homer | GATGACTCAGCA | 1.00E+00 | -1.14E+00 | 0.7229 | 20 | 2.76% | 673.8 | 2.44% |
| Pitx1(Homeobox)/Chicken-Pitx1-ChIP-Seq(GSE38910)/Homer | TAATCCCN | 1.00E+00 | -1.14E+00 | 0.7229 | 706 | 97.51% | 26821.8 | 97.14% |
| GFY-Staf(?,Zf)/Promoter/Homer | RACTACAATTCCCAGAAKGC | 1.00E+00 | -1.13E+00 | 0.7229 | 48 | 6.63% | 1700.1 | 6.16% |
| HNF6(Homeobox)/Liver-Hnf6-ChIP-Seq(ERP000394)/Homer | NTATYGATCH | 1.00E+00 | -1.13E+00 | 0.7229 | 217 | 29.97% | 8041.5 | 29.12% |
| Pax8(Paired,Homeobox)/Thyroid-Pax8-ChIP-Seq(GSE26938)/Homer | GTCATGCHTGRCTGS | 1.00E+00 | -1.11E+00 | 0.7229 | 138 | 19.06% | 5068.6 | 18.36% |
| IRF2(IRF)/Erythroblas-IRF2-ChIP-Seq(GSE36985)/Homer | GAAASYGAAASY | 1.00E+00 | -1.11E+00 | 0.7229 | 53 | 7.32% | 1891.4 | 6.85% |
| Nkx2.1(Homeobox)/LungAC-Nkx2.1-ChIP-Seq(GSE43252)/Homer | RSCACTYRAG | 1.00E+00 | -1.11E+00 | 0.7229 | 658 | 90.88% | 24937.2 | 90.31% |
| Sox6(HMG)/Myotubes-Sox6-ChIP-Seq(GSE32627)/Homer | CCATTGTTNY | 1.00E+00 | -1.10E+00 | 0.7229 | 505 | 69.75% | 19030 | 68.92% |
| GFY(?)/Promoter/Homer | ACTACAATTCCC | 1.00E+00 | -1.10E+00 | 0.7229 | 57 | 7.87% | 2042.4 | 7.40% |
| MafF(bZIP)/HepG2-MafF-ChIP-Seq(GSE31477)/Homer | HWWGTCAGCAWWTTT | 1.00E+00 | -1.10E+00 | 0.7229 | 103 | 14.23% | 3760.2 | 13.62% |
| RUNX2(Runt)/PCa-RUNX2-ChIP-Seq(GSE33889)/Homer | NWAACCACADNN | 1.00E+00 | -1.09E+00 | 0.7229 | 312 | 43.09% | 11664.5 | 42.24% |
| PR(NR)/T47D-PR-ChIP-Seq(GSE31130)/Homer | VAGRACAKNCTGTBC | 1.00E+00 | -1.09E+00 | 0.7229 | 591 | 81.63% | 22346.8 | 80.93% |
| BMYB(HTH)/Hela-BMYB-ChIP-Seq(GSE27030)/Homer | NHAACBGYYV | 1.00E+00 | -1.09E+00 | 0.7229 | 520 | 71.82% | 19613.7 | 71.03% |
| PAX5(Paired,Homeobox)/GM12878-PAX5-ChIP-Seq(GSE32465)/Homer | GCAGCCAAGCRTGACH | 1.00E+00 | -1.06E+00 | 0.7229 | 165 | 22.79% | 6104.9 | 22.11% |
| p63(p53)/Keratinocyte-p63-ChIP-Seq(GSE17611)/Homer | NNDRCATGYCYNRRCATGYH | 1.00E+00 | -1.06E+00 | 0.7229 | 143 | 19.75% | 5276.4 | 19.11% |
| RUNX-AML(Runt)/CD4+-PolII-ChIP-Seq(Barski et al.)/Homer | GCTGTGGTTW | 1.00E+00 | -1.05E+00 | 0.7229 | 277 | 38.26% | 10351.5 | 37.49% |
| Nkx3.1(Homeobox)/LNCaP-Nkx3.1-ChIP-Seq(GSE28264)/Homer | AAGCACTTAA | 1.00E+00 | -1.03E+00 | 0.7229 | 612 | 84.53% | 23178.4 | 83.94% |
| Nkx2.5(Homeobox)/HL1-Nkx2.5.biotin-ChIP-Seq(GSE21529)/Homer | RRSCACTYAA | 1.00E+00 | -1.02E+00 | 0.7229 | 608 | 83.98% | 23026.9 | 83.39% |
| STAT5(Stat)/mCD4+-Stat5-ChIP-Seq(GSE12346)/Homer | RTTTCTNAGAAA | 1.00E+00 | -9.96E-01 | 0.7229 | 171 | 23.62% | 6359.5 | 23.03% |
| Foxa2(Forkhead)/Liver-Foxa2-ChIP-Seq(GSE25694)/Homer | CYTGTTTACWYW | 1.00E+00 | -9.65E-01 | 0.7395 | 318 | 43.92% | 11953.9 | 43.29% |
| EBF1(EBF)/Near-E2A-ChIP-Seq(GSE21512)/Homer | GTCCCCWGGGGA | 1.00E+00 | -9.47E-01 | 0.7476 | 407 | 56.22% | 15355.5 | 55.61% |
| E2A(bHLH)/proBcell-E2A-ChIP-Seq(GSE21978)/Homer | DNRCAGCTGY | 1.00E+00 | -9.45E-01 | 0.7476 | 457 | 63.12% | 17266.2 | 62.53% |
| Oct4(POU,Homeobox)/mES-Oct4-ChIP-Seq(GSE11431)/Homer | ATTTGCATAW | 1.00E+00 | -9.44E-01 | 0.7476 | 183 | 25.28% | 6836.6 | 24.76% |
| PU.1-IRF(ETS:IRF)/Bcell-PU.1-ChIP-Seq(GSE21512)/Homer | MGGAAGTGAAAC | 1.00E+00 | -9.40E-01 | 0.7476 | 476 | 65.75% | 17995.9 | 65.17% |
| Mef2c(MADS)/GM12878-Mef2c-ChIP-Seq(GSE32465)/Homer | DCYAAAAATAGM | 1.00E+00 | -9.34E-01 | 0.7476 | 181 | 25.00% | 6765.7 | 24.50% |
| TEAD4(TEA)/Tropoblast-Tead4-ChIP-Seq(GSE37350)/Homer | CCWGGAATGY | 1.00E+00 | -9.03E-01 | 0.7537 | 333 | 45.99% | 12557.5 | 45.48% |
| GRE(NR),IR3/A549-GR-ChIP-Seq(GSE32465)/Homer | NRGVACABNVTGTYCY | 1.00E+00 | -8.94E-01 | 0.7553 | 57 | 7.87% | 2096.2 | 7.59% |
| GATA:SCL(Zf,bHLH)/Ter119-SCL-ChIP-Seq(GSE18720)/Homer | CRGCTGBNGNSNNSAGATAA | 1.00E+00 | -8.88E-01 | 0.7553 | 56 | 7.73% | 2060.9 | 7.46% |
| Erra(NR)/HepG2-Erra-ChIP-Seq(GSE31477)/Homer | CAAAGGTCAG | 1.00E+00 | -8.86E-01 | 0.7553 | 582 | 80.39% | 22081.3 | 79.97% |
| ELF1(ETS)/Jurkat-ELF1-ChIP-Seq(SRA014231)/Homer | AVCCGGAAGT | 1.00E+00 | -8.82E-01 | 0.7553 | 303 | 41.85% | 11426.4 | 41.38% |
| Nanog(Homeobox)/mES-Nanog-ChIP-Seq(GSE11724)/Homer | RGCCATTAAC | 1.00E+00 | -8.60E-01 | 0.7602 | 703 | 97.10% | 26751.7 | 96.88% |
| p53(p53)/Saos-p53-ChIP-Seq(GSE15780)/Homer | RRCATGYCYRGRCATGYYYN | 1.00E+00 | -8.54E-01 | 0.7602 | 39 | 5.39% | 1430.9 | 5.18% |
| p53(p53)/Saos-p53-ChIP-Seq/Homer | RRCATGYCYRGRCATGYYYN | 1.00E+00 | -8.54E-01 | 0.7602 | 39 | 5.39% | 1430.9 | 5.18% |
| ZNF143\|STAF(Zf)/CUTLL-ZNF143-ChIP-Seq(GSE29600)/Homer | ATTTCCCAGVAKSCY | 1.00E+00 | -8.52E-01 | 0.7602 | 175 | 24.17% | 6576.3 | 23.82% |
| EKLF(Zf)/Erythrocyte-Klf1-ChIP-Seq(GSE20478)/Homer | NWGGGTGTGGCY | 1.00E+00 | -8.33E-01 | 0.7602 | 118 | 16.30% | 4422.6 | 16.02% |
| MafA(bZIP)/Islet-MafA-ChIP-Seq(GSE30298)/Homer | TGCTGACTCA | 1.00E+00 | -8.30E-01 | 0.7602 | 289 | 39.92% | 10922.7 | 39.56% |
| TEAD(TEA)/Fibroblast-PU.1-ChIP-Seq(Unpublished)/Homer | YCWGGAATGY | 1.00E+00 | -8.09E-01 | 0.7684 | 265 | 36.60% | 10021 | 36.29% |
| Tbx20(T-box)/Heart-Tbx20-ChIP-Seq(GSE29636)/Homer | GGTGYTGACAGS | 1.00E+00 | -8.08E-01 | 0.7684 | 99 | 13.67% | 3712.1 | 13.44% |
| ZFX(Zf)/mES-Zfx-ChIP-Seq(GSE11431)/Homer | AGGCCTRG | 1.00E+00 | -7.92E-01 | 0.7714 | 435 | 60.08% | 16509.4 | 59.79% |
| TEAD2(TEA)/Py2T-Tead2-ChIP-Seq(GSE55709)/Homer | CCWGGAATGY | 1.00E+00 | -7.84E-01 | 0.7724 | 219 | 30.25% | 8283.3 | 30.00% |
| Tbx5(T-box)/HL1-Tbx5.biotin-ChIP-Seq(GSE21529)/Homer | AGGTGTCA | 1.00E+00 | -7.78E-01 | 0.7727 | 668 | 92.27% | 25423.6 | 92.07% |
| GATA(Zf),IR4/iTreg-Gata3-ChIP-Seq(GSE20898)/Homer | NAGATWNBNATCTNN | 1.00E+00 | -7.67E-01 | 0.7763 | 37 | 5.11% | 1377 | 4.99% |
| Sox3(HMG)/NPC-Sox3-ChIP-Seq(GSE33059)/Homer | CCWTTGTY | 1.00E+00 | -7.39E-01 | 0.7932 | 530 | 73.20% | 20165.7 | 73.03% |
| Tbet(T-box)/CD8-Tbet-ChIP-Seq(GSE33802)/Homer | AGGTGTGAAM | 1.00E+00 | -7.35E-01 | 0.7932 | 387 | 53.45% | 14713.3 | 53.29% |
| Nr5a2(NR)/Pancreas-LRH1-ChIP-Seq(GSE34295)/Homer | BTCAAGGTCA | 1.00E+00 | -7.17E-01 | 0.8007 | 283 | 39.09% | 10760.1 | 38.97% |
| THRa(NR)/C17.2-THRa-ChIP-Seq(GSE38347)/Homer | GGTCANYTGAGGWCA | 1.00E+00 | -7.17E-01 | 0.8007 | 177 | 24.45% | 6721.4 | 24.34% |
| Tlx?(NR)/NPC-H3K4me1-ChIP-Seq(GSE16256)/Homer | CTGGCAGSCTGCCA | 1.00E+00 | -7.16E-01 | 0.8007 | 174 | 24.03% | 6607.6 | 23.93% |
| FOXA1:AR(Forkhead,NR)/LNCAP-AR-ChIP-Seq(GSE27824)/Homer | AGTAAACAAAAAAGAACAND | 1.00E+00 | -7.02E-01 | 0.8007 | 30 | 4.14% | 1128.7 | 4.09% |
| ARE(NR)/LNCAP-AR-ChIP-Seq(GSE27824)/Homer | RGRACASNSTGTYCYB | 1.00E+00 | -7.02E-01 | 0.8007 | 102 | 14.09% | 3872 | 14.02% |
| MyoD(bHLH)/Myotube-MyoD-ChIP-Seq(GSE21614)/Homer | RRCAGCTGYTSY | 1.00E+00 | -6.92E-01 | 0.8007 | 279 | 38.54% | 10623.2 | 38.47% |
| CEBP(bZIP)/ThioMac-CEBPb-ChIP-Seq(GSE21512)/Homer | ATTGCGCAAC | 1.00E+00 | -6.87E-01 | 0.8007 | 262 | 36.19% | 9978.9 | 36.14% |
| AP-2alpha(AP2)/Hela-AP2alpha-ChIP-Seq(GSE31477)/Homer | ATGCCCTGAGGC | 1.00E+00 | -6.85E-01 | 0.8007 | 341 | 47.10% | 12991.8 | 47.05% |
| Egr1(Zf)/K562-Egr1-ChIP-Seq(GSE32465)/Homer | TGCGTGGGYG | 1.00E+00 | -6.78E-01 | 0.8007 | 350 | 48.34% | 13339.7 | 48.31% |
| Tbox:Smad(T-box,MAD)/ESCd5-Smad2_3-ChIP-Seq(GSE29422)/Homer | AGGTGHCAGACA | 1.00E+00 | -6.71E-01 | 0.8007 | 83 | 11.46% | 3160.1 | 11.44% |
| NRF(NRF)/Promoter/Homer | STGCGCATGCGC | 1.00E+00 | -6.61E-01 | 0.8007 | 132 | 18.23% | 5035.2 | 18.24% |
| Bach1(bZIP)/K562-Bach1-ChIP-Seq(GSE31477)/Homer | AWWNTGCTGAGTCAT | 1.00E+00 | -6.35E-01 | 0.8136 | 18 | 2.49% | 685.3 | 2.48% |
| Pbx3(Homeobox)/GM12878-PBX3-ChIP-Seq(GSE32465)/Homer | SCTGTCAMTCAN | 1.00E+00 | -6.27E-01 | 0.8153 | 114 | 15.75% | 4365.9 | 15.81% |
| FOXP1(Forkhead)/H9-FOXP1-ChIP-Seq(GSE31006)/Homer | NYYTGTTTACHN | 1.00E+00 | -6.22E-01 | 0.8153 | 190 | 26.24% | 7272.7 | 26.34% |
| PAX5(Paired,Homeobox),condensed/GM12878-PAX5-ChIP-Seq(GSE32465)/Homer | GTCACGCTCSCTGM | 1.00E+00 | -6.16E-01 | 0.8153 | 44 | 6.08% | 1689.4 | 6.12% |
| TATA-Box(TBP)/Promoter/Homer | CCTTTTAWAGSC | 1.00E+00 | -6.02E-01 | 0.8213 | 491 | 67.82% | 18762.3 | 67.95% |
| Lhx2(Homeobox)/HFSC-Lhx2-ChIP-Seq(GSE48068)/Homer | TAATTAGN | 1.00E+00 | -5.97E-01 | 0.8213 | 370 | 51.10% | 14157.3 | 51.27% |
| GSC(Homeobox)/FrogEmbryos-GSC-ChIP-Seq(DRA000576)/Homer | RGGATTAR | 1.00E+00 | -5.94E-01 | 0.8213 | 403 | 55.66% | 15417.8 | 55.84% |
| Gata2(Zf)/K562-GATA2-ChIP-Seq(GSE18829)/Homer | BBCTTATCTS | 1.00E+00 | -5.89E-01 | 0.8213 | 261 | 36.05% | 10005.8 | 36.24% |
| Elk1(ETS)/Hela-Elk1-ChIP-Seq(GSE31477)/Homer | HACTTCCGGY | 1.00E+00 | -5.85E-01 | 0.8213 | 322 | 44.48% | 12336.7 | 44.68% |
| ETS(ETS)/Promoter/Homer | AACCGGAAGT | 1.00E+00 | -5.82E-01 | 0.8213 | 207 | 28.59% | 7947.9 | 28.78% |
| Myf5(bHLH)/GM-Myf5-ChIP-Seq(GSE24852)/Homer | BAACAGCTGT | 1.00E+00 | -5.68E-01 | 0.8217 | 262 | 36.19% | 10058.1 | 36.43% |
| NF1-halfsite(CTF)/LNCaP-NF1-ChIP-Seq(Unpublished)/Homer | YTGCCAAG | 1.00E+00 | -5.59E-01 | 0.8248 | 517 | 71.41% | 19780.9 | 71.64% |
| PRDM14(Zf)/H1-PRDM14-ChIP-Seq(GSE22767)/Homer | RGGTCTCTAACY | 1.00E+00 | -5.56E-01 | 0.8248 | 156 | 21.55% | 6013.3 | 21.78% |
| Oct2(POU,Homeobox)/Bcell-Oct2-ChIP-Seq(GSE21512)/Homer | ATATGCAAAT | 1.00E+00 | -5.49E-01 | 0.8248 | 117 | 16.16% | 4522.3 | 16.38% |
| ETS:E-box(ETS,bHLH)/HPC7-Scl-ChIP-Seq(GSE22178)/Homer | AGGAARCAGCTG | 1.00E+00 | -5.08E-01 | 0.854 | 41 | 5.66% | 1612.6 | 5.84% |
| Nr5a2(NR)/mES-Nr5a2-ChIP-Seq(GSE19019)/Homer | BTCAAGGTCA | 1.00E+00 | -4.93E-01 | 0.8622 | 220 | 30.39% | 8508.5 | 30.81% |
| Elk4(ETS)/Hela-Elk4-ChIP-Seq(GSE31477)/Homer | NRYTTCCGGY | 1.00E+00 | -4.88E-01 | 0.8624 | 325 | 44.89% | 12526.3 | 45.37% |
| Oct4:Sox17(POU,Homeobox,HMG)/F9-Sox17-ChIP-Seq(GSE44553)/Homer | CCATTGTATGCAAAT | 1.00E+00 | -4.82E-01 | 0.8624 | 56 | 7.73% | 2205.1 | 7.99% |
| ERG(ETS)/VCaP-ERG-ChIP-Seq(GSE14097)/Homer | ACAGGAAGTG | 1.00E+00 | -4.73E-01 | 0.8662 | 538 | 74.31% | 20638.2 | 74.74% |
| RARg(NR)/ES-RARg-ChIP-Seq(GSE30538)/Homer | AGGTCAAGGTCA | 1.00E+00 | -4.60E-01 | 0.8723 | 14 | 1.93% | 569.5 | 2.06% |
| Hoxc9(Homeobox)/Ainv15-Hoxc9-ChIP-Seq(GSE21812)/Homer | GGCCATAAATCA | 1.00E+00 | -4.42E-01 | 0.8834 | 197 | 27.21% | 7666.6 | 27.77% |
| REST-NRSF(Zf)/Jurkat-NRSF-ChIP-Seq/Homer | GGMGCTGTCCATGGTGCTGA | 1.00E+00 | -4.41E-01 | 0.8834 | 4 | 0.55% | 168 | 0.61% |
| T1ISRE(IRF)/ThioMac-Ifnb-Expression/Homer | ACTTTCGTTTCT | 1.00E+00 | -4.38E-01 | 0.8834 | 5 | 0.69% | 210.1 | 0.76% |
| E2F(E2F)/Hela-CellCycle-Expression/Homer | TTSGCGCGAAAA | 1.00E+00 | -4.26E-01 | 0.8843 | 35 | 4.83% | 1410 | 5.11% |
| Atf1(bZIP)/K562-ATF1-ChIP-Seq(GSE31477)/Homer | GATGACGTCA | 1.00E+00 | -4.20E-01 | 0.8846 | 257 | 35.50% | 9985.4 | 36.16% |
| Phox2a(Homeobox)/Neuron-Phox2a-ChIP-Seq(GSE31456)/Homer | YTAATYNRATTA | 1.00E+00 | -4.02E-01 | 0.8962 | 152 | 20.99% | 5967.6 | 21.61% |
| Gata4(Zf)/Heart-Gata4-ChIP-Seq(GSE35151)/Homer | NBWGATAAGR | 1.00E+00 | -3.97E-01 | 0.8967 | 365 | 50.41% | 14133.6 | 51.19% |
| IRF4(IRF)/GM12878-IRF4-ChIP-Seq(GSE32465)/Homer | ACTGAAACCA | 1.00E+00 | -3.89E-01 | 0.8993 | 178 | 24.59% | 6981 | 25.28% |
| EBNA1(EBV virus)/Raji-EBNA1-ChIP-Seq(GSE30709)/Homer | GGYAGCAYDTGCTDCCCNNN | 1.00E+00 | -3.85E-01 | 0.8993 | 6 | 0.83% | 261.3 | 0.95% |
| HOXA9(Homeobox)/HSC-Hoxa9-ChIP-Seq(GSE33509)/Homer | GGCCATAAATCA | 1.00E+00 | -3.75E-01 | 0.9031 | 249 | 34.39% | 9721.6 | 35.21% |
| Fox:Ebox(Forkhead,bHLH)/Panc1-Foxa2-ChIP-Seq(GSE47459)/Homer | NNNVCTGWGYAAACASN | 1.00E+00 | -3.74E-01 | 0.9031 | 363 | 50.14% | 14080 | 50.99% |
| CEBP:CEBP(bZIP)/MEF-Chop-ChIP-Seq(GSE35681)/Homer | NTNATGCAAYMNNHTGMAAY | 1.00E+00 | -3.70E-01 | 0.9031 | 53 | 7.32% | 2144.6 | 7.77% |
| Mef2a(MADS)/HL1-Mef2a.biotin-ChIP-Seq(GSE21529)/Homer | CYAAAAATAG | 1.00E+00 | -3.68E-01 | 0.9031 | 169 | 23.34% | 6651.1 | 24.09% |
| FXR(NR),IR1/Liver-FXR-ChIP-Seq(Chong et al.)/Homer | AGGTCANTGACCTB | 1.00E+00 | -3.68E-01 | 0.9031 | 162 | 22.38% | 6381.8 | 23.11% |
| Mouse_Recombination_Hotspot(Zf)/Testis-DMC1-ChIP-Seq(GSE24438)/Homer | ACTYKNATTCGTGNTACTTC | 1.00E+00 | -3.54E-01 | 0.9031 | 27 | 3.73% | 1123.9 | 4.07% |
| HOXA2(Homeobox)/mES-Hoxa2-ChIP-Seq(Donaldson et al.)/Homer | GYCATCMATCAT | 1.00E+00 | -3.51E-01 | 0.9031 | 36 | 4.97% | 1483.7 | 5.37% |
| LXRE(NR),DR4/RAW-LXRb.biotin-ChIP-Seq(GSE21512)/Homer | RGGTTACTANAGGTCA | 1.00E+00 | -3.50E-01 | 0.9031 | 19 | 2.62% | 804 | 2.91% |
| NFkB-p65(RHD)/GM12787-p65-ChIP-Seq(GSE19485)/Homer | WGGGGATTTCCC | 1.00E+00 | -3.42E-01 | 0.9031 | 216 | 29.83% | 8486.6 | 30.73% |
| c-Jun-CRE(bZIP)/K562-cJun-ChIP-Seq(GSE31477)/Homer | ATGACGTCATCY | 1.00E+00 | -3.41E-01 | 0.9031 | 123 | 16.99% | 4895.8 | 17.73% |
| STAT6(Stat)/Macrophage-Stat6-ChIP-Seq(GSE38377)/Homer | TTCCKNAGAA | 1.00E+00 | -3.40E-01 | 0.9031 | 231 | 31.91% | 9064.6 | 32.83% |
| Gata1(Zf)/K562-GATA1-ChIP-Seq(GSE18829)/Homer | SAGATAAGRV | 1.00E+00 | -3.40E-01 | 0.9031 | 232 | 32.04% | 9103.5 | 32.97% |
| CArG(MADS)/PUER-Srf-ChIP-Seq(Sullivan et al.)/Homer | CCATATATGGNM | 1.00E+00 | -3.30E-01 | 0.9031 | 140 | 19.34% | 5564.7 | 20.15% |
| GATA3(Zf),DR4/iTreg-Gata3-ChIP-Seq(GSE20898)/Homer | AGATGKDGAGATAAG | 1.00E+00 | -3.21E-01 | 0.9031 | 31 | 4.28% | 1298.2 | 4.70% |
| VDR(NR),DR3/GM10855-VDR+vitD-ChIP-Seq(GSE22484)/Homer | ARAGGTCANWGAGTTCANNN | 1.00E+00 | -3.15E-01 | 0.9031 | 87 | 12.02% | 3513.4 | 12.72% |
| FOXA1(Forkhead)/LNCAP-FOXA1-ChIP-Seq(GSE27824)/Homer | WAAGTAAACA | 1.00E+00 | -3.05E-01 | 0.9031 | 419 | 57.87% | 16284.3 | 58.97% |
| Sox10(HMG)/SciaticNerve-Sox3-ChIP-Seq(GSE35132)/Homer | CCWTTGTYYB | 1.00E+00 | -3.02E-01 | 0.9031 | 494 | 68.23% | 19127.4 | 69.27% |
| Meis1(Homeobox)/MastCells-Meis1-ChIP-Seq(GSE48085)/Homer | VGCTGWCAVB | 1.00E+00 | -2.93E-01 | 0.9033 | 510 | 70.44% | 19741 | 71.49% |
| Pit1+1bp(Homeobox)/GCrat-Pit1-ChIP-Seq(GSE58009)/Homer | ATGCATAATTCA | 1.00E+00 | -2.88E-01 | 0.9035 | 131 | 18.09% | 5253.6 | 19.03% |
| GRE(NR),IR3/RAW264.7-GRE-ChIP-Seq(Unpublished)/Homer | VAGRACAKWCTGTYC | 1.00E+00 | -2.88E-01 | 0.9035 | 93 | 12.85% | 3770.4 | 13.65% |
| ZNF711(Zf)/SHSY5Y-ZNF711-ChIP-Seq(GSE20673)/Homer | AGGCCTAG | 1.00E+00 | -2.87E-01 | 0.9035 | 526 | 72.65% | 20349.2 | 73.70% |
| E2A(bHLH),near_PU.1/Bcell-PU.1-ChIP-Seq(GSE21512)/Homer | NVCACCTGBN | 1.00E+00 | -2.75E-01 | 0.9035 | 454 | 62.71% | 17646.8 | 63.91% |
| Six1(Homeobox)/Myoblast-Six1-ChIP-Chip(GSE20150)/Homer | GKVTCADRTTWC | 1.00E+00 | -2.67E-01 | 0.9063 | 101 | 13.95% | 4102.1 | 14.86% |
| Pit1(Homeobox)/GCrat-Pit1-ChIP-Seq(GSE58009)/Homer | ATGMATATDC | 1.00E+00 | -2.40E-01 | 0.9271 | 338 | 46.69% | 13284.7 | 48.11% |
| X-box(HTH)/NPC-H3K4me1-ChIP-Seq(GSE16256)/Homer | GGTTGCCATGGCAA | 1.00E+00 | -2.33E-01 | 0.9296 | 52 | 7.18% | 2194.9 | 7.95% |
| Eomes(T-box)/H9-Eomes-ChIP-Seq(GSE26097)/Homer | ATTAACACCT | 1.00E+00 | -2.29E-01 | 0.9296 | 571 | 78.87% | 22099.7 | 80.04% |
| NFAT(RHD)/Jurkat-NFATC1-ChIP-Seq(Jolma et al.)/Homer | ATTTTCCATT | 1.00E+00 | -2.24E-01 | 0.9297 | 331 | 45.72% | 13040.2 | 47.23% |
| Hnf1(Homeobox)/Liver-Foxa2-Chip-Seq(GSE25694)/Homer | GGTTAAWCATTAA | 1.00E+00 | -2.21E-01 | 0.9297 | 60 | 8.29% | 2524.5 | 9.14% |
| Atf2(bZIP)/3T3L1-Atf2-ChIP-Seq(GSE56872)/Homer | NRRTGACGTCAT | 1.00E+00 | -2.20E-01 | 0.9297 | 135 | 18.65% | 5483.4 | 19.86% |
| PAX3:FKHR-fusion(Paired,Homeobox)/Rh4-PAX3:FKHR-ChIP-Seq(GSE19063)/Homer | ACCRTGACTAATTNN | 1.00E+00 | -2.13E-01 | 0.9297 | 76 | 10.50% | 3168.9 | 11.48% |
| YY1(Zf)/Promoter/Homer | CAAGATGGCGGC | 1.00E+00 | -2.07E-01 | 0.9297 | 31 | 4.28% | 1364.5 | 4.94% |
| NFAT:AP1(RHD,bZIP)/Jurkat-NFATC1-ChIP-Seq(Jolma et al.)/Homer | SARTGGAAAAWRTGAGTCAB | 1.00E+00 | -1.97E-01 | 0.9346 | 67 | 9.25% | 2826.6 | 10.24% |
| GATA3(Zf)/iTreg-Gata3-ChIP-Seq(GSE20898)/Homer | AGATAASR | 1.00E+00 | -1.84E-01 | 0.9426 | 473 | 65.33% | 18490.2 | 66.96% |
| CHR(?)/Hela-CellCycle-Expression/Homer | SRGTTTCAAA | 1.00E+00 | -1.80E-01 | 0.9428 | 268 | 37.02% | 10696.6 | 38.74% |
| Hoxb4(Homeobox)/ES-Hoxb4-ChIP-Seq(GSE34014)/Homer | TGATTRATGGCY | 1.00E+00 | -1.73E-01 | 0.9448 | 66 | 9.12% | 2811.9 | 10.18% |
| GATA3(Zf),DR8/iTreg-Gata3-ChIP-Seq(GSE20898)/Homer | AGATSTNDNNDSAGATAASN | 1.00E+00 | -1.69E-01 | 0.9448 | 24 | 3.31% | 1101.5 | 3.99% |
| bZIP:IRF(bZIP,IRF)/Th17-BatF-ChIP-Seq(GSE39756)/Homer | NAGTTTCABTHTGACTNW | 1.00E+00 | -1.67E-01 | 0.9448 | 168 | 23.20% | 6846.4 | 24.79% |
| FOXA1(Forkhead)/MCF7-FOXA1-ChIP-Seq(GSE26831)/Homer | WAAGTAAACA | 1.00E+00 | -1.45E-01 | 0.96 | 357 | 49.31% | 14170.3 | 51.32% |
| E2F1(E2F)/Hela-E2F1-ChIP-Seq(GSE22478)/Homer | CWGGCGGGAA | 1.00E+00 | -1.42E-01 | 0.96 | 131 | 18.09% | 5437.4 | 19.69% |
| PBX1(Homeobox)/MCF7-PBX1-ChIP-Seq(GSE28007)/Homer | GSCTGTCACTCA | 1.00E+00 | -1.34E-01 | 0.9626 | 36 | 4.97% | 1633.7 | 5.92% |
| GLI3(Zf)/Limb-GLI3-ChIP-Chip(GSE11077)/Homer | CGTGGGTGGTCC | 1.00E+00 | -1.32E-01 | 0.9626 | 51 | 7.04% | 2252.8 | 8.16% |
| Pax7(Paired,Homeobox),longest/Myoblast-Pax7-ChIP-Seq(GSE25064)/Homer | NTAATTDGCYAATTANNWWD | 1.00E+00 | -1.26E-01 | 0.9626 | 8 | 1.10% | 435.8 | 1.58% |
| JunD(bZIP)/K562-JunD-ChIP-Seq/Homer | ATGACGTCATCN | 1.00E+00 | -1.22E-01 | 0.9626 | 43 | 5.94% | 1936.4 | 7.01% |
| Gfi1b(Zf)/HPC7-Gfi1b-ChIP-Seq(GSE22178)/Homer | MAATCACTGC | 1.00E+00 | -1.18E-01 | 0.9626 | 233 | 32.18% | 9468 | 34.29% |
| Atf7(bZIP)/3T3L1-Atf7-ChIP-Seq(GSE56872)/Homer | NGRTGACGTCAY | 1.00E+00 | -1.14E-01 | 0.9626 | 179 | 24.72% | 7375.1 | 26.71% |
| CTCF-SatelliteElement(Zf?)/CD4+-CTCF-ChIP-Seq(Barski et al.)/Homer | TGCAGTTCCMVNWRTGGCCA | 1.00E+00 | -1.04E-01 | 0.9676 | 4 | 0.55% | 256.2 | 0.93% |
| Pdx1(Homeobox)/Islet-Pdx1-ChIP-Seq(SRA008281)/Homer | YCATYAATCA | 1.00E+00 | -9.85E-02 | 0.9686 | 315 | 43.51% | 12676.2 | 45.91% |
| NRF1(NRF)/MCF7-NRF1-ChIP-Seq(Unpublished)/Homer | CTGCGCATGCGC | 1.00E+00 | -8.96E-02 | 0.9733 | 105 | 14.50% | 4506.5 | 16.32% |
| OCT4-SOX2-TCF-NANOG(POU,Homeobox,HMG)/mES-Oct4-ChIP-Seq(GSE11431)/Homer | ATTTGCATAACAATG | 1.00E+00 | -8.52E-02 | 0.9737 | 64 | 8.84% | 2857.7 | 10.35% |
| Unknown-ESC-element(?)/mES-Nanog-ChIP-Seq(GSE11724)/Homer | CACAGCAGGGGG | 1.00E+00 | -8.19E-02 | 0.9737 | 208 | 28.73% | 8591.1 | 31.11% |
| E2F7(E2F)/Hela-E2F7-ChIP-Seq(GSE32673)/Homer | VDTTTCCCGCCA | 1.00E+00 | -8.05E-02 | 0.9737 | 70 | 9.67% | 3112 | 11.27% |
| Rfx1(HTH)/NPC-H3K4me1-ChIP-Seq(GSE16256)/Homer | KGTTGCCATGGCAA | 1.00E+00 | -7.89E-02 | 0.9737 | 86 | 11.88% | 3766.1 | 13.64% |
| SPDEF(ETS)/VCaP-SPDEF-ChIP-Seq(SRA014231)/Homer | ASWTCCTGBT | 1.00E+00 | -7.24E-02 | 0.9737 | 393 | 54.28% | 15730.1 | 56.97% |
| STAT6(Stat)/CD4-Stat6-ChIP-Seq(GSE22104)/Homer | ABTTCYYRRGAA | 1.00E+00 | -4.71E-02 | 0.9915 | 213 | 29.42% | 8918 | 32.30% |
| Rfx5(HTH)/GM12878-Rfx5-ChIP-Seq(GSE31477)/Homer | SCCTAGCAACAG | 1.00E+00 | -3.97E-02 | 0.995 | 129 | 17.82% | 5630.6 | 20.39% |
| E2F6(E2F)/Hela-E2F6-ChIP-Seq(GSE31477)/Homer | GGCGGGAARN | 1.00E+00 | -3.03E-02 | 1 | 250 | 34.53% | 10460.6 | 37.88% |
| PRDM9(Zf)/Testis-DMC1-ChIP-Seq(GSE35498)/Homer | ADGGYAGYAGCATCT | 1.00E+00 | -3.03E-02 | 1 | 140 | 19.34% | 6123.9 | 22.18% |
| Brachyury(T-box)/Mesoendoderm-Brachyury-ChIP-exo(GSE54963)/Homer | ANTTMRCASBNNNGTGYKAAN | 1.00E+00 | -2.57E-02 | 1 | 95 | 13.12% | 4328.5 | 15.68% |
| PRDM1(Zf)/Hela-PRDM1-ChIP-Seq(GSE31477)/Homer | ACTTTCACTTTC | 1.00E+00 | -1.07E-02 | 1 | 212 | 29.28% | 9184.6 | 33.26% |
| ZBTB33(Zf)/GM12878-ZBTB33-ChIP-Seq(GSE32465)/Homer | GGVTCTCGCGAGAAC | 1.00E+00 | -4.98E-03 | 1 | 20 | 2.76% | 1267.6 | 4.59% |
| GFX(?)/Promoter/Homer | ATTCTCGCGAGA | 1.00E+00 | -3.68E-03 | 1 | 6 | 0.83% | 557 | 2.02% |
| RFX(HTH)/K562-RFX3-ChIP-Seq(SRA012198)/Homer | CGGTTGCCATGGCAAC | 1.00E+00 | -2.60E-03 | 1 | 32 | 4.42% | 1893.8 | 6.86% |
| Rfx2(HTH)/LoVo-RFX2-ChIP-Seq(GSE49402)/Homer | GTTGCCATGGCAACM | 1.00E+00 | -1.58E-03 | 1 | 35 | 4.83% | 2078.3 | 7.53% |
| E2F4(E2F)/K562-E2F4-ChIP-Seq(GSE31477)/Homer | GGCGGGAAAH | 1.00E+00 | -1.11E-03 | 1 | 214 | 29.56% | 9640 | 34.91% |

**Supplementary Table 5.** Common down motifs.

| **Motif Name** | **Consensus** | **P-value** | **Log P-value** | **q-value (Benjamini)** | **# of Target Sequences with Motif(of 515)** | **% of Target Sequences with Motif** | **# of Background Sequences with Motif(of 27925)** | **% of Background Sequences with Motif** |
| --- | --- | --- | --- | --- | --- | --- | --- | --- |
| Myf5(bHLH)/GM-Myf5-ChIP-Seq(GSE24852)/Homer | BAACAGCTGT | 1.00E-04 | -1.12E+01 | 0.0036 | 238 | 46.21% | 10329.6 | 36.99% |
| Nkx2.1(Homeobox)/LungAC-Nkx2.1-ChIP-Seq(GSE43252)/Homer | RSCACTYRAG | 1.00E-04 | -1.01E+01 | 0.0054 | 489 | 94.95% | 25139.5 | 90.03% |
| MyoD(bHLH)/Myotube-MyoD-ChIP-Seq(GSE21614)/Homer | RRCAGCTGYTSY | 1.00E-03 | -9.08E+00 | 0.01 | 242 | 46.99% | 10841.1 | 38.82% |
| Otx2(Homeobox)/EpiLC-Otx2-ChIP-Seq(GSE56098)/Homer | NYTAATCCYB | 1.00E-03 | -8.24E+00 | 0.0175 | 255 | 49.51% | 11663.3 | 41.77% |
| Ap4(bHLH)/AML-Tfap4-ChIP-Seq(GSE45738)/Homer | NAHCAGCTGD | 1.00E-03 | -7.33E+00 | 0.0346 | 320 | 62.14% | 15349.3 | 54.97% |
| Tcf12(bHLH)/GM12878-Tcf12-ChIP-Seq(GSE32465)/Homer | VCAGCTGYTG | 1.00E-03 | -7.30E+00 | 0.0346 | 280 | 54.37% | 13165.9 | 47.15% |
| p53(p53)/mES-cMyc-ChIP-Seq(GSE11431)/Homer | ACATGCCCGGGCAT | 1.00E-03 | -7.20E+00 | 0.0346 | 13 | 2.52% | 239 | 0.86% |
| TEAD4(TEA)/Tropoblast-Tead4-ChIP-Seq(GSE37350)/Homer | CCWGGAATGY | 1.00E-02 | -6.73E+00 | 0.0394 | 272 | 52.82% | 12837.7 | 45.98% |
| Maz(Zf)/HepG2-Maz-ChIP-Seq(GSE31477)/Homer | GGGGGGGG | 1.00E-02 | -6.35E+00 | 0.0511 | 413 | 80.19% | 20823.6 | 74.57% |
| AMYB(HTH)/Testes-AMYB-ChIP-Seq(GSE44588)/Homer | TGGCAGTTGG | 1.00E-02 | -6.29E+00 | 0.0511 | 391 | 75.92% | 19552.7 | 70.02% |
| ZFX(Zf)/mES-Zfx-ChIP-Seq(GSE11431)/Homer | AGGCCTRG | 1.00E-02 | -5.95E+00 | 0.0623 | 334 | 64.85% | 16386.2 | 58.68% |
| Nkx2.5(Homeobox)/HL1-Nkx2.5.biotin-ChIP-Seq(GSE21529)/Homer | RRSCACTYAA | 1.00E-02 | -5.90E+00 | 0.0623 | 453 | 87.96% | 23296.9 | 83.43% |
| TEAD(TEA)/Fibroblast-PU.1-ChIP-Seq(Unpublished)/Homer | YCWGGAATGY | 1.00E-02 | -5.79E+00 | 0.0623 | 222 | 43.11% | 10350.6 | 37.07% |
| Egr1(Zf)/K562-Egr1-ChIP-Seq(GSE32465)/Homer | TGCGTGGGYG | 1.00E-02 | -5.72E+00 | 0.0623 | 261 | 50.68% | 12442.6 | 44.56% |
| TATA-Box(TBP)/Promoter/Homer | CCTTTTAWAGSC | 1.00E-02 | -5.29E+00 | 0.0885 | 375 | 72.82% | 18827.3 | 67.43% |
| CArG(MADS)/PUER-Srf-ChIP-Seq(Sullivan et al.)/Homer | CCATATATGGNM | 1.00E-02 | -5.26E+00 | 0.0885 | 130 | 25.24% | 5708.7 | 20.44% |
| Rbpj1(?)/Panc1-Rbpj1-ChIP-Seq(GSE47459)/Homer | HTTTCCCASG | 1.00E-02 | -5.22E+00 | 0.0885 | 389 | 75.53% | 19641.6 | 70.34% |
| Tcf4(HMG)/Hct116-Tcf4-ChIP-Seq(SRA012054)/Homer | ASATCAAAGGVA | 1.00E-02 | -5.20E+00 | 0.0885 | 170 | 33.01% | 7754.6 | 27.77% |
| Tcf3(HMG)/mES-Tcf3-ChIP-Seq(GSE11724)/Homer | ASWTCAAAGG | 1.00E-02 | -5.18E+00 | 0.0885 | 106 | 20.58% | 4527.4 | 16.21% |
| Sox10(HMG)/SciaticNerve-Sox3-ChIP-Seq(GSE35132)/Homer | CCWTTGTYYB | 1.00E-02 | -4.88E+00 | 0.1 | 388 | 75.34% | 19653.5 | 70.38% |
| NF1-halfsite(CTF)/LNCaP-NF1-ChIP-Seq(Unpublished)/Homer | YTGCCAAG | 1.00E-02 | -4.79E+00 | 0.1049 | 395 | 76.70% | 20074.3 | 71.89% |
| HOXD13(Homeobox)/Chicken-Hoxd13-ChIP-Seq(GSE38910)/Homer | NCYAATAAAA | 1.00E-02 | -4.62E+00 | 0.1188 | 303 | 58.83% | 14963 | 53.59% |
| RBPJ:Ebox(?,bHLH)/Panc1-Rbpj1-ChIP-Seq(GSE47459)/Homer | GGGRAARRGRMCAGMTG | 1.00E-01 | -4.59E+00 | 0.1188 | 126 | 24.47% | 5625.8 | 20.15% |
| AR-halfsite(NR)/LNCaP-AR-ChIP-Seq(GSE27824)/Homer | CCAGGAACAG | 1.00E-01 | -4.58E+00 | 0.1188 | 500 | 97.09% | 26487.2 | 94.86% |
| NFkB-p65-Rel(RHD)/ThioMac-LPS-Expression(GSE23622)/Homer | GGAAATTCCC | 1.00E-01 | -4.56E+00 | 0.1188 | 32 | 6.21% | 1111.8 | 3.98% |
| Meis1(Homeobox)/MastCells-Meis1-ChIP-Seq(GSE48085)/Homer | VGCTGWCAVB | 1.00E-01 | -4.29E+00 | 0.139 | 393 | 76.31% | 20067 | 71.87% |
| Stat3(Stat)/mES-Stat3-ChIP-Seq(GSE11431)/Homer | CTTCCGGGAA | 1.00E-01 | -4.28E+00 | 0.139 | 194 | 37.67% | 9194.6 | 32.93% |
| CHR(?)/Hela-CellCycle-Expression/Homer | SRGTTTCAAA | 1.00E-01 | -4.22E+00 | 0.1393 | 220 | 42.72% | 10582.8 | 37.90% |
| Sox2(HMG)/mES-Sox2-ChIP-Seq(GSE11431)/Homer | BCCATTGTTC | 1.00E-01 | -4.05E+00 | 0.1586 | 267 | 51.84% | 13140.1 | 47.06% |
| AP-1(bZIP)/ThioMac-PU.1-ChIP-Seq(GSE21512)/Homer | VTGACTCATC | 1.00E-01 | -4.02E+00 | 0.1586 | 204 | 39.61% | 9782.8 | 35.03% |
| NeuroD1(bHLH)/Islet-NeuroD1-ChIP-Seq(GSE30298)/Homer | GCCATCTGTT | 1.00E-01 | -4.00E+00 | 0.1586 | 238 | 46.21% | 11594 | 41.52% |
| MyoG(bHLH)/C2C12-MyoG-ChIP-Seq(GSE36024)/Homer | AACAGCTG | 1.00E-01 | -3.95E+00 | 0.1586 | 289 | 56.12% | 14358.1 | 51.42% |
| HNF4a(NR),DR1/HepG2-HNF4a-ChIP-Seq(GSE25021)/Homer | CARRGKBCAAAGTYCA | 1.00E-01 | -3.90E+00 | 0.1623 | 173 | 33.59% | 8183.3 | 29.31% |
| CEBP:CEBP(bZIP)/MEF-Chop-ChIP-Seq(GSE35681)/Homer | NTNATGCAAYMNNHTGMAAY | 1.00E-01 | -3.75E+00 | 0.1832 | 55 | 10.68% | 2260.1 | 8.09% |
| Atf3(bZIP)/GBM-ATF3-ChIP-Seq(GSE33912)/Homer | DATGASTCATHN | 1.00E-01 | -3.74E+00 | 0.1832 | 184 | 35.73% | 8795 | 31.50% |
| Atoh1(bHLH)/Cerebellum-Atoh1-ChIP-Seq(GSE22111)/Homer | VNRVCAGCTGGY | 1.00E-01 | -3.74E+00 | 0.1832 | 291 | 56.50% | 14522.7 | 52.01% |
| GATA:SCL(Zf,bHLH)/Ter119-SCL-ChIP-Seq(GSE18720)/Homer | CRGCTGBNGNSNNSAGATAA | 1.00E-01 | -3.58E+00 | 0.1984 | 53 | 10.29% | 2187.6 | 7.83% |
| FOXA1(Forkhead)/MCF7-FOXA1-ChIP-Seq(GSE26831)/Homer | WAAGTAAACA | 1.00E-01 | -3.52E+00 | 0.2062 | 286 | 55.53% | 14310.4 | 51.25% |
| Sox6(HMG)/Myotubes-Sox6-ChIP-Seq(GSE32627)/Homer | CCATTGTTNY | 1.00E-01 | -3.41E+00 | 0.2245 | 379 | 73.59% | 19484.1 | 69.78% |
| Olig2(bHLH)/Neuron-Olig2-ChIP-Seq(GSE30882)/Homer | RCCATMTGTT | 1.00E-01 | -3.41E+00 | 0.2245 | 403 | 78.25% | 20843.9 | 74.65% |
| RUNX1(Runt)/Jurkat-RUNX1-ChIP-Seq(GSE29180)/Homer | AAACCACARM | 1.00E-01 | -3.33E+00 | 0.2297 | 288 | 55.92% | 14470.9 | 51.82% |
| KLF5(Zf)/LoVo-KLF5-ChIP-Seq(GSE49402)/Homer | DGGGYGKGGC | 1.00E-01 | -3.31E+00 | 0.2297 | 395 | 76.70% | 20412 | 73.10% |
| FOXA1(Forkhead)/LNCAP-FOXA1-ChIP-Seq(GSE27824)/Homer | WAAGTAAACA | 1.00E-01 | -3.31E+00 | 0.2297 | 325 | 63.11% | 16505.4 | 59.11% |
| Isl1(Homeobox)/Neuron-Isl1-ChIP-Seq(GSE31456)/Homer | CTAATKGV | 1.00E-01 | -3.23E+00 | 0.2386 | 418 | 81.17% | 21744.5 | 77.87% |
| Ascl1(bHLH)/NeuralTubes-Ascl1-ChIP-Seq(GSE55840)/Homer | NNVVCAGCTGBN | 1.00E-01 | -3.22E+00 | 0.2386 | 356 | 69.13% | 18245.5 | 65.34% |
| Arnt:Ahr(bHLH)/MCF7-Arnt-ChIP-Seq(Lo et al.)/Homer | TBGCACGCAA | 1.00E-01 | -3.17E+00 | 0.24 | 199 | 38.64% | 9733.4 | 34.86% |
| Smad3(MAD)/NPC-Smad3-ChIP-Seq(GSE36673)/Homer | TWGTCTGV | 1.00E-01 | -3.16E+00 | 0.24 | 479 | 93.01% | 25340.2 | 90.75% |
| Fra1(bZIP)/BT549-Fra1-ChIP-Seq(GSE46166)/Homer | NNATGASTCATH | 1.00E-01 | -3.13E+00 | 0.2414 | 158 | 30.68% | 7588.4 | 27.18% |
| AP-2alpha(AP2)/Hela-AP2alpha-ChIP-Seq(GSE31477)/Homer | ATGCCCTGAGGC | 1.00E-01 | -3.09E+00 | 0.2453 | 253 | 49.13% | 12644.7 | 45.28% |
| NFAT(RHD)/Jurkat-NFATC1-ChIP-Seq(Jolma et al.)/Homer | ATTTTCCATT | 1.00E-01 | -3.09E+00 | 0.2453 | 265 | 51.46% | 13293.9 | 47.61% |
| GSC(Homeobox)/FrogEmbryos-GSC-ChIP-Seq(DRA000576)/Homer | RGGATTAR | 1.00E-01 | -3.08E+00 | 0.2453 | 307 | 59.61% | 15582.6 | 55.81% |
| ZNF711(Zf)/SHSY5Y-ZNF711-ChIP-Seq(GSE20673)/Homer | AGGCCTAG | 1.00E-01 | -3.04E+00 | 0.2453 | 383 | 74.37% | 19806.9 | 70.93% |
| Foxa2(Forkhead)/Liver-Foxa2-ChIP-Seq(GSE25694)/Homer | CYTGTTTACWYW | 1.00E-01 | -3.04E+00 | 0.2453 | 245 | 47.57% | 12229.2 | 43.80% |
| E2A(bHLH)/proBcell-E2A-ChIP-Seq(GSE21978)/Homer | DNRCAGCTGY | 1.00E-01 | -3.02E+00 | 0.2453 | 342 | 66.41% | 17526.2 | 62.77% |
| BORIS(Zf)/K562-CTCFL-ChIP-Seq(GSE32465)/Homer | CNNBRGCGCCCCCTGSTGGC | 1.00E-01 | -3.01E+00 | 0.2453 | 90 | 17.48% | 4115.1 | 14.74% |
| Smad2(MAD)/ES-SMAD2-ChIP-Seq(GSE29422)/Homer | CTGTCTGG | 1.00E-01 | -2.96E+00 | 0.2453 | 373 | 72.43% | 19269.9 | 69.01% |
| Cdx2(Homeobox)/mES-Cdx2-ChIP-Seq(GSE14586)/Homer | GYMATAAAAH | 1.00E-01 | -2.95E+00 | 0.2453 | 215 | 41.75% | 10647.4 | 38.13% |
| p63(p53)/Keratinocyte-p63-ChIP-Seq(GSE17611)/Homer | NNDRCATGYCYNRRCATGYH | 1.00E-01 | -2.89E+00 | 0.2522 | 115 | 22.33% | 5416.3 | 19.40% |
| BMYB(HTH)/Hela-BMYB-ChIP-Seq(GSE27030)/Homer | NHAACBGYYV | 1.00E-01 | -2.82E+00 | 0.2677 | 377 | 73.20% | 19533.8 | 69.96% |
| Egr2(Zf)/Thymocytes-Egr2-ChIP-Seq(GSE34254)/Homer | NGCGTGGGCGGR | 1.00E-01 | -2.81E+00 | 0.2677 | 84 | 16.31% | 3854 | 13.80% |
| Jun-AP1(bZIP)/K562-cJun-ChIP-Seq(GSE31477)/Homer | GATGASTCATCN | 1.00E-01 | -2.80E+00 | 0.2677 | 72 | 13.98% | 3249.1 | 11.64% |
| EBF1(EBF)/Near-E2A-ChIP-Seq(GSE21512)/Homer | GTCCCCWGGGGA | 1.00E-01 | -2.79E+00 | 0.2677 | 305 | 59.22% | 15561 | 55.73% |
| TEAD2(TEA)/Py2T-Tead2-ChIP-Seq(GSE55709)/Homer | CCWGGAATGY | 1.00E-01 | -2.76E+00 | 0.2677 | 175 | 33.98% | 8580.3 | 30.73% |
| Erra(NR)/HepG2-Erra-ChIP-Seq(GSE31477)/Homer | CAAAGGTCAG | 1.00E-01 | -2.75E+00 | 0.2677 | 427 | 82.91% | 22379.8 | 80.15% |
| PRDM9(Zf)/Testis-DMC1-ChIP-Seq(GSE35498)/Homer | ADGGYAGYAGCATCT | 1.00E-01 | -2.64E+00 | 0.2897 | 131 | 25.44% | 6308.4 | 22.59% |
| VDR(NR),DR3/GM10855-VDR+vitD-ChIP-Seq(GSE22484)/Homer | ARAGGTCANWGAGTTCANNN | 1.00E-01 | -2.64E+00 | 0.2897 | 79 | 15.34% | 3636.4 | 13.02% |
| EBF(EBF)/proBcell-EBF-ChIP-Seq(GSE21978)/Homer | DGTCCCYRGGGA | 1.00E-01 | -2.63E+00 | 0.2897 | 91 | 17.67% | 4247.4 | 15.21% |
| Nanog(Homeobox)/mES-Nanog-ChIP-Seq(GSE11724)/Homer | RGCCATTAAC | 1.00E-01 | -2.62E+00 | 0.2897 | 505 | 98.06% | 27050.5 | 96.88% |
| SPDEF(ETS)/VCaP-SPDEF-ChIP-Seq(SRA014231)/Homer | ASWTCCTGBT | 1.00E-01 | -2.53E+00 | 0.3039 | 309 | 60.00% | 15861.9 | 56.81% |
| Unknown(Homeobox)/Limb-p300-ChIP-Seq/Homer | SSCMATWAAA | 1.00E-01 | -2.52E+00 | 0.3039 | 204 | 39.61% | 10193.6 | 36.51% |
| RUNX-AML(Runt)/CD4+-PolII-ChIP-Seq(Barski et al.)/Homer | GCTGTGGTTW | 1.00E-01 | -2.44E+00 | 0.3234 | 214 | 41.55% | 10752.4 | 38.51% |
| GLI3(Zf)/Limb-GLI3-ChIP-Chip(GSE11077)/Homer | CGTGGGTGGTCC | 1.00E-01 | -2.40E+00 | 0.3322 | 51 | 9.90% | 2278 | 8.16% |
| Smad4(MAD)/ESC-SMAD4-ChIP-Seq(GSE29422)/Homer | VBSYGTCTGG | 1.00E-01 | -2.37E+00 | 0.3392 | 376 | 73.01% | 19614.2 | 70.24% |
| Bcl6(Zf)/Liver-Bcl6-ChIP-Seq(GSE31578)/Homer | NNNCTTTCCAGGAAA | 1.00E-01 | -2.33E+00 | 0.3489 | 358 | 69.51% | 18628.6 | 66.71% |
| FXR(NR),IR1/Liver-FXR-ChIP-Seq(Chong et al.)/Homer | AGGTCANTGACCTB | 1.00E+00 | -2.26E+00 | 0.3676 | 132 | 25.63% | 6468.5 | 23.17% |
| Sox4(HMG)/proB-Sox4-ChIP-Seq(GSE50066)/Homer | YCTTTGTTCC | 1.00E+00 | -2.24E+00 | 0.3684 | 253 | 49.13% | 12917.5 | 46.26% |
| Tcfcp2l1(CP2)/mES-Tcfcp2l1-ChIP-Seq(GSE11431)/Homer | NRAACCRGTTYRAACCRGYT | 1.00E+00 | -2.23E+00 | 0.3684 | 60 | 11.65% | 2761 | 9.89% |
| Esrrb(NR)/mES-Esrrb-ChIP-Seq(GSE11431)/Homer | KTGACCTTGA | 1.00E+00 | -2.22E+00 | 0.3684 | 202 | 39.22% | 10185.5 | 36.48% |
| Fosl2(bZIP)/3T3L1-Fosl2-ChIP-Seq(GSE56872)/Homer | NATGASTCABNN | 1.00E+00 | -2.20E+00 | 0.3721 | 96 | 18.64% | 4612.3 | 16.52% |
| NFkB-p65(RHD)/GM12787-p65-ChIP-Seq(GSE19485)/Homer | WGGGGATTTCCC | 1.00E+00 | -2.10E+00 | 0.4029 | 176 | 34.17% | 8842 | 31.67% |
| Foxh1(Forkhead)/hESC-FOXH1-ChIP-Seq(GSE29422)/Homer | NNTGTGGATTSS | 1.00E+00 | -2.10E+00 | 0.4029 | 189 | 36.70% | 9534.4 | 34.15% |
| GATA(Zf),IR3/iTreg-Gata3-ChIP-Seq(GSE20898)/Homer | NNNNNBAGATAWYATCTVHN | 1.00E+00 | -2.10E+00 | 0.4029 | 50 | 9.71% | 2285.8 | 8.19% |
| CEBP(bZIP)/ThioMac-CEBPb-ChIP-Seq(GSE21512)/Homer | ATTGCGCAAC | 1.00E+00 | -2.10E+00 | 0.4029 | 198 | 38.45% | 10015.1 | 35.87% |
| STAT4(Stat)/CD4-Stat4-ChIP-Seq(GSE22104)/Homer | NYTTCCWGGAAR | 1.00E+00 | -2.09E+00 | 0.4029 | 287 | 55.73% | 14818 | 53.07% |
| ERE(NR),IR3/MCF7-ERa-ChIP-Seq(Unpublished)/Homer | VAGGTCACNSTGACC | 1.00E+00 | -2.08E+00 | 0.4029 | 88 | 17.09% | 4228.5 | 15.14% |
| NFY(CCAAT)/Promoter/Homer | RGCCAATSRG | 1.00E+00 | -2.06E+00 | 0.4029 | 269 | 52.23% | 13850.9 | 49.60% |
| AP-2gamma(AP2)/MCF7-TFAP2C-ChIP-Seq(GSE21234)/Homer | SCCTSAGGSCAW | 1.00E+00 | -2.05E+00 | 0.4029 | 297 | 57.67% | 15375.4 | 55.06% |
| Mef2c(MADS)/GM12878-Mef2c-ChIP-Seq(GSE32465)/Homer | DCYAAAAATAGM | 1.00E+00 | -2.00E+00 | 0.4069 | 139 | 26.99% | 6918.5 | 24.78% |
| SCL(bHLH)/HPC7-Scl-ChIP-Seq(GSE13511)/Homer | AVCAGCTG | 1.00E+00 | -1.95E+00 | 0.4242 | 499 | 96.89% | 26766.9 | 95.86% |
| p53(p53)/Saos-p53-ChIP-Seq(GSE15780)/Homer | RRCATGYCYRGRCATGYYYN | 1.00E+00 | -1.89E+00 | 0.4405 | 34 | 6.60% | 1525.6 | 5.46% |
| p53(p53)/Saos-p53-ChIP-Seq/Homer | RRCATGYCYRGRCATGYYYN | 1.00E+00 | -1.89E+00 | 0.4405 | 34 | 6.60% | 1525.6 | 5.46% |
| RXR(NR),DR1/3T3L1-RXR-ChIP-Seq(GSE13511)/Homer | TAGGGCAAAGGTCA | 1.00E+00 | -1.84E+00 | 0.4575 | 311 | 60.39% | 16224.9 | 58.11% |
| NFkB-p50,p52(RHD)/Monocyte-p50-ChIP-Chip(Schreiber et al.)/Homer | GGGGGAATCCCC | 1.00E+00 | -1.83E+00 | 0.4575 | 47 | 9.13% | 2190.6 | 7.84% |
| Stat3+il21(Stat)/CD4-Stat3-ChIP-Seq(GSE19198)/Homer | SVYTTCCNGGAARB | 1.00E+00 | -1.81E+00 | 0.4611 | 230 | 44.66% | 11843 | 42.41% |
| Mef2a(MADS)/HL1-Mef2a.biotin-ChIP-Seq(GSE21529)/Homer | CYAAAAATAG | 1.00E+00 | -1.80E+00 | 0.4611 | 135 | 26.21% | 6773.5 | 24.26% |
| BATF(bZIP)/Th17-BATF-ChIP-Seq(GSE39756)/Homer | DATGASTCAT | 1.00E+00 | -1.77E+00 | 0.4703 | 175 | 33.98% | 8911 | 31.91% |
| HIF-1b(HLH)/T47D-HIF1b-ChIP-Seq(GSE59937)/Homer | RTACGTGC | 1.00E+00 | -1.76E+00 | 0.4703 | 278 | 53.98% | 14458.9 | 51.78% |
| PR(NR)/T47D-PR-ChIP-Seq(GSE31130)/Homer | VAGRACAKNCTGTBC | 1.00E+00 | -1.76E+00 | 0.4703 | 430 | 83.50% | 22833.7 | 81.77% |
| CRX(Homeobox)/Retina-Crx-ChIP-Seq(GSE20012)/Homer | GCTAATCC | 1.00E+00 | -1.76E+00 | 0.4703 | 444 | 86.21% | 23623.5 | 84.60% |
| PPARE(NR),DR1/3T3L1-Pparg-ChIP-Seq(GSE13511)/Homer | TGACCTTTGCCCCA | 1.00E+00 | -1.75E+00 | 0.4703 | 282 | 54.76% | 14678.9 | 52.57% |
| Nur77(NR)/K562-NR4A1-ChIP-Seq(GSE31363)/Homer | TGACCTTTNCNT | 1.00E+00 | -1.75E+00 | 0.4703 | 70 | 13.59% | 3387.5 | 12.13% |
| Nkx6.1(Homeobox)/Islet-Nkx6.1-ChIP-Seq(GSE40975)/Homer | GKTAATGR | 1.00E+00 | -1.74E+00 | 0.4703 | 440 | 85.44% | 23403.2 | 83.81% |
| Nkx3.1(Homeobox)/LNCaP-Nkx3.1-ChIP-Seq(GSE28264)/Homer | AAGCACTTAA | 1.00E+00 | -1.71E+00 | 0.4703 | 440 | 85.44% | 23413.3 | 83.85% |
| Klf4(Zf)/mES-Klf4-ChIP-Seq(GSE11431)/Homer | GCCACACCCA | 1.00E+00 | -1.69E+00 | 0.4703 | 175 | 33.98% | 8941 | 32.02% |
| Lhx2(Homeobox)/HFSC-Lhx2-ChIP-Seq(GSE48068)/Homer | TAATTAGN | 1.00E+00 | -1.68E+00 | 0.4703 | 275 | 53.40% | 14328.1 | 51.31% |
| CTCF-SatelliteElement(Zf?)/CD4+-CTCF-ChIP-Seq(Barski et al.)/Homer | TGCAGTTCCMVNWRTGGCCA | 1.00E+00 | -1.64E+00 | 0.4834 | 7 | 1.36% | 253.5 | 0.91% |
| Fox:Ebox(Forkhead,bHLH)/Panc1-Foxa2-ChIP-Seq(GSE47459)/Homer | NNNVCTGWGYAAACASN | 1.00E+00 | -1.59E+00 | 0.5011 | 278 | 53.98% | 14530.5 | 52.04% |
| PAX3:FKHR-fusion(Paired,Homeobox)/Rh4-PAX3:FKHR-ChIP-Seq(GSE19063)/Homer | ACCRTGACTAATTNN | 1.00E+00 | -1.58E+00 | 0.5013 | 66 | 12.82% | 3226.8 | 11.56% |
| Sp1(Zf)/Promoter/Homer | GGCCCCGCCCCC | 1.00E+00 | -1.57E+00 | 0.5062 | 185 | 35.92% | 9528 | 34.12% |
| Znf263(Zf)/K562-Znf263-ChIP-Seq(GSE31477)/Homer | CVGTSCTCCC | 1.00E+00 | -1.55E+00 | 0.5091 | 394 | 76.50% | 20904 | 74.86% |
| CTCF(Zf)/CD4+-CTCF-ChIP-Seq(Barski et al.)/Homer | AYAGTGCCMYCTRGTGGCCA | 1.00E+00 | -1.54E+00 | 0.5091 | 55 | 10.68% | 2667.4 | 9.55% |
| E2F6(E2F)/Hela-E2F6-ChIP-Seq(GSE31477)/Homer | GGCGGGAARN | 1.00E+00 | -1.53E+00 | 0.5124 | 178 | 34.56% | 9169.7 | 32.84% |
| Hoxc9(Homeobox)/Ainv15-Hoxc9-ChIP-Seq(GSE21812)/Homer | GGCCATAAATCA | 1.00E+00 | -1.51E+00 | 0.5142 | 154 | 29.90% | 7892.1 | 28.26% |
| MYB(HTH)/ERMYB-Myb-ChIPSeq(GSE22095)/Homer | GGCVGTTR | 1.00E+00 | -1.51E+00 | 0.5142 | 397 | 77.09% | 21087.6 | 75.52% |
| EKLF(Zf)/Erythrocyte-Klf1-ChIP-Seq(GSE20478)/Homer | NWGGGTGTGGCY | 1.00E+00 | -1.47E+00 | 0.5291 | 89 | 17.28% | 4465.1 | 15.99% |
| Hoxb4(Homeobox)/ES-Hoxb4-ChIP-Seq(GSE34014)/Homer | TGATTRATGGCY | 1.00E+00 | -1.39E+00 | 0.5674 | 59 | 11.46% | 2918 | 10.45% |
| Hnf1(Homeobox)/Liver-Foxa2-Chip-Seq(GSE25694)/Homer | GGTTAAWCATTAA | 1.00E+00 | -1.36E+00 | 0.58 | 52 | 10.10% | 2562.6 | 9.18% |
| PRDM14(Zf)/H1-PRDM14-ChIP-Seq(GSE22767)/Homer | RGGTCTCTAACY | 1.00E+00 | -1.35E+00 | 0.58 | 121 | 23.50% | 6202.8 | 22.21% |
| Ptf1a(bHLH)/Panc1-Ptf1a-ChIP-Seq(GSE47459)/Homer | ACAGCTGTTN | 1.00E+00 | -1.34E+00 | 0.5839 | 441 | 85.63% | 23596 | 84.50% |
| RUNX(Runt)/HPC7-Runx1-ChIP-Seq(GSE22178)/Homer | SAAACCACAG | 1.00E+00 | -1.32E+00 | 0.5877 | 207 | 40.19% | 10821.9 | 38.76% |
| FOXA1:AR(Forkhead,NR)/LNCAP-AR-ChIP-Seq(GSE27824)/Homer | AGTAAACAAAAAAGAACAND | 1.00E+00 | -1.31E+00 | 0.5894 | 24 | 4.66% | 1129.6 | 4.05% |
| NFAT:AP1(RHD,bZIP)/Jurkat-NFATC1-ChIP-Seq(Jolma et al.)/Homer | SARTGGAAAAWRTGAGTCAB | 1.00E+00 | -1.30E+00 | 0.5911 | 60 | 11.65% | 2998.2 | 10.74% |
| Phox2a(Homeobox)/Neuron-Phox2a-ChIP-Seq(GSE31456)/Homer | YTAATYNRATTA | 1.00E+00 | -1.26E+00 | 0.6101 | 117 | 22.72% | 6029 | 21.59% |
| Gata4(Zf)/Heart-Gata4-ChIP-Seq(GSE35151)/Homer | NBWGATAAGR | 1.00E+00 | -1.25E+00 | 0.6101 | 275 | 53.40% | 14532.1 | 52.04% |
| HIF-1a(bHLH)/MCF7-HIF1a-ChIP-Seq(GSE28352)/Homer | TACGTGCV | 1.00E+00 | -1.23E+00 | 0.6159 | 78 | 15.15% | 3968.7 | 14.21% |
| HNF6(Homeobox)/Liver-Hnf6-ChIP-Seq(ERP000394)/Homer | NTATYGATCH | 1.00E+00 | -1.21E+00 | 0.6278 | 158 | 30.68% | 8244.8 | 29.53% |
| Tbx5(T-box)/HL1-Tbx5.biotin-ChIP-Seq(GSE21529)/Homer | AGGTGTCA | 1.00E+00 | -1.20E+00 | 0.6278 | 478 | 92.82% | 25710.1 | 92.07% |
| RUNX2(Runt)/PCa-RUNX2-ChIP-Seq(GSE33889)/Homer | NWAACCACADNN | 1.00E+00 | -1.19E+00 | 0.6278 | 230 | 44.66% | 12128.4 | 43.43% |
| Tbx20(T-box)/Heart-Tbx20-ChIP-Seq(GSE29636)/Homer | GGTGYTGACAGS | 1.00E+00 | -1.16E+00 | 0.6416 | 74 | 14.37% | 3783.9 | 13.55% |
| Oct4(POU,Homeobox)/mES-Oct4-ChIP-Seq(GSE11431)/Homer | ATTTGCATAW | 1.00E+00 | -1.14E+00 | 0.6523 | 135 | 26.21% | 7045.8 | 25.23% |
| YY1(Zf)/Promoter/Homer | CAAGATGGCGGC | 1.00E+00 | -1.13E+00 | 0.6523 | 24 | 4.66% | 1166.9 | 4.18% |
| Oct4:Sox17(POU,Homeobox,HMG)/F9-Sox17-ChIP-Seq(GSE44553)/Homer | CCATTGTATGCAAAT | 1.00E+00 | -1.13E+00 | 0.6523 | 46 | 8.93% | 2316.7 | 8.30% |
| Reverb(NR),DR2/RAW-Reverba.biotin-ChIP-Seq(GSE45914)/Homer | GTRGGTCASTGGGTCA | 1.00E+00 | -1.10E+00 | 0.6615 | 45 | 8.74% | 2272.8 | 8.14% |
| T1ISRE(IRF)/ThioMac-Ifnb-Expression/Homer | ACTTTCGTTTCT | 1.00E+00 | -1.09E+00 | 0.6616 | 5 | 0.97% | 206.5 | 0.74% |
| Pdx1(Homeobox)/Islet-Pdx1-ChIP-Seq(SRA008281)/Homer | YCATYAATCA | 1.00E+00 | -1.08E+00 | 0.6616 | 245 | 47.57% | 12998.7 | 46.55% |
| Tlx?(NR)/NPC-H3K4me1-ChIP-Seq(GSE16256)/Homer | CTGGCAGSCTGCCA | 1.00E+00 | -1.06E+00 | 0.6725 | 133 | 25.83% | 6975.1 | 24.98% |
| Tbet(T-box)/CD8-Tbet-ChIP-Seq(GSE33802)/Homer | AGGTGTGAAM | 1.00E+00 | -1.06E+00 | 0.6725 | 284 | 55.15% | 15127.2 | 54.17% |
| NF1(CTF)/LNCAP-NF1-ChIP-Seq(Unpublished)/Homer | CYTGGCABNSTGCCAR | 1.00E+00 | -1.06E+00 | 0.6725 | 124 | 24.08% | 6495.6 | 23.26% |
| Pitx1(Homeobox)/Chicken-Pitx1-ChIP-Seq(GSE38910)/Homer | TAATCCCN | 1.00E+00 | -1.04E+00 | 0.6725 | 502 | 97.48% | 27105.2 | 97.07% |
| Ets1-distal(ETS)/CD4+-PolII-ChIP-Seq(Barski et al.)/Homer | MACAGGAAGT | 1.00E+00 | -1.03E+00 | 0.6748 | 110 | 21.36% | 5758.3 | 20.62% |
| Unknown-ESC-element(?)/mES-Nanog-ChIP-Seq(GSE11724)/Homer | CACAGCAGGGGG | 1.00E+00 | -1.03E+00 | 0.6748 | 171 | 33.20% | 9035.3 | 32.36% |
| Srebp2(bHLH)/HepG2-Srebp2-ChIP-Seq(GSE31477)/Homer | CGGTCACSCCAC | 1.00E+00 | -9.96E-01 | 0.687 | 46 | 8.93% | 2358.8 | 8.45% |
| HRE(HSF)/Striatum-HSF1-ChIP-Seq(GSE38000)/Homer | TTCTAGAABNTTCTA | 1.00E+00 | -9.90E-01 | 0.687 | 84 | 16.31% | 4384.2 | 15.70% |
| Rfx5(HTH)/GM12878-Rfx5-ChIP-Seq(GSE31477)/Homer | SCCTAGCAACAG | 1.00E+00 | -9.88E-01 | 0.687 | 108 | 20.97% | 5670.9 | 20.31% |
| RARg(NR)/ES-RARg-ChIP-Seq(GSE30538)/Homer | AGGTCAAGGTCA | 1.00E+00 | -9.61E-01 | 0.6962 | 12 | 2.33% | 578.9 | 2.07% |
| GATA3(Zf),DR4/iTreg-Gata3-ChIP-Seq(GSE20898)/Homer | AGATGKDGAGATAAG | 1.00E+00 | -9.59E-01 | 0.6962 | 28 | 5.44% | 1417.7 | 5.08% |
| E2F(E2F)/Hela-CellCycle-Expression/Homer | TTSGCGCGAAAA | 1.00E+00 | -9.41E-01 | 0.7008 | 23 | 4.47% | 1158.2 | 4.15% |
| STAT1(Stat)/HelaS3-STAT1-ChIP-Seq(GSE12782)/Homer | NATTTCCNGGAAAT | 1.00E+00 | -9.39E-01 | 0.7008 | 109 | 21.17% | 5748.8 | 20.59% |
| Rfx1(HTH)/NPC-H3K4me1-ChIP-Seq(GSE16256)/Homer | KGTTGCCATGGCAA | 1.00E+00 | -9.27E-01 | 0.7012 | 72 | 13.98% | 3770.5 | 13.50% |
| MafF(bZIP)/HepG2-MafF-ChIP-Seq(GSE31477)/Homer | HWWGTCAGCAWWTTT | 1.00E+00 | -9.26E-01 | 0.7012 | 75 | 14.56% | 3931.8 | 14.08% |
| NF1:FOXA1(CTF,Forkhead)/LNCAP-FOXA1-ChIP-Seq(GSE27824)/Homer | WNTGTTTRYTTTGGCA | 1.00E+00 | -9.19E-01 | 0.7012 | 18 | 3.50% | 901 | 3.23% |
| Bach2(bZIP)/OCILy7-Bach2-ChIP-Seq(GSE44420)/Homer | TGCTGAGTCA | 1.00E+00 | -8.89E-01 | 0.7143 | 54 | 10.49% | 2824 | 10.11% |
| GATA3(Zf)/iTreg-Gata3-ChIP-Seq(GSE20898)/Homer | AGATAASR | 1.00E+00 | -8.53E-01 | 0.7351 | 351 | 68.16% | 18892.1 | 67.66% |
| Lhx3(Homeobox)/Neuron-Lhx3-ChIP-Seq(GSE31456)/Homer | ADBTAATTAR | 1.00E+00 | -8.45E-01 | 0.7362 | 359 | 69.71% | 19332.5 | 69.23% |
| IRF1(IRF)/PBMC-IRF1-ChIP-Seq(GSE43036)/Homer | GAAAGTGAAAGT | 1.00E+00 | -8.45E-01 | 0.7362 | 48 | 9.32% | 2519.8 | 9.02% |
| Six1(Homeobox)/Myoblast-Six1-ChIP-Chip(GSE20150)/Homer | GKVTCADRTTWC | 1.00E+00 | -8.39E-01 | 0.7362 | 80 | 15.53% | 4240.6 | 15.19% |
| GRE(NR),IR3/A549-GR-ChIP-Seq(GSE32465)/Homer | NRGVACABNVTGTYCY | 1.00E+00 | -8.38E-01 | 0.7362 | 42 | 8.16% | 2200.6 | 7.88% |
| HIF2a(bHLH)/785_O-HIF2a-ChIP-Seq(GSE34871)/Homer | GCACGTACCC | 1.00E+00 | -8.26E-01 | 0.7362 | 105 | 20.39% | 5593.1 | 20.03% |
| Nr5a2(NR)/Pancreas-LRH1-ChIP-Seq(GSE34295)/Homer | BTCAAGGTCA | 1.00E+00 | -8.12E-01 | 0.7372 | 207 | 40.19% | 11112.8 | 39.80% |
| Nrf2(bZIP)/Lymphoblast-Nrf2-ChIP-Seq(GSE37589)/Homer | HTGCTGAGTCAT | 1.00E+00 | -8.12E-01 | 0.7372 | 12 | 2.33% | 607 | 2.17% |
| EWS:FLI1-fusion(ETS)/SK_N_MC-EWS:FLI1-ChIP-Seq(SRA014231)/Homer | VACAGGAAAT | 1.00E+00 | -7.97E-01 | 0.7392 | 195 | 37.86% | 10473.4 | 37.51% |
| ZNF143\|STAF(Zf)/CUTLL-ZNF143-ChIP-Seq(GSE29600)/Homer | ATTTCCCAGVAKSCY | 1.00E+00 | -7.90E-01 | 0.7397 | 126 | 24.47% | 6747.2 | 24.16% |
| Brachyury(T-box)/Mesoendoderm-Brachyury-ChIP-exo(GSE54963)/Homer | ANTTMRCASBNNNGTGYKAAN | 1.00E+00 | -7.69E-01 | 0.7507 | 83 | 16.12% | 4437.6 | 15.89% |
| EWS:ERG-fusion(ETS)/CADO_ES1-EWS:ERG-ChIP-Seq(SRA014231)/Homer | ATTTCCTGTN | 1.00E+00 | -7.61E-01 | 0.7523 | 222 | 43.11% | 11960.6 | 42.83% |
| LXRE(NR),DR4/RAW-LXRb.biotin-ChIP-Seq(GSE21512)/Homer | RGGTTACTANAGGTCA | 1.00E+00 | -7.57E-01 | 0.7523 | 16 | 3.11% | 832.1 | 2.98% |
| ERG(ETS)/VCaP-ERG-ChIP-Seq(GSE14097)/Homer | ACAGGAAGTG | 1.00E+00 | -7.51E-01 | 0.7523 | 387 | 75.15% | 20913.6 | 74.90% |
| TCFL2(HMG)/K562-TCF7L2-ChIP-Seq(GSE29196)/Homer | ACWTCAAAGG | 1.00E+00 | -7.41E-01 | 0.7533 | 29 | 5.63% | 1536.7 | 5.50% |
| Pax7(Paired,Homeobox)/Myoblast-Pax7-ChIP-Seq(GSE25064)/Homer | TAATCAATTA | 1.00E+00 | -7.36E-01 | 0.7533 | 28 | 5.44% | 1484.6 | 5.32% |
| RORgt(NR)/EL4-RORgt.Flag-ChIP-Seq(GSE56019)/Homer | AAYTAGGTCA | 1.00E+00 | -7.27E-01 | 0.7552 | 41 | 7.96% | 2189.5 | 7.84% |
| Sox3(HMG)/NPC-Sox3-ChIP-Seq(GSE33059)/Homer | CCWTTGTY | 1.00E+00 | -7.25E-01 | 0.7552 | 383 | 74.37% | 20713.4 | 74.18% |
| STAT6(Stat)/Macrophage-Stat6-ChIP-Seq(GSE38377)/Homer | TTCCKNAGAA | 1.00E+00 | -7.16E-01 | 0.7552 | 175 | 33.98% | 9448 | 33.84% |
| X-box(HTH)/NPC-H3K4me1-ChIP-Seq(GSE16256)/Homer | GGTTGCCATGGCAA | 1.00E+00 | -7.12E-01 | 0.7552 | 41 | 7.96% | 2195.4 | 7.86% |
| Pax7(Paired,Homeobox),long/Myoblast-Pax7-ChIP-Seq(GSE25064)/Homer | TAATCHGATTAC | 1.00E+00 | -7.09E-01 | 0.7552 | 7 | 1.36% | 358.8 | 1.28% |
| Pax8(Paired,Homeobox)/Thyroid-Pax8-ChIP-Seq(GSE26938)/Homer | GTCATGCHTGRCTGS | 1.00E+00 | -7.05E-01 | 0.7552 | 96 | 18.64% | 5176.7 | 18.54% |
| THRa(NR)/C17.2-THRa-ChIP-Seq(GSE38347)/Homer | GGTCANYTGAGGWCA | 1.00E+00 | -6.99E-01 | 0.7552 | 129 | 25.05% | 6967.6 | 24.95% |
| OCT4-SOX2-TCF-NANOG(POU,Homeobox,HMG)/mES-Oct4-ChIP-Seq(GSE11431)/Homer | ATTTGCATAACAATG | 1.00E+00 | -6.94E-01 | 0.7552 | 55 | 10.68% | 2961.3 | 10.61% |
| Usf2(bHLH)/C2C12-Usf2-ChIP-Seq(GSE36030)/Homer | GTCACGTGGT | 1.00E+00 | -6.87E-01 | 0.7552 | 93 | 18.06% | 5024.7 | 17.99% |
| STAT6(Stat)/CD4-Stat6-ChIP-Seq(GSE22104)/Homer | ABTTCYYRRGAA | 1.00E+00 | -6.78E-01 | 0.7552 | 171 | 33.20% | 9258.1 | 33.16% |
| MafK(bZIP)/C2C12-MafK-ChIP-Seq(GSE36030)/Homer | GCTGASTCAGCA | 1.00E+00 | -6.65E-01 | 0.7582 | 62 | 12.04% | 3355.7 | 12.02% |
| MafA(bZIP)/Islet-MafA-ChIP-Seq(GSE30298)/Homer | TGCTGACTCA | 1.00E+00 | -6.65E-01 | 0.7582 | 210 | 40.78% | 11382.1 | 40.76% |
| IRF2(IRF)/Erythroblas-IRF2-ChIP-Seq(GSE36985)/Homer | GAAASYGAAASY | 1.00E+00 | -6.49E-01 | 0.7625 | 36 | 6.99% | 1950.5 | 6.99% |
| Gata1(Zf)/K562-GATA1-ChIP-Seq(GSE18829)/Homer | SAGATAAGRV | 1.00E+00 | -6.28E-01 | 0.7738 | 175 | 33.98% | 9513.2 | 34.07% |
| NPAS2(bHLH)/Liver-NPAS2-ChIP-Seq(GSE39860)/Homer | KCCACGTGAC | 1.00E+00 | -6.27E-01 | 0.7738 | 258 | 50.10% | 14014.9 | 50.19% |
| CLOCK(bHLH)/Liver-Clock-ChIP-Seq(GSE39860)/Homer | GHCACGTG | 1.00E+00 | -6.24E-01 | 0.7738 | 154 | 29.90% | 8377.1 | 30.00% |
| Nr5a2(NR)/mES-Nr5a2-ChIP-Seq(GSE19019)/Homer | BTCAAGGTCA | 1.00E+00 | -6.01E-01 | 0.7825 | 161 | 31.26% | 8775.4 | 31.43% |
| CEBP:AP1(bZIP)/ThioMac-CEBPb-ChIP-Seq(GSE21512)/Homer | DRTGTTGCAA | 1.00E+00 | -5.98E-01 | 0.7825 | 198 | 38.45% | 10786.5 | 38.63% |
| bHLHE40(bHLH)/HepG2-BHLHE40-ChIP-Seq(GSE31477)/Homer | KCACGTGMCN | 1.00E+00 | -5.71E-01 | 0.798 | 89 | 17.28% | 4881.3 | 17.48% |
| HOXA9(Homeobox)/HSC-Hoxa9-ChIP-Seq(GSE33509)/Homer | GGCCATAAATCA | 1.00E+00 | -5.64E-01 | 0.7992 | 184 | 35.73% | 10055.8 | 36.01% |
| Foxo1(Forkhead)/RAW-Foxo1-ChIP-Seq(Fan et al.)/Homer | CTGTTTAC | 1.00E+00 | -5.27E-01 | 0.8244 | 393 | 76.31% | 21396.2 | 76.63% |
| ETS:E-box(ETS,bHLH)/HPC7-Scl-ChIP-Seq(GSE22178)/Homer | AGGAARCAGCTG | 1.00E+00 | -5.24E-01 | 0.8244 | 30 | 5.83% | 1675.8 | 6.00% |
| TR4(NR),DR1/Hela-TR4-ChIP-Seq(GSE24685)/Homer | GAGGTCAAAGGTCA | 1.00E+00 | -5.21E-01 | 0.8244 | 40 | 7.77% | 2228.3 | 7.98% |
| GRE(NR),IR3/RAW264.7-GRE-ChIP-Seq(Unpublished)/Homer | VAGRACAKWCTGTYC | 1.00E+00 | -5.17E-01 | 0.8244 | 71 | 13.79% | 3933.1 | 14.09% |
| Gata2(Zf)/K562-GATA2-ChIP-Seq(GSE18829)/Homer | BBCTTATCTS | 1.00E+00 | -5.07E-01 | 0.8244 | 190 | 36.89% | 10432.6 | 37.36% |
| PRDM1(Zf)/Hela-PRDM1-ChIP-Seq(GSE31477)/Homer | ACTTTCACTTTC | 1.00E+00 | -4.92E-01 | 0.8323 | 171 | 33.20% | 9413.9 | 33.71% |
| n-Myc(bHLH)/mES-nMyc-ChIP-Seq(GSE11431)/Homer | VRCCACGTGG | 1.00E+00 | -4.81E-01 | 0.8367 | 176 | 34.17% | 9695.7 | 34.72% |
| c-Myc(bHLH)/LNCAP-cMyc-ChIP-Seq(Unpublished)/Homer | VCCACGTG | 1.00E+00 | -4.73E-01 | 0.8395 | 131 | 25.44% | 7250.4 | 25.97% |
| Tbox:Smad(T-box,MAD)/ESCd5-Smad2_3-ChIP-Seq(GSE29422)/Homer | AGGTGHCAGACA | 1.00E+00 | -4.63E-01 | 0.8436 | 59 | 11.46% | 3310.9 | 11.86% |
| Max(bHLH)/K562-Max-ChIP-Seq(GSE31477)/Homer | RCCACGTGGYYN | 1.00E+00 | -4.56E-01 | 0.8451 | 168 | 32.62% | 9284.3 | 33.25% |
| Rfx2(HTH)/LoVo-RFX2-ChIP-Seq(GSE49402)/Homer | GTTGCCATGGCAACM | 1.00E+00 | -4.45E-01 | 0.8503 | 35 | 6.80% | 1992.3 | 7.14% |
| EHF(ETS)/LoVo-EHF-ChIP-Seq(GSE49402)/Homer | AVCAGGAAGT | 1.00E+00 | -4.42E-01 | 0.8503 | 337 | 65.44% | 18457.3 | 66.10% |
| E-box(bHLH)/Promoter/Homer | SSGGTCACGTGA | 1.00E+00 | -4.36E-01 | 0.8503 | 28 | 5.44% | 1606.6 | 5.75% |
| Pit1+1bp(Homeobox)/GCrat-Pit1-ChIP-Seq(GSE58009)/Homer | ATGCATAATTCA | 1.00E+00 | -4.35E-01 | 0.8503 | 96 | 18.64% | 5367.5 | 19.22% |
| PU.1-IRF(ETS:IRF)/Bcell-PU.1-ChIP-Seq(GSE21512)/Homer | MGGAAGTGAAAC | 1.00E+00 | -4.03E-01 | 0.8693 | 336 | 65.24% | 18444.4 | 66.05% |
| Mouse_Recombination_Hotspot(Zf)/Testis-DMC1-ChIP-Seq(GSE24438)/Homer | ACTYKNATTCGTGNTACTTC | 1.00E+00 | -3.96E-01 | 0.8712 | 20 | 3.88% | 1175.4 | 4.21% |
| FOXP1(Forkhead)/H9-FOXP1-ChIP-Seq(GSE31006)/Homer | NYYTGTTTACHN | 1.00E+00 | -3.84E-01 | 0.8776 | 132 | 25.63% | 7391.4 | 26.47% |
| Atf1(bZIP)/K562-ATF1-ChIP-Seq(GSE31477)/Homer | GATGACGTCA | 1.00E+00 | -3.77E-01 | 0.8792 | 181 | 35.15% | 10077.8 | 36.09% |
| GFY(?)/Promoter/Homer | ACTACAATTCCC | 1.00E+00 | -3.77E-01 | 0.8792 | 34 | 6.60% | 1976.1 | 7.08% |
| USF1(bHLH)/GM12878-Usf1-ChIP-Seq(GSE32465)/Homer | SGTCACGTGR | 1.00E+00 | -3.40E-01 | 0.9033 | 127 | 24.66% | 7164.9 | 25.66% |
| PAX5(Paired,Homeobox)/GM12878-PAX5-ChIP-Seq(GSE32465)/Homer | GCAGCCAAGCRTGACH | 1.00E+00 | -3.40E-01 | 0.9033 | 105 | 20.39% | 5953.5 | 21.32% |
| bZIP:IRF(bZIP,IRF)/Th17-BatF-ChIP-Seq(GSE39756)/Homer | NAGTTTCABTHTGACTNW | 1.00E+00 | -3.38E-01 | 0.9033 | 126 | 24.47% | 7111.6 | 25.47% |
| E2A(bHLH),near_PU.1/Bcell-PU.1-ChIP-Seq(GSE21512)/Homer | NVCACCTGBN | 1.00E+00 | -3.30E-01 | 0.9033 | 323 | 62.72% | 17830 | 63.85% |
| E2F1(E2F)/Hela-E2F1-ChIP-Seq(GSE22478)/Homer | CWGGCGGGAA | 1.00E+00 | -3.28E-01 | 0.9033 | 77 | 14.95% | 4416 | 15.82% |
| c-Jun-CRE(bZIP)/K562-cJun-ChIP-Seq(GSE31477)/Homer | ATGACGTCATCY | 1.00E+00 | -2.96E-01 | 0.9218 | 88 | 17.09% | 5060.1 | 18.12% |
| EBNA1(EBV virus)/Raji-EBNA1-ChIP-Seq(GSE30709)/Homer | GGYAGCAYDTGCTDCCCNNN | 1.00E+00 | -2.83E-01 | 0.9297 | 4 | 0.78% | 278.1 | 1.00% |
| BMAL1(bHLH)/Liver-Bmal1-ChIP-Seq(GSE39860)/Homer | GNCACGTG | 1.00E+00 | -2.70E-01 | 0.9375 | 351 | 68.16% | 19412.8 | 69.52% |
| Chop(bZIP)/MEF-Chop-ChIP-Seq(GSE35681)/Homer | ATTGCATCAT | 1.00E+00 | -2.67E-01 | 0.9375 | 53 | 10.29% | 3133.9 | 11.22% |
| c-Myc(bHLH)/mES-cMyc-ChIP-Seq(GSE11431)/Homer | VVCCACGTGG | 1.00E+00 | -2.65E-01 | 0.9375 | 124 | 24.08% | 7092.1 | 25.40% |
| GFY-Staf(?,Zf)/Promoter/Homer | RACTACAATTCCCAGAAKGC | 1.00E+00 | -2.59E-01 | 0.9375 | 26 | 5.05% | 1600.6 | 5.73% |
| MITF(bHLH)/MastCells-MITF-ChIP-Seq(GSE48085)/Homer | RTCATGTGAC | 1.00E+00 | -2.53E-01 | 0.9375 | 232 | 45.05% | 13023 | 46.64% |
| CRE(bZIP)/Promoter/Homer | CSGTGACGTCAC | 1.00E+00 | -2.35E-01 | 0.949 | 74 | 14.37% | 4351.8 | 15.59% |
| Eomes(T-box)/H9-Eomes-ChIP-Seq(GSE26097)/Homer | ATTAACACCT | 1.00E+00 | -2.17E-01 | 0.9611 | 410 | 79.61% | 22619 | 81.00% |
| Pbx3(Homeobox)/GM12878-PBX3-ChIP-Seq(GSE32465)/Homer | SCTGTCAMTCAN | 1.00E+00 | -2.17E-01 | 0.9611 | 76 | 14.76% | 4486.1 | 16.07% |
| RFX(HTH)/K562-RFX3-ChIP-Seq(SRA012198)/Homer | CGGTTGCCATGGCAAC | 1.00E+00 | -2.16E-01 | 0.9611 | 29 | 5.63% | 1810.4 | 6.48% |
| E2F4(E2F)/K562-E2F4-ChIP-Seq(GSE31477)/Homer | GGCGGGAAAH | 1.00E+00 | -2.15E-01 | 0.9611 | 139 | 26.99% | 7995.8 | 28.64% |
| ARE(NR)/LNCAP-AR-ChIP-Seq(GSE27824)/Homer | RGRACASNSTGTYCYB | 1.00E+00 | -2.14E-01 | 0.9611 | 69 | 13.40% | 4096.3 | 14.67% |
| ELF5(ETS)/T47D-ELF5-ChIP-Seq(GSE30407)/Homer | ACVAGGAAGT | 1.00E+00 | -2.12E-01 | 0.9611 | 224 | 43.50% | 12658.4 | 45.33% |
| PU.1(ETS)/ThioMac-PU.1-ChIP-Seq(GSE21512)/Homer | AGAGGAAGTG | 1.00E+00 | -1.95E-01 | 0.9611 | 158 | 30.68% | 9079.4 | 32.52% |
| Pit1(Homeobox)/GCrat-Pit1-ChIP-Seq(GSE58009)/Homer | ATGMATATDC | 1.00E+00 | -1.88E-01 | 0.9611 | 240 | 46.60% | 13573.7 | 48.61% |
| GRHL2(CP2)/HBE-GRHL2-ChIP-Seq(GSE46194)/Homer | AAACYKGTTWDACMRGTTTB | 1.00E+00 | -1.88E-01 | 0.9611 | 107 | 20.78% | 6267 | 22.44% |
| GATA3(Zf),DR8/iTreg-Gata3-ChIP-Seq(GSE20898)/Homer | AGATSTNDNNDSAGATAASN | 1.00E+00 | -1.86E-01 | 0.9611 | 18 | 3.50% | 1188.4 | 4.26% |
| Srebp1a(bHLH)/HepG2-Srebp1a-ChIP-Seq(GSE31477)/Homer | RTCACSCCAY | 1.00E+00 | -1.83E-01 | 0.9611 | 60 | 11.65% | 3629.3 | 13.00% |
| Atf2(bZIP)/3T3L1-Atf2-ChIP-Seq(GSE56872)/Homer | NRRTGACGTCAT | 1.00E+00 | -1.76E-01 | 0.9611 | 95 | 18.45% | 5615.7 | 20.11% |
| STAT5(Stat)/mCD4+-Stat5-ChIP-Seq(GSE12346)/Homer | RTTTCTNAGAAA | 1.00E+00 | -1.48E-01 | 0.9776 | 112 | 21.75% | 6621.4 | 23.71% |
| E2F7(E2F)/Hela-E2F7-ChIP-Seq(GSE32673)/Homer | VDTTTCCCGCCA | 1.00E+00 | -1.47E-01 | 0.9776 | 40 | 7.77% | 2529.8 | 9.06% |
| IRF4(IRF)/GM12878-IRF4-ChIP-Seq(GSE32465)/Homer | ACTGAAACCA | 1.00E+00 | -1.37E-01 | 0.98 | 120 | 23.30% | 7092.5 | 25.40% |
| NF-E2(bZIP)/K562-NFE2-ChIP-Seq(GSE31477)/Homer | GATGACTCAGCA | 1.00E+00 | -1.30E-01 | 0.982 | 10 | 1.94% | 743.6 | 2.66% |
| ETS:RUNX(ETS,Runt)/Jurkat-RUNX1-ChIP-Seq(GSE17954)/Homer | RCAGGATGTGGT | 1.00E+00 | -1.25E-01 | 0.9834 | 28 | 5.44% | 1854.3 | 6.64% |
| GATA(Zf),IR4/iTreg-Gata3-ChIP-Seq(GSE20898)/Homer | NAGATWNBNATCTNN | 1.00E+00 | -1.19E-01 | 0.985 | 21 | 4.08% | 1440.2 | 5.16% |
| Atf7(bZIP)/3T3L1-Atf7-ChIP-Seq(GSE56872)/Homer | NGRTGACGTCAY | 1.00E+00 | -1.16E-01 | 0.985 | 126 | 24.47% | 7477.8 | 26.78% |
| JunD(bZIP)/K562-JunD-ChIP-Seq/Homer | ATGACGTCATCN | 1.00E+00 | -9.52E-02 | 1 | 28 | 5.44% | 1901.2 | 6.81% |
| HRE(HSF)/HepG2-HSF1-ChIP-Seq(GSE31477)/Homer | BSTTCTRGAABVTTCYAGAA | 1.00E+00 | -8.20E-02 | 1 | 47 | 9.13% | 3061.3 | 10.96% |
| HOXA2(Homeobox)/mES-Hoxa2-ChIP-Seq(Donaldson et al.)/Homer | GYCATCMATCAT | 1.00E+00 | -4.85E-02 | 1 | 21 | 4.08% | 1573 | 5.63% |
| ZBTB33(Zf)/GM12878-ZBTB33-ChIP-Seq(GSE32465)/Homer | GGVTCTCGCGAGAAC | 1.00E+00 | -4.36E-02 | 1 | 11 | 2.14% | 934 | 3.34% |
| Atf4(bZIP)/MEF-Atf4-ChIP-Seq(GSE35681)/Homer | MTGATGCAAT | 1.00E+00 | -4.08E-02 | 1 | 62 | 12.04% | 4088.3 | 14.64% |
| ISRE(IRF)/ThioMac-LPS-Expression(GSE23622)/Homer | AGTTTCASTTTC | 1.00E+00 | -3.03E-02 | 1 | 13 | 2.52% | 1110.4 | 3.98% |
| PBX1(Homeobox)/MCF7-PBX1-ChIP-Seq(GSE28007)/Homer | GSCTGTCACTCA | 1.00E+00 | -2.60E-02 | 1 | 21 | 4.08% | 1658.6 | 5.94% |
| Gfi1b(Zf)/HPC7-Gfi1b-ChIP-Seq(GSE22178)/Homer | MAATCACTGC | 1.00E+00 | -2.36E-02 | 1 | 160 | 31.07% | 9818.6 | 35.16% |
| Fli1(ETS)/CD8-FLI-ChIP-Seq(GSE20898)/Homer | NRYTTCCGGH | 1.00E+00 | -2.11E-02 | 1 | 304 | 59.03% | 17681.8 | 63.32% |
| Bach1(bZIP)/K562-Bach1-ChIP-Seq(GSE31477)/Homer | AWWNTGCTGAGTCAT | 1.00E+00 | -1.87E-02 | 1 | 7 | 1.36% | 733.8 | 2.63% |
| PAX5(Paired,Homeobox),condensed/GM12878-PAX5-ChIP-Seq(GSE32465)/Homer | GTCACGCTCSCTGM | 1.00E+00 | -1.85E-02 | 1 | 20 | 3.88% | 1636.4 | 5.86% |
| ETV1(ETS)/GIST48-ETV1-ChIP-Seq(GSE22441)/Homer | AACCGGAAGT | 1.00E+00 | -1.59E-02 | 1 | 338 | 65.63% | 19536 | 69.96% |
| Oct2(POU,Homeobox)/Bcell-Oct2-ChIP-Seq(GSE21512)/Homer | ATATGCAAAT | 1.00E+00 | -1.53E-02 | 1 | 70 | 13.59% | 4754.1 | 17.03% |
| Pax7(Paired,Homeobox),longest/Myoblast-Pax7-ChIP-Seq(GSE25064)/Homer | NTAATTDGCYAATTANNWWD | 1.00E+00 | -1.52E-02 | 1 | 3 | 0.58% | 427.4 | 1.53% |
| NRF1(NRF)/MCF7-NRF1-ChIP-Seq(Unpublished)/Homer | CTGCGCATGCGC | 1.00E+00 | -1.26E-02 | 1 | 47 | 9.13% | 3400.3 | 12.18% |
| Elk4(ETS)/Hela-Elk4-ChIP-Seq(GSE31477)/Homer | NRYTTCCGGY | 1.00E+00 | -9.68E-03 | 1 | 185 | 35.92% | 11424 | 40.91% |
| SpiB(ETS)/OCILY3-SPIB-ChIP-Seq(GSE56857)/Homer | AAAGRGGAAGTG | 1.00E+00 | -8.93E-03 | 1 | 66 | 12.82% | 4613.6 | 16.52% |
| GFX(?)/Promoter/Homer | ATTCTCGCGAGA | 1.00E+00 | -4.29E-03 | 1 | 2 | 0.39% | 412.9 | 1.48% |
| ETS1(ETS)/Jurkat-ETS1-ChIP-Seq(GSE17954)/Homer | ACAGGAAGTG | 1.00E+00 | -3.79E-03 | 1 | 272 | 52.82% | 16362.7 | 58.60% |
| ELF1(ETS)/Jurkat-ELF1-ChIP-Seq(SRA014231)/Homer | AVCCGGAAGT | 1.00E+00 | -1.31E-03 | 1 | 164 | 31.84% | 10659.2 | 38.17% |
| NRF(NRF)/Promoter/Homer | STGCGCATGCGC | 1.00E+00 | -1.11E-03 | 1 | 52 | 10.10% | 4073.5 | 14.59% |
| Elk1(ETS)/Hela-Elk1-ChIP-Seq(GSE31477)/Homer | HACTTCCGGY | 1.00E+00 | -9.62E-04 | 1 | 176 | 34.17% | 11390.4 | 40.79% |
| GABPA(ETS)/Jurkat-GABPa-ChIP-Seq(GSE17954)/Homer | RACCGGAAGT | 1.00E+00 | -3.63E-04 | 1 | 245 | 47.57% | 15350.4 | 54.97% |
| ETS(ETS)/Promoter/Homer | AACCGGAAGT | 1.00E+00 | -8.00E-06 | 1 | 95 | 18.45% | 7399.8 | 26.50% |
| REST-NRSF(Zf)/Jurkat-NRSF-ChIP-Seq/Homer | GGMGCTGTCCATGGTGCTGA | 1.00E+00 | 0.00E+00 | 1 | 0 | 0.00% | 172.5 | 0.62% |

**
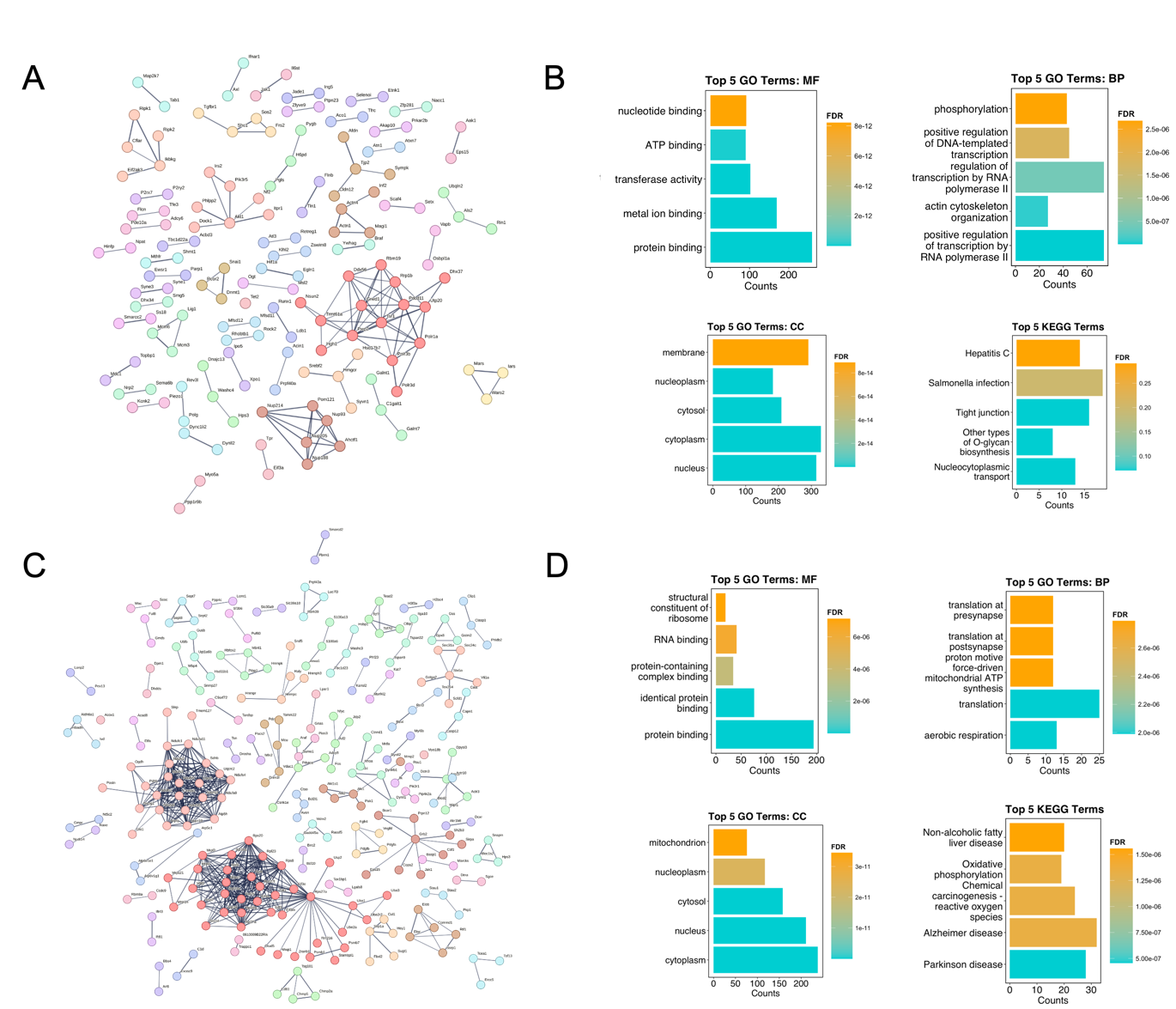
**

**Supplementary figure 1.** Summary of uniquely up- and downregulated DEGs by CBD. Unique upregulated (A) and downregulated (C) genes and their known protein-protein interactions, single nodes are not shown, highest confidence (0.9), clusters are coloured individually using K-means clustering. The top 5 Enriched KEGG pathways and GO terms for common upregulated (B) and downregulated genes (D). BP = biological process; CC = cellular compartment; MF = molecular function.

**
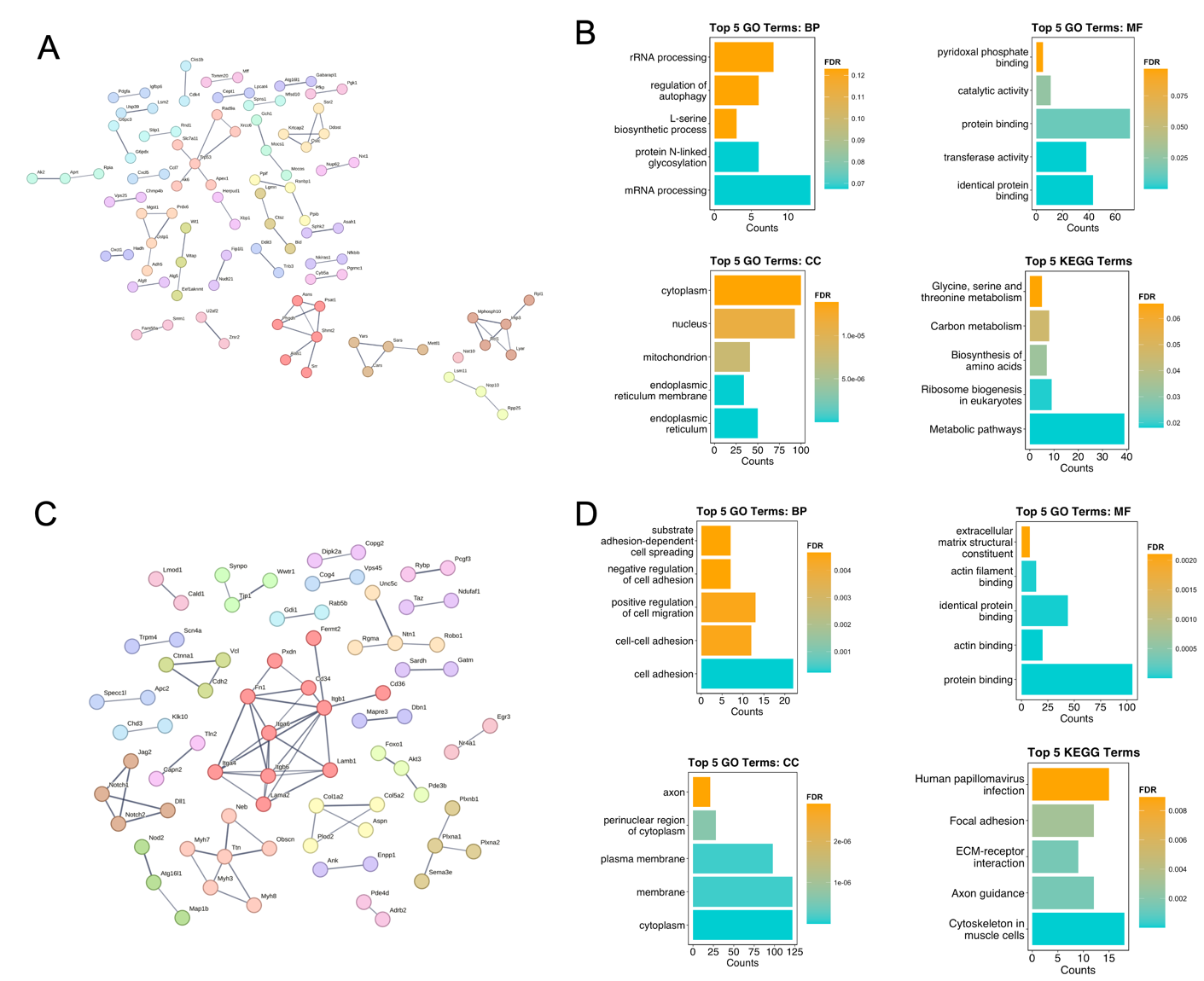
**

**Supplementary figure 2.** Summary of uniquely up- and downregulated DEGs by synthetic CBD. Unique upregulated (A) and downregulated (C) genes and their known protein-protein interactions, single nodes are not shown, highest confidence (0.9), clusters are coloured individually using K-means clustering. The top 5 Enriched KEGG pathways and GO terms for common upregulated (B) and downregulated genes (D). BP = biological process; CC = cellular compartment; MF = molecular function.
